# A Possible Aquatic Origin of the Thiaminase TenA of the Human Gut Symbiont *Bacteroides thetaiotaomicron*

**DOI:** 10.1007/s00239-023-10101-8

**Published:** 2023-04-06

**Authors:** Régis Stentz, Jitender Cheema, Mark Philo, Simon R. Carding

**Affiliations:** 1 Quadram Institute Bioscience, Gut Microbes and Health Research Programme, Norwich, UK; 2 Computational and Systems Biology, John Innes Centre, Norwich, UK; 3 Core Science Resources, Quadram Institute Bioscience, Norwich, UK; 4 Norwich Medical School, University East Anglia, Norwich, UK

**Keywords:** Horizontal gene transfer, Monophyletic group, Bacterial extracellular vesicles, Thiamin

## Abstract

TenA thiamin-degrading enzymes are commonly found in prokaryotes, plants, fungi and algae and are involved in the thiamin salvage pathway. The gut symbiont *Bacteroides thetaiotaomicron* (Bt) produces a TenA protein (BtTenA) which is packaged into its extracellular vesicles. An alignment of BtTenA protein sequence with proteins from different databases using the basic local alignment search tool (BLAST) and the generation of a phylogenetic tree revealed that BtTenA is related to TenA-like proteins not only found in a small number of intestinal bacterial species but also in some aquatic bacteria, aquatic invertebrates, and freshwater fish. This is, to our knowledge, the first report describing the presence of TenA-encoding genes in the genome of members of the animal kingdom. By searching metagenomic databases of diverse host-associated microbial communities, we found that BtTenA homologues were mostly represented in biofilms present on the surface of macroalgae found in Australian coral reefs. We also confirmed the ability of a recombinant BtTenA to degrade thiamin. Our study shows that Bt*tenA*-like genes which encode a novel sub-class of TenA proteins are sparingly distributed across two kingdoms of life, a feature of accessory genes known for their ability to spread between species through horizontal gene transfer.

**Supplementary Information:**

The online version contains supplementary material available at 10.1007/s00239-023-10101-8.

## Introduction

The gastrointestinal tract (GIT) of animals (Song et al. 2020) including insects (Jing 2020) worms (Zhang et al. 2017) and marine invertebrates (Liberti et al. 2021) accommodates a vast and complex community of microbes known as the microbiota. In humans, the GIT microbiota carries out vital functions for health including digestion, pathogen exclusion and immune function (Fan and Pedersen 2021). Recent evidence suggests that bacterial extracellular vesicles (BEVs) can contribute to interactions with other members of the microbial community and with host cells (Cecil et al. 2019; Caruana and Walper 2020). BEVs are nano-sized and non-replicative spherical particles which constitute a secretion system enabling the dissemination of membrane-encapsulated cellular materials including proteins, nucleic acids and metabolites into the extracellular milieu (Guerrero-Mandujano et al. 2017; Bryant et al. 2017) and beyond (Jones et al. 2020).

We have recently identified a set of proteins enriched in bacterial extracellular vesicles (BEVs) produced by the human gut-symbiont *Bacteroides thetaiotaomicron* (Bt) in vivo in the mouse intestine (Stentz et al. 2022). Amongst them is the BT_3146-encoded protein which is selectively secreted into BEVs indicating that this protein is of potential significance in host physiology. BT_3146 is annotated as being a member of the TenA protein family (Xu et al. 2003) and was defined as a transcriptional regulator (transcriptional enhancer A) based on observed changes in gene-expression patterns when overexpressed in *Bacillus subtilis* (Pang et al. 1991).

To date, TenA enzymes have been reported in prokaryotes, plants, fungi (Kraft and Angert 2017) and haptophyte algae (Gutowska et al. 2017), with the prototypical *Bacillus* spp. enzyme being described as the thiamin-degrading enzyme thiaminase II (Murata 1965). The hydrolysis of intact thiamin by the thiaminase II activity of diverse microbial TenA proteins has since then been demonstrated (Haas et al. 2005; Onozuka et al. 2008) and the three-dimensional structure resolved (Toms et al. 2005). In addition to their ability to catalyze the degradation of thiamin, TenA enzymes are involved in the thiamin salvage pathway, catalyzing the hydrolysis of 4-amino-5-aminomethyl-2- methylpyrimidine (HMP) precursors through TenA amino-HMP hydrolase activity in bacteria, yeast and plants (Jenkins et al. 2007; Onozuka et al. 2008; Zallot et al. 2014).

The TenA family has two subfamilies; TenA_C containing an active-site cysteine and TenA_E that lack the active-site cysteine (Zallot et al. 2014) totaling 39 TenA proteins from bacteria, archaea, plants and yeast. A phylogenetic analysis of the protein sequences showed that TenA_C and TenA_E classes fit into two distinct clades making the active-site cysteine residue a convenient marker that distinguishes the two subfamilies. A similar result was later obtained following phylogenetic analyses of the haptophyte algae TenA protein sequences with TenA_C and TenA_E proteins clustered in two distinct groups (Gutowska et al. 2017).

Here we demonstrate that based on sequence homology, BT_3146 belongs to a novel sub-class of TenA_C enzymes which now includes members of the animal kingdom and discuss the possible aquatic origin of this gene in Bt.

## Methods

### Phylogenetic Analysis

A protein BLAST (https://blast.ncbi.nlm.nih.gov/Blast.cgi) search of the BtTenA protein sequence against the nonredundant protein database was performed and 48 representative proteins selected. The 48 amino acid sequences (core) were aligned with PRANK (Löytynoja and Goldman 2010) and highly variable regions removed from the data set. The evolutionary history of BtTenA-like proteins was inferred first by fitting different models of evolution using the ModelTest-NG version 0.1.7 (Darriba et al. 2020) on the aligned core set of sequences. The consensus model of evolution using all three criteria namely, Akaike information criterion (AIC), corrected Akaike information criterion (AICc), and the Bayesian information criterion (BIC) was “WAG + I + G4” (Whelan and Goldman 2001). Next, the core sequence alignment was used to infer the maximum likelihood (ML) tree using IQ-TREE 1.6.12 (Nguyen et al. 2015) with the best-fitting model of amino acid evolution. Further, 1000 ultrafast bootstrap replicates (Minh et al. 2013) were drawn on the consensus tree. Finally, we rooted the tree at the midpoint and visualized it using the iTOL webservice (Letunic and Bork 2019).

To estimate the strength of evidence of horizontal gene transfer (HGT) which is the transfer of genes between species outside the normal mode of transmission from parent to offspring, we calculated Aggregate Hit Support (AHS) using AvP (Koutsovoulos et al 2022), which is a contamination-aware metric based on the normalized bitscores. Using AvP we also calculated outgroup percentage (*outg_pct*), which is the percentage of species from donor lineage in the top hits that have different taxonomic species name. The taxa ids were extracted after running the blastp (version 2.9.0 +) search against a remote nr database with parameters “ -outfmt '6 std staxids' -seg no -evalue 1e-5”. The code used and the associated data is available at (https://github.com/gitbackspacer/btenA). It is released under GNU General Public License v3.0.

### Sequence Retrieval and Associated Genomes

To retrieve BtTenA homologous protein sequences and associated microbial genomes, a BLASTP search was performed using the IMG /M data management and analysis system v.6.0 (Chen et al. 2021) at https://img.jgi.doe.gov/m/. The “gene orthologs neighborhoods” analysis was used to display an alignment of Bt*tenA*-like and surrounding genes from different bacterial genomes.

To retrieve protein sequences and associated genomes from host-associated microbial metagenomes, a BLASTP search was performed using the IMG /M data management and analysis system v.6.0 against the” host-associated metagenomes” databases.

### Bt-TenA In Vitro Expression and Thiaminase Activity

We used the *E. coli* cell-free protein synthesis (CFPS) system to produce recombinant active proteins in *vitro*. The primer pair 3146_BSA_fwd ATCGGTCTCCAATGAATGATTTCAAGAATCA and 3146_BSA_rev ATCGGTCTCCAAGCTCAGCCTTTCTTGTTCCAC was used to amplify the BT_3146 gene using Bt VPI-5482 chromosomal DNA as a template. The PCR fragment was inserted into the pEPQD0KN0025 target vector (Dudley et al. 2021) using the Golden Gate B*sa*I-based assembly protocol (Engler et al. 2008). As a control, the BT_1259 gene predicted to encode a choloylglycine hydrolase protein was amplified using the primer pair 1259_BSA_fwd ATCGGTCTCCAATGTGTACGAGAGTCGTTTA and 1259_BSA_rev ATCGGTCTCCAAGCTTATCCCTTTACGCCCATAA. The *E. coli* CFPS system was kindly provided by Dr. Quentin Dudley (Earlham Institute, Norwich, UK). 15 μg of total protein was added to Tris 10 mM pH 7.4 buffer containing 0.25 mM thiamin or 1 mM thiamin in a volume of 250 μl and the mixture was incubated for 3 h at 37 °C. Protein concentration was determined using the Bio-Rad protein assay with bovine serum albumin used as a standard.

Thiamin concentration was determined by HPLC–MS/MS using an Agilent 1200 HPPLC coupled to a Sciex 4000 QTrap triple quadrupole mass spectrometer. The sample was injected (0.5 µl) onto a Waters BEH Amide 150 × 2.1 × 1.7 µm column maintained at 40 °C operated in HILIC mode. Solvent A was 90% acetonitrile/water + 10 mM ammonium formate at pH 3.2 with formic acid and Solvent B was 50% acetonitrile/water + 10 mM ammonium formate at pH 3.2 with formic acid. The solvents were pumped at a constant flow of 0.4 ml/min. A binary gradient was applied as 0% B for 2.5 min, 100% B at 10 min and held for 5 min. The mass spectrometer was operated in positive electrospray mode with source conditions of curtain gas, GS1 and GS2 at 25, 50 and 20 L/min, respectively and source temperature of 550 °C. The mass analyser was operated in multiple reaction monitoring (MRM) mode to monitor the primary MRM transition of 265/122 amu declustering potential 90 V and collision energy 12 V and secondary ion of 265/81 declustering potential 90 V and collision energy 30 V, each with a dwell time of 150 ms. Under the conditions applied, thiamin had a retention time of 4.8 min.

## Results

### The Bt-TenA Protein Family Includes Members of the Animal Kingdom

From a previous comparative proteomic analysis of Bt BEVs and their parent cells that aimed to determine proteins selectively enriched in BEVs produced in the mouse GIT we identified the BT_3146 gene product annotated as “TenA” (Xu et al. 2003) with a 13-fold enrichment in BEVs produced in vivo versus BEVS produced in vitro (Stentz et al. 2022). We will refer to the BT_3146-encoded protein as BtTenA.

**Fig. 1:**
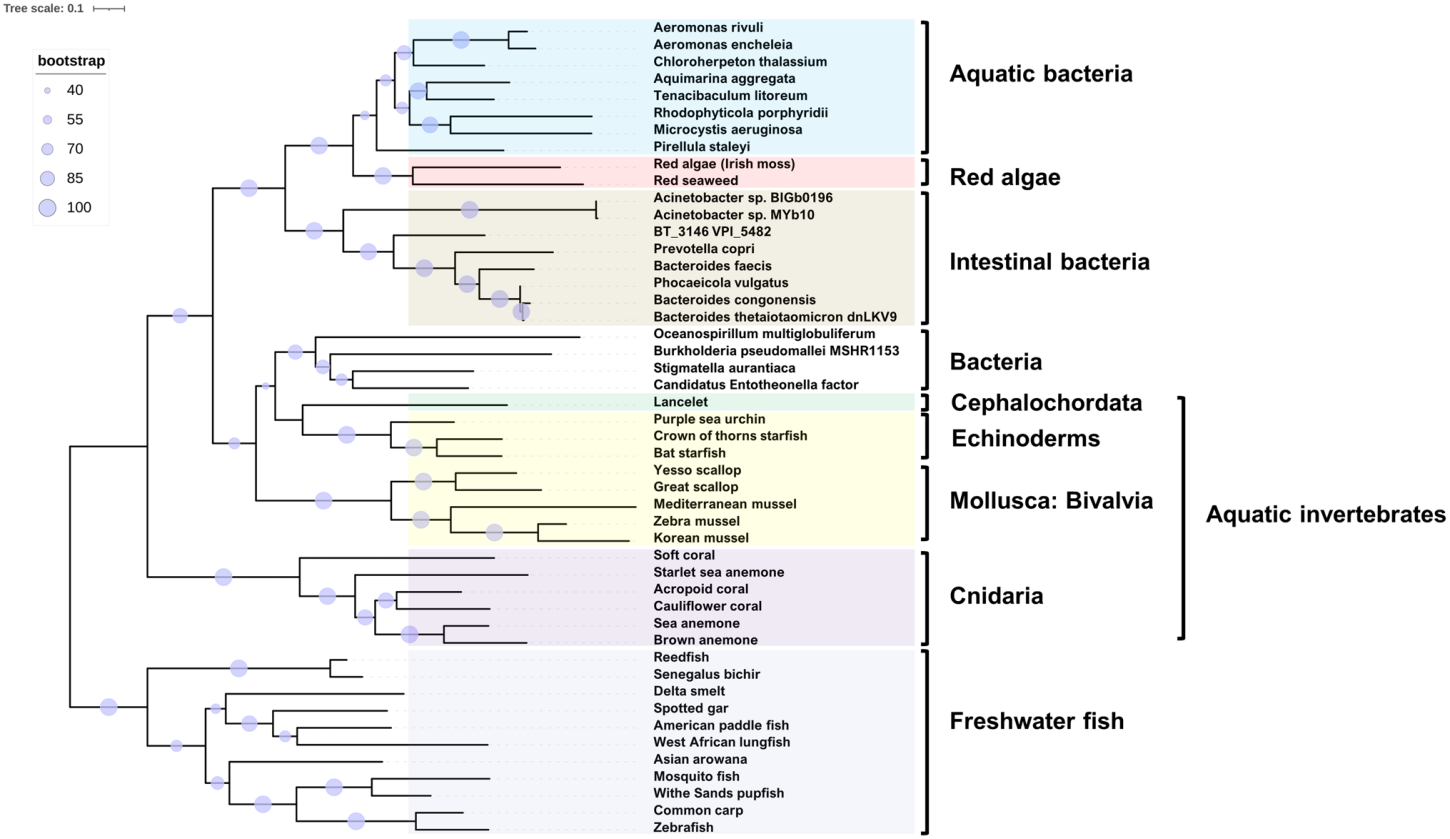
A maximum-likelihood phylogenetic tree derived from the alignment of 48 TenA protein representatives from different kingdoms of life was constructed in IQ-TREE using the model WAG + I + G4 with 1000 bootstrap replicates and visualized in iTOL. Bootstrap confidence is shown as percentages. Different clades have been assigned the following background colors: Aquatic bacteria, light blue; Intestinal bacteria, light brown; Red algae, pink; Echinoderms, mollusks, yellow; Freshwater fish, grey; Cnidaria, purple; Lancelet, green. The tree is drawn to scale, with branch lengths measured in the number of substitutions per site

A phylogenetic analysis was performed to determine the distribution of BtTenA-like homologues in different organisms. A BLASTP search of BtTenA-encoded protein sequence against the nonredundant protein database identified 194 sequences (Table S1) with significant alignments (E-value ≤ 10^–15^ and a minimal number of 50 identical amino acids over the entire sequence length). Amongst prokaryotes, Bacteroidetes residents of the lower GIT encoded proteins belonging to the BtTenA family although they were restricted to a small number of species (*Bacteroides* [*congonensis*, *faecis*, *thetaiotaomicron*], *Phocaeicola vulgatus* and *Prevotella copri*). BtTenA homologues were also identified in marine invertebrates and freshwater fish. A phylogenetic tree (Fig. 1) was constructed from the alignment of 48 representatives (Table S2) of the two kingdoms of life selected based on the diversity and the number of species represented, between and within the two kingdoms of life.

In general, TenA-related sequences were evolutionarily conserved within their respective clade and cluster according to the taxonomic group of the organism they belonged to. They were named according to the taxonomic group and their habitat.

#### Clade Intestinal Bacteria

The few TenAs from commensal GI *Bacteroides* spp., *P. vulgatus* and *P. copri* form a monophyletic group with two *Acinetobacter spp.* found in the natural microbiome of the nematode worm *Caenorhabditis elegans*: *Acinetobacter* sp. MYb10 (Taxon ID 2791355502) and *Acinetobacter* sp. BIGb0196 (Taxon ID 2824222098) at https://img.jgi.doe.gov/. This suggests that these BtTenA variants expressed by bacteria residing in the GIT of animals may share a common ancestor.

#### Clade Aquatic Bacteria

TenAs from GI bacteria cluster with TenAs of bacteria found in aquatic environments. Both *Aeromonas* spp. *A. rivuli* and *A. encheleia* originate from fresh water with *A. rivuli* found in a karst water rivulet (Figueras et al. 2011), and *A. encheleia* being associated with European eels reared in a freshwater farm (Esteve et al. 1996). All the other bacteria represented in this clade are of marine origin, including the green sulfur bacterium *Chloroherpeton* and the marine algal-associated bacteria *Rhodophyticola porphyridii* (Jung et al. 2019). Intriguingly, BtTenA-like proteins were found in two red algae with copies of the gene present in the genomes of the red algae Irish moss *Chondrus crispus* (Collén et al. 2013) and of the red seaweed *Gracilariopsis chorda* (Lee et al. 2018).

#### Clade Burkholderia (Other Bacteria)

Another clade contained bacteria of diverse origins including the sponge symbiont *Candidatus Entheonella* factor (Wilson et al. 2014), the marine bacterium *Oceanospirillum multiglobuliform, Stigmatella aurantiaca* found on rotting wood or associated with fungi, and the human and animal pathogen *Burkholderia pseudomallei* (28 hits) from which TenA from the human clinical isolate MSHR1153 was chosen as a representative. Weakly (bootstrap support of 45 and 44%), this clade tends to cluster with TenA representatives from marine invertebrates including chordates, echinoderms and mollusks with the exception of the zebra mussel (*Dreissena polymorpha*) which is found in freshwater habitats.

#### Clades Cnidaria and Freshwater Fish

BtTenA-like representatives were also predicted to be produced by marine cnidaria such as corals and sea anemones and a group of fish living in lakes or rivers.

### Could the BT_3145 Gene in Bacteroides spp. have been Acquired from Aquatic Bacteria by Horizontal Gene Transfer?

**Fig. 2:**
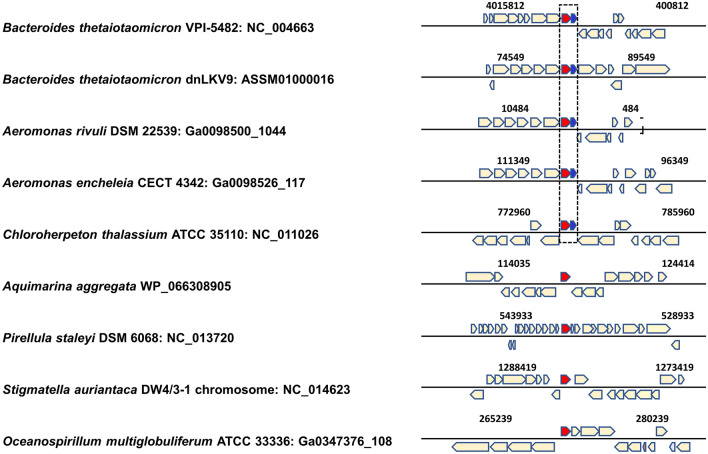
Neighborhood analysis of Bt-*tenA*-like genes from different bacteria. Bt*tenA* (BT_3146) homologues are labeled in red and BT_3145 homologues are in blue. Numbers corresponding to the coordinates of the genes on the different chromosomes are indicated

We next examined the genes adjacent to BT_3146 (encoding BtTenA) and compared them with those surrounding *tenA* in other bacterial species. By aligning the *tenA* genomic region of the different bacterial species at IMG (https://img.jgi.doe.gov/), we observed that Bacteroidetes species originating from the human intestine, i.e., *Bacteroides spp*., *P. vulgatus* and *P. copri*, all contained a gene predicted to encode a short protein (127–142 amino acids, Table 1) downstream from *tenA*. The gene product shares homology with BT_3145 from Bt (42–90%, Table 1). However, the other intestinal bacteria *Acinetobacter spp.* do not contain a copy of this gene. In contrast, within the “aquatic bacteria” clade, *A. rivuli*, *A. encheleia* and *C. thalassium* contain a BT_3145 homolog that is adjacent to the BT_3146-like protein (Fig. 2; Table 1). BT_3145 is annotated as being an “uncharacterized protein” and has no identifiable domain.

**Table 1:** BLASTP results against BT_3145

Organism	Phylum	Locus	Length	Score (maximum)	E-value	Identities
Bt VPI-5482	Bacteroidetes	NP_812057	142	297	8e-102	142/142(100%)
*Prevotella copri*	Bacteroidetes	MQN91271	142	271	4e-91	128/142(90%)
*Bacteroides congonensis*	Bacteroidetes	WP_022137009	127	101	2e-24	55/127(43%)
*Phocaeicola vulgatus*	Bacteroidetes	2,904,900,433	127	97.8	4e-23	54/127(43%)
*Bt dnLKV9*	Bacteroidetes	2,565,668,743	127	97.4	4e-23	54/127(43%)
*Bacteroides faecis*	Bacteroidetes	WP_156730477	127	94	1e-25	53/127(42%)
*Chloroherpeton thalassium*	Chlorobiota	WP_012499221	125	75	5e-14	48/128(38%)
*Aeromonas encheleia*	Proteobacteria	WP_042653069	125	67	2e-13	43/128(34%)
*Aeromonas rivuli*	Proteobacteria	WP_042041974	125	49.3	9e-07	44/128(34%)

To investigate further the possible acquisition of the Bt*tenA* gene from aquatic bacteria, potential horizontal gene transfer (HGT) events were assessed using the AvP pipeline (see Methods). We have obtained high Aggregate Hit Support (AHS) values of 561.0 and 310.7 for *Aquimarina aggregata* (gene product WP_066308905.1) and *Aeromonas rivuli* (gene product WP_224431244.1), respectively. These high values are an indicative of these aquatic bacterial species (Fig. 1, Table S2) to be the likely donors to the *B. thetaiotaomicron* VPI-5482 recipient strain. However, although both species have a comparable AHS metric and an outgroup percentage outg_pct around 95%, our gene synteny comparison suggests that *A. rivuli* is more likely to be the donor as a copy of the BT_3145 gene is found adjacent to the Bt*tena*-like gene only in this species which is not the case for *A. aggregata* (Fig. 2).

### Bt-TenA Homologue Proteins are Highly Represented in Coral Reef Ecosystems

With the aim of identifying BtTenA bacterial members in diverse environments, we performed a BLASTP search against metagenomic databases found in IMG. Not surprisingly, BtTenA or BtTenA homologues were found in metagenomic datasets of the human distal GI microbiome (Table S3). However, our database search also revealed that the habitat comprising the most BtTenA representatives was the surface of macroalgae (*Sargassum* sp.) which includes six samples collected at fringing reefs surrounding the Magnetic Island (Geoffrey Bay), Queensland, Australia (Glasl et al. 2019) (Table 2). BtTenA homologues were also found in host-associated microbial community of a marine sponge (Table S3).

**Table 2:**
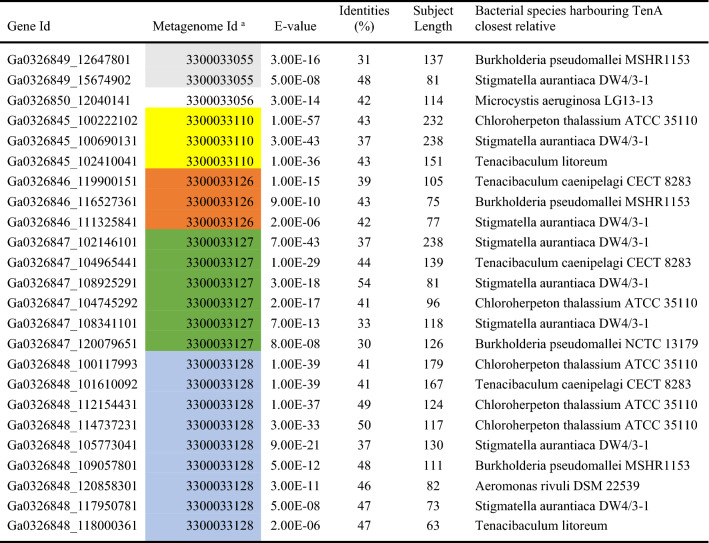
Bt-TenA homologue proteins are highly represented in coral reef ecosystems

BLASTP results against metagenomics databases (Macroalgae surface microbial communities from Magnetic Island Reef, Townsville, Australia)

^a^Metagenomes from macroalgae surface microbial communities from Magnetic Island Reef, Townsville, Australia. The colors highlight groups of the same sample

The closest relatives to BtTenA identified in this biofilm ecosystem were found in the predicted proteomes of the marine bacteria *C. thalassium*, *Tenacibaculum litoreum* and *Tenacibaculum caenipelagi*, the freshwater bacterium *A. rivuli*, the wood and fungi-associated bacteria *S. aurantiaca,* and the human and animal pathogen *B. pseudomallei*.

### The Thiaminase Activity of Bt-TenA

We investigated the potential thiaminase activity of BtTenA and its 47 relatives from their alignment and assessed its enzymatic activity in vitro.

The active-site cysteine residue identified in the bacterium *B. subtilis* TenA (Cys135) (Jenkins et al. 2008) was conserved in BtTenA-like proteins (Cys164 in BtTenA, Fig. S) 48 representatives (Fig. 1; Table S2) except for TenA from the brown anemone which instead contains a serine residue at this position (Fig. S1). BtTenA and BtTenA-like sequences have only one of the double glutamate residues identified in the active site of TenA from *Bacillus subtilis* (Glu 205) (Jenkins et al. 2008), as previously observed for a few members of the TenA_E and TenA_C subfamilies (Zallot et al 2014). The glutamate residue corresponding to Glu 205 in *B. subtilis* (Glu 229 in *Bt*) was consistently conserved in BtTenA-like proteins with the exception of the cyanobacterium *Cyanomargarita calcarean* sequence containing an aspartate residue instead. An alignment of the BtTenA-like proteins with members of the TenA_E and TenA_C subfamilies confirmed that BtTenA members are distinct from the 2 other groups and constitute a novel TenA sublineage (Fig. S2).

**Fig. 3:**
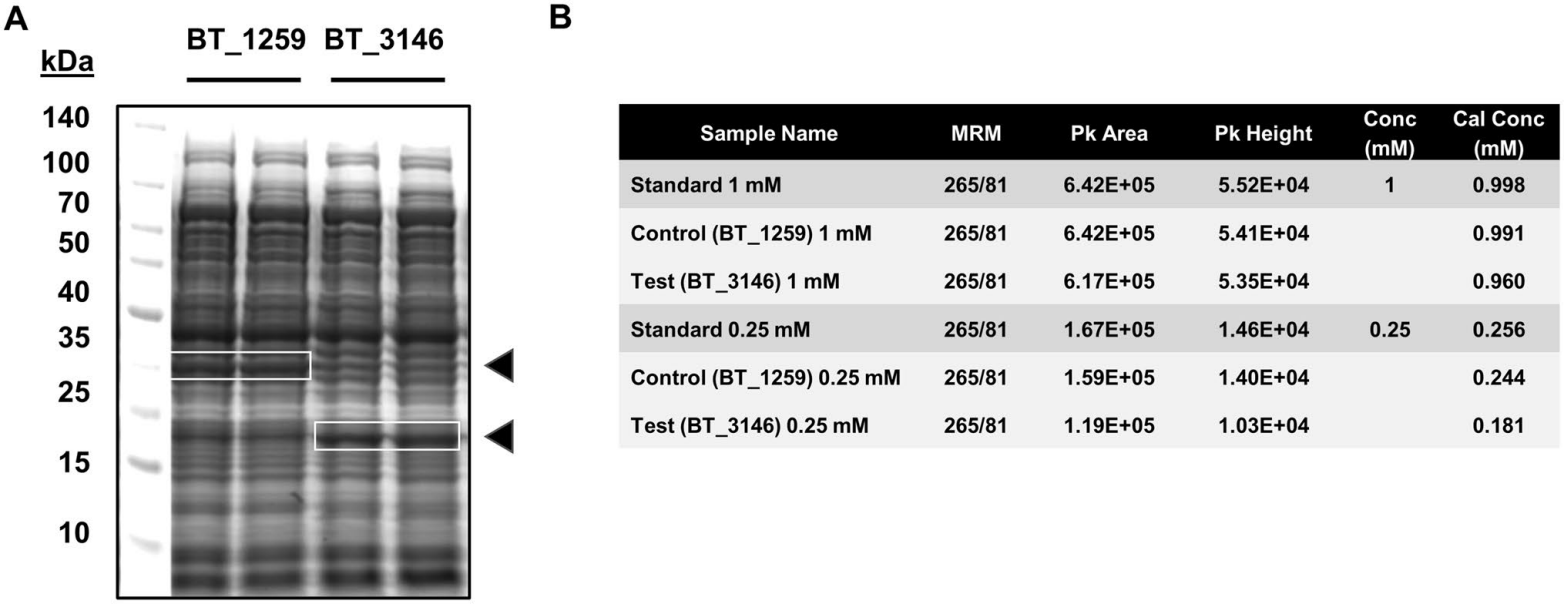
Thiaminase activity of BtTenA. **A** In vitro expression of BtTenA (BT_3146) using the *E. coli Escherichia coli* CFPS system. Expression of BT_1259 was used as a negative control. Two samples for each protein are shown. **B** Thiaminase activity of BT_3146. The protein extracts containing BT_3146 or BT_1259 were incubated with thiamin at the indicated concentration for 3 h at 37 °C. The secondary channel (265/81) complemented the results of the main channel (265/22)

We confirmed the thiaminase activity of BtTenA by comparing the degradation of thiamin from *E. coli* cell-free protein synthesis (CFPS) extract containing recombinant BtTenA (Fig. 3) with CFPS containing BT_1259 (choloylglycine hydrolase) used as a control. The activity measured was 5.5 ± 1.4 mU (nmole thiamin consumed.min^−1^)/mg total protein with some thiaminase activity detected in the control (0.72 ± 0.08 mU/mg protein), likely due to thiaminase activity present in the *E. coli* protein extract.

## Discussion

Our BtTenA phylogenetic analysis highlights a new subclass of TenA_C proteins which, in addition to those previously described, includes members of the animal kingdom. Given the more substantial representation of genes encoding BtTenA-like proteins in aquatic environments, we propose that the genes found in Bt and other members of the animal intestinal microbiota derive from aquatic environments. Furthermore, the detection of horizontal gene transfers using the AvP pipeline combined with a comparison of Bt*tenA*-like neighboring genes in different species identified the aquatic bacterium *A. rivuli* as a potential Bt*tenA* donor to the intestinal bacterium Bt VPI-5482.

Another example of the acquisition of genes by members of the human intestinal microbiota is the theoretical transfer of porphyranase and agarase genes from a marine bacterium to the GI symbiont *Bacteroides plebeius*, most probably by horizontal gene transfer (HGT) (Hehemann et al. 2010). Interestingly, the route by which these enzymes were acquired in human gut bacteria may have been dietary seaweeds (nori) associated with marine bacteria and are consumed in sushi (Hehemann et al. 2010). To support this finding, comparative gut metagenome analyses showed that porphyranases and agarases were found frequently in the human Japanese population, with sushi being a widely and frequently consumed food in Japan, and its notable absence in metagenome datasets derived from North American individuals. It is tempting to speculate that likewise, *tenA* of intestinal bacteria was acquired from the consumption of aquatic organisms to which *tenA*-containing bacteria were associated.

In the same way, our comparative microbial host-associated metagenome analyses showed that BtTenA homologues are frequently found in marine environments. This includes macroalgal biofilms found in coral reefs that are composed of microbial communities containing the highest number of copies (complete or partial, depending on the outcome of the genome assembly) of BtTenA-like products (Table 2). Similar results (Table S3) were observed for microbial coral communities isolated from coral reefs in Mexico and Australia (Coral microbial communities from various locations to study host-microbial communication, IMG Metagenome IDs: 3300010030-5, 3300022597-9 and 3300022600). These results support the possible aquatic origin of the *tenA* gene which is also linked to algal and marine invertebrate species, as also reflected by the possible HGT that occurred between marine bacteria and red algae (Fig. 1).

Phylogenetic analysis placed red alga TenAs in the clade of bacterial TenAs. The presence of copies of Bt-TenA-like gene products in Irish moss red algae and red seaweed is likely to be the result of HGT from marine bacteria such as *Rhodophyticola porphyridii* (associated with red algae) (Jung et al. 2019) to algae. Furthermore, among the 9 genomes of Florideophyceae (sub-class Rhodymeniophycidae) red algae in the NCBI database (https://www.ncbi.nlm.nih.gov/datasets/genomes/) only *Chondrus crispus* (Irish Moss) and *Gracilariopsis chorda* (red seaweed) contain a copy of a Bt-TenA homologue, indicating that the transfer is likely to have occurred after the emergence of the common ancestor of the Rhodymeniophycidae sub-class (Yang et al. 2016). In a previous work, HGT of genes involved in the biosynthesis of terpenes is proposed to have occurred from bacteria to red algae (Wei et al. 2019). The possibility that terpene synthase genes in red algae are acquired from bacteria was inferred from phylogenetic analyses resulting in the clustering of terpene enzymes with bacterial terpene synthases. More generally, HGT was described as a mechanism responsible for the origin of a significant proportion of nuclear genes of sequenced red algal genomes (Bhattacharya et al. 2018). At present, we cannot rule out the possibility that the absence of TenA genes in the seven other red algae is due to *tenA* gene loss. An improved understanding of the presence and absence of TenA genes in red algae will rely on continued genome and transcriptome sequencing of diverse red algal species (Wei et al. 2019).

It should be noted that the outcome of our initial BLASTP search against BtTenA displayed only one representative of the Firmicutes (*Lachnospiraceae bacterium*, found in the ruminant gastrointestinal microbiome, accession JAFUZE000000000), a major phylum of the human GIT microbiota. However, although it clustered with the group of intestinal bacteria in phylogenetic trees (data not shown), TenA from *L. bacterium* appeared to be more distantly related to TenAs from other bacterial species since it showed an early diverging lineage. The clustering of *L. bacterium* within the bacteria clade lacked support at the basal node and was therefore not included in the tree displayed in Fig. 1.

Amongst the group of bacteria for which BtTenA homologs form a monophyletic group is *B. pseudomallei* (Fig. 1), a primary pathogen of animals and humans (Syed and Wooten 2021.). Intriguingly, it is the only *Brukholderia* species with *B. mayonis* and *B. oklahomensis* that contains a copy of the Bt*tenA*-like gene (Table S1) in its genome. The *tenA* neighboring genes and their organization is highly similar for 66 strains (including *B. pseudomallei* MSHR1153) and for a second group composed of 6 strains (including *B. pseudomallei* VB976100) (Fig. S3) which suggests a HGT from a bacterium to a common ancestor of each of these species, after the *Burkholderia* lineage emerged. Close relatives of *Burkholderia* TenA were also identified in bacteria found in the bacterial community forming biofilms on the surfaces of macroalgae (Table 2). In a recent study aiming to identify bacterial communities associated with sponges in the Bay of Bengal, Paul et al. (2021) found that *Burkholderia* spp. were largely represented which may explain the presence and origin of TenA in pathogenic species of *Burkoldheria*.

Although part of the clade including intestinal bacteria (Fig. 1), the gene encoding TenA of both *Acinetobacter* species is not adjacent to a BT_3145 homologous gene (nor elsewhere in the genome) which contrasts with Bacteroidetes species. Since all these TenA variants share a common ancestor, it is possible that *tenA* in intestinal *Acinetobacter* spp. are acquired from intestinal Bacteroidetes members and the transfer somehow occurred with a loss of the BT_3145 gene. However, the possible loss of the BT_3145-like gene by *Acinetobacter* species after the transfer occurred cannot be excluded.

Among the 3,461 fish genomes available in the NCBI genome database (at 06.30.2022), (https://www.ncbi.nlm.nih.gov/datasets/genomes/?taxon=7898) we selected the 11 species containing a gene product highly homologous to BtTenA (BLASTP E-value ≤ 10^–15^). The search identified 115 proteins in total from genomes of diverse fish species either from marine or freshwater environments (BLASTP E-value ≤ 0.05) sharing homology with BtTenA. We believe that this is the first time that the presence of a TenA protein/gene in the genome of members of the animal kingdom has been reported.

In summary, our study reveals that *tenA*-like genes are widespread across all kingdoms of life with BtTenA being sparingly distributed among bacterial and animal species reminiscent of bacterial accessory genes which are often subject to HGT (Young 2016). How BtTenA relatives contribute to the fitness of their hosts and to what extent the thiaminase II activity and/or thiamin salvage pathway is involved remains to be determined. Similarly, the role of BtTenA in BEVs produced in the lower GI-tract of animals (Stentz et al. 2022) in host metabolism also warrants further investigations.

## Supplementary Information

Below is the link to the electronic supplementary material﻿.

Supplementary file1 (PDF 1259 kb)

Supplementary file2 (XLSX 22 kb)
