# Supplementary material for: A Possible Aquatic Origin of the Thiaminase TenA of the Human Gut Symbiont Bacteroides thetaiotaomicron
Source: J Mol Evol. 2023 Apr 6;91(4):482–91. doi: 10.1007/s00239-023-10101-8 (PMC10277260; doi:10.1007/s00239-023-10101-8)
Supplement: Supplementary file 1 — Supplementary file1 (PDF 1259 kb) [file 239_2023_10101_MOESM1_ESM.pdf]

A possible aquatic origin of the thiaminase TenA of the human gut symbiont *Bacteroides thetaiotaomicron*

Régis Stentz, Jitender Cheema, Mark Philo, Simon R. Carding

Quadram Institute Bioscience, Norwich, UK

Corresponding author(s). Email(s): regis.stentz@quadram.ac.uk

Table S1: List of the 194 sequences with significant alignments (E-value ≤ 10^{-15} and a minimal number of 50 identical amino acids over the entire sequence length) obtained from a BLASTP search of the BtTenA-encoded protein sequence against the nonredundant protein database

>WP_011108665.1 MULTISPECIES: hypothetical protein [Bacteroidales]

MNDFKNQWLRKRTFAIPASRLTGRLTTLKSDVPAADSLFWKLWNGSLDTAVQVLQTDYFKGIAAGTLDPNAYGSLMVQDGYYCFRGRDDYATAATCAQDETLREFFK
AKAKSYDEYNETYHQTWHLREASGLIPGTDIKDYADYEAYVAGSLASPYMCVVMLPCEYLWPWIANFLDGYTPTNSLYRFWIEWNGGTPNGAYQMGNMLEQYRDKID
EDKAVEIFNTAMNYELKVFTSSTILTTIENGK

>AAO78252.1 TenA family transcriptional activator-like protein [Bacteroides thetaiotaomicron VPI-5482]

MNDFKNQWLRKRTFAIPASRLTGRLTTLKSDVPAADSLFWKLWNGSLDTAVQVLQTDYFKGIAAGTLDPNAYGSLMVQDGYYCFRGRDDYATAATCAQDETLREFFK
AKAKSYDEYNETYHQTWHLREASGLIPGTDIKDYADYEAYVAGSLASPYMCVVMLPCEYLWPWIANFLDGYTPTNSLYRFWIEWNGGTPNGAYQMGNMLEQYRDKID
EDKAVEIFNTAMNYELKVFTSSTILTTIENGK

>2A2M_A CRYSTAL STRUCTURE OF a putativeTenA family transcriptional regulator (BT_3146) FROM BACTEROIDES THETAIOTAOMICRON VPI-5482 AT 1.88 A RESOLUTION [Bacteroides thetaiotaomicron VPI-5482]

XGSDKIHHHHHHXNDFKNQWLRKRTFAIPASRLTGRLTTLKSDVPAADSLFWKLWNGSLDTAVQVLQTDYFKGIAAGTLDPNAYGSLXVQDGYYCFRGRDDYATAAT
CAQDETLREFFKAKAKSYDEYNETYHQTWHLREASGLIPGTDIKDYADYEAYVAGSLASPYXCVVXLPCEYLWPWIANFLDGYTPTNSLYRFWIEWNGGTPNGAYQXG
NXLEQYRDKIDEDKAVEIFNTAXNYELKVFTSSTILTTIENGK

>WP_195662386.1 hypothetical protein [Bacteroides congonensis]

MDMNKWYIRRDFSVVAERLGRRGQLLSSDAPSPDALFWEMWQECEPIARQVLETAYFKGILNNDLDPNAYGTLMVQDAYYCFKAQDAYAVAATHALDDACGDFLQG
KYTSYEEYNAYYHETWHVREASGVIPGDEIKEYAAYEAFVAGNLDSPYLFSVMLPCEYLWNWIANELDKTAPKDGLYYFWIEGNGGTPDGAYQMANMLESYRRQIDE
AKAKEIFRIALQHELKVFTAATLLKSELLWQRKNSSLQR

>WP_073346898.1 hypothetical protein [Bacteroides congonensis]

MDMNKWYIRRDFSVVAERLGRRGQLLSSDAPPPDALFWEMWQECEPIARQVLETAYFKGILNNDLDPNAYGTLMVQDAYYCFKAQDAYAVAATHALDDACGDFLQG
KYTSYEEYNAYYHETWHVREASGVIPGDEIKEYAAYEAFVAGNLDSPYLFSVMLPCEYLWNWIANELDKTAPKDGLYYFWIEGNGGTPDGAYQMANMLESYRRQIDE
AKAKEIFRIALQHELKVFTAATLLKSELLWQRKNSSLQR

>MCD7962432.1 hypothetical protein [Rikenellaceae bacterium]

MKRYYYPKRDFSIPESLLTGYLSNVIGDRPSDDALFWEMWNECQSIADAVLQTDYFKGIVNANLHPAAFGSLMVQDAYYCMKGRDDYSAAVTHALDDKCKEFMQRKV
DSYDNYNVYYHKTWHIREAQSIIPGKEIKDYADYEAYVAGNLESPYVFCVMLPCEYLWNWVANQIDDSVPAGTIYRFWVDSNRGEPTGGYQMANILEDYRSQINESKA
KEIFKKAMNYELEVFTASTKIDKLWEKK

>WP_195406687.1 hypothetical protein [Bacteroides congonensis]

MDMNKWYIRRDFSVVAERLGRRGQLLSSDAPPPDALFWEMWQECEPIARQVLETAYFKGILNNDLDPNAYGSLMVQDAYYCFKAQDAYAVAATHALDDACGDFLQG
KYTSYEEYNTYYHEAWHVREASGVIPGDEIKEYAAYEAFVAGNLDSPYLFSVMLPCEYLWNWIANELDKTAPKDGLYYFWIEGNGGTPDGAYQMANMLESYRRQIDE
AKAKEIFRIALQHELKVFTAATLLKSELLWQRKNSSLQR

>CDA87108.1 tenA family transcriptional activator-like protein [Bacteroides sp. CAG:754]

MDMNKWYIRRDFSVVAERLGRRGQLLSSDAPPPDALFWEMWQECEPIARQVLETAYFKGILNNDLDPNAYGSLMVQDAYYCFKAQDAYAVAATHALDDACGDFLQG
KYTSYEEYNAYYHETWYVREASGVIPGDEIKEYAAYEAFVAGNLDSPYLFSVMLPCEYLWNWIANELDKTAPKDGLYYFWIEGNGGTPDGAYQMANMLESYRRQIDE
AKAKEIFRIALQHELKVFTAATLLKSELLWQRKNSSLQR

>WP_227188819.1 hypothetical protein [Bacteroides thetaiotaomicron]

MNKWYIRRDFSVAAEKLGRRGQLLSSDAPPPDALFWEMWQECEPIARQVLETAYFKGILNNDLDPNAYGALMVQDAYYCFKAQDAYAVAATHALDDACGDFLQGKY
TSYEEYNAYYHETWHVREASGVIPGDEIKEYAAYEAFVAGNLDSPYLFSVMLPCEYLWNWIANELDKTAPKDGLYYFWIEGNGGTPDGAYQMANMFEIYRRQIDEAKA
KEIFRIALQHELKVFTATLLKSELLWQRKNSSLQR

>MBV4310778.1 hypothetical protein [Bacteroides thetaiotaomicron]

MDMNKWYIRRDFSVAAEKLGRRGQLLSSDAPPPDALFWEMWQECEPIARQVLETAYFKGILNNDLDPNAYGALMVQDAYYCFKAQDAYAVAATHALDDACGDFLQG
KYTSYEEYNAYYHETWHVREASGVIPGDEIKEYAAYEAFVAGNLDSPYLFSVMLPCEYLWNWIANELDKTAPKDGLYYFWIEGNGGTPDGAYQMANMFEIYRRQIDEA
KAKEIFRIALQHELKVFTAATLLKSELLWQRKNSSLQR

>WP_229532965.1 hypothetical protein [Phocaeicola vulgatus]

MNKWYIRRDFSVAAERLGRRGQFLSSDAPPPDALFWEMWQECEPIARQVLETAYFKGILNNDLDPNAYGALMVQDAYYCFKAQDAYAAAATHPLDDACGDFLQGKYT
SYEEYNAYYHETWHVREASGVIPGDEIKEYAAYEAFVAGNLDSPYLFSVMLPCEYLWNWIANELDKTAPKDGLYYFWIEGNGGTPDGAYQMANMLESYRRQIDEAKA
KEIFRIALQHELKVFTTATLLKSELLWQRKNSILQR

>WP_229106048.1 hypothetical protein [Phocaeicola vulgatus]

MNKWYIRRDFSVAAERLGRRGQFLSSDAPPPDALFWEMWQECEPIARQVLETAYFKGILNNDLDPNAYGALMVQDAYYCFKAQDAYAAAATHALDDACGDFLQGKY
TSYEEYNAYYHETWHVREASGVIPGDEIKEYAAYEAFVAGNLDSPYLFSVMLPCEYLWNWIANELDKTAPKDGLYYFWIEGNGGTPDGAYQMANMLESYRRQIDEEKA
KEIFRIALQHELKVFTAATLLKSELLWQRKNSILQR

>WP_234128484.1 hypothetical protein [Bacteroides thetaiotaomicron]

MNKWYIRRDFSVAAERLGRRGQFLSSDAPPPDALFWEMWQECEPIARQVLETAYFKGILNNDLDPNAYGALMVQDAYYCFKAQDAYAAAATHALDDACGDFLQGKY
TSYEEYNAYYHETWHVREASGVIPGDEIKEYAAYEAFVAGNLDSPYLFSVMLPCEYLWNWIANELDKTAPKDGLYYFWIEGNGGTPDGAYQMANMLESYRRQIDEEKA
KEIFRIALQHELKVFTTATLLKSELLWQRKNSSLQR

>WP_229508550.1 hypothetical protein [Phocaeicola vulgatus]

MNMNKWYIRRDFSVAAERLGRRGQFLSSDAPPPDALFWEMWQECEPIARQVLETAYFKGILNNDLDPNAYGALMVQDAYYCFKAQDAYAAAATHPLDDACGDFLQG
KYTSYEEYNAYYHETWHVREASGVIPGDEIKEYAAYEAFVAGNLDSPYLFSVMLPCEYLWNWIANELDKTAPKDGLYYFWIEGNGGTPDGAYQMANMLESYRRQIDE
AKAKEIFRIALQHELKVFTTATLLKSELLWQRKNSILQR

>MCE9236248.1 hypothetical protein [Bacteroides thetaiotaomicron]

MNMNKWYIRRDFSVAAERLGRRGQFLSSDAPPPDALFWEMWQECEPIARQVLETAYFKGILNNDLDPNAYGALMVQDAYYCFKAQDAYAAAATHALDDACGDFLQG
KYTSYEEYNAYYHETWHVREASGVIPGDEIKEYAAYEAFVAGNLDSPYLFSVMLPCEYLWNWIANELDKTAPKDGLYYFWIEGNGGTPDGAYQMANMLESYRRQIDEE
KAKEIFRIALQHELKVFTTATLLKSELLWQRKNSSLQR

>MBO5156346.1 hypothetical protein [Prevotella sp.]

MNMMWYKKRSFAIPTMRLGKMALSALADEPAEDSLFKEMWNECKSIAQDVLATDYFKGILNNNLDPNAYGSLMVQDAYYCFKAQDSYAAAATHAMDEDCEKFMQA
KFDSYAEYNKYYHNPWCVREASGVIPGNAIKTYADYEAYVAQHLDSPYVFAVMLPCEYLWNWVANELAKTADPKGLYYFWIEGNGGKPEGAYQMAAMLERYRNRID
EAKAKEIFRTAMEHEKEVFTCATKLKYQQTKNI

>WP_134855275.1 hypothetical protein [Phocaeicola vulgatus]

MNMNKWYIRRDFSVAAERLGRRGQFLSSDAPPPDALFWEMWQECEPIARQVLETAYFKGILNNDLDPNAYGALMVQDAYYCFKAQDAYAAAATHPLDDACGDFLQG
KYTSYEEYNAYYHETWHVREASGVIPGDEIKEYAAYEAFVAGNLDSPYLFSVMLPCEYLWNWIANELDKTAPKDGLYYFWIEGNGGIPDGAYQMANMLESYRRQIDEA
KAKEIFRIALQHELKVFTAATLLKSELLWQRKNSILQR

>WP_230475135.1 hypothetical protein [Bacteroides thetaiotaomicron]

MNKWYIRRDFSVAAERLGRRGQFLSSDAPPPDALFWEMWQECEPIARQVLETAYFKGILNNDLDPNAYGALMVQDAYYCFKAQDAYAAAATHALDDACGDFLQGKY
TSYEEYNAYYHETWHVREASGVIPGDEIKEYAAYEAFVAGNLDSPYLFSVMLPCEYLWNWIANELDKTAPKDGLYYFWIEGNGGIPDGAYQMANMLESYRRQIDEAKA
KEIFRIALQHELKVFTAATLLKSELLWQRKNSILQR

>EOR97517.1 hypothetical protein C799_04352 [Bacteroides thetaiotaomicron dnLKV9]

MNMNKWYIRRDFSVAAERLGRRGQFLSSDAPPPDALFWEMWQECEPIARQVLETAYFKGILNNDLDPNAYGALMVQDAYYCFKAQDAYAAAATHALDDACGDFLQG
KYTSYEEYNAYYHETWHVREASGVIPGDEIKEYAAYEAFVAGNLDSPYLFSVMLPCEYLWNWIANELDKTAPKDGLYYFWIEGNGGIPDGAYQMANMLESYRRQIDEA
KAKEIFRIALQHELKVFTAATLLKSELLWQRKNSILQR

>MCE8942323.1 hypothetical protein [Bacteroides faecis]

MNKWYIRRDFSVAAAKMGKRGIALSSDEPPADALFWEMWNECEDIARQVLDTDYFRGIRNNNLDPNAYGSLMVQDAYYCFEAENAYAAAASHPLDDVCSDFLKGKCA
SYEEYNLYYHGAWHIHDASGVIPGDPIKSYADYEAHVAGHLDSPYLFCVMLPCEYLWNWIANQLLPTASPDGLYYFWIEGNGGTPDGAYQMANMLENYRTQMDEKQA
KEIFHTAMRHELEVFSTATELKNNALWLRKNTF

>WP_227034653.1 hypothetical protein [Bacteroides faecis]

MTLMNKWYIRRDFSVAAAKMGKRGIALSSDEPPADALFWEMWNECEDIARQVLDTDYFRGIRNNNLDPNAYGSLMVQDAYYCFEAENAYAAAASHPLDDVCSDFLKG
KCASYEEYNLYYHGAWHIHDASGVIPGDPIKSYADYEAHVAGHLDSPYLFCVMLPCEYLWNWIANQLLPTASPDGLYYFWIEGNGGTPDGAYQMANMLENYRTQMDE
KQAKEIFHTAMRHELEVFSTATELKNNALWLRKNTF

>WP_192915911.1 hypothetical protein [Bacteroides faecis]

MTLMNKWYIRRDFSVAAAKMGKRDIALSSGEPPADALFWEMWNECEDIARQVLDTDYFRGIRDNNLDPNAYGSLMVQDAYYCFEAENAYAAAASHPLDDVCSDFLKG
KCASYEEYNLYYHGPWHIRDASGVIPDDPIKSYADYEAHVAGHLDSPYLFCVMLPCEYLWNWIANQLLPTASPDGLYYFWIEGNGGTPDGAYQMANMLENYRTQTDEE
QAKEIFHTAMRHELEVFSTATQLNTNALWLRKNTF

>KAA5267574.1 hypothetical protein F2Z41_13865 [Bacteroides faecis]

MNKWYIRRDFSVAAAKMGKRDIALSSGEPPADALFWEMWNECEDIARQVLDTDYFRGIRDNNLDPNAYGSLMVQDAYYCFEAENAYAAAASHPLDDVCSDFLKGKCA
SYEEYNLYYHGPWHIRDASGVIPDDPIKSYADYEAHVAGHLDSPYLFCVMLPCEYLWNWIANQLLPTASPDGLYYFWIEGNGGTPDGAYQMANMLENYRTQTDEEQAK
EIFHTAMRHELEVFSTATQLNTNALWLRKNTF

>KAA5274630.1 hypothetical protein F2Z14_10170 [Bacteroides faecis]

MNKWYIRRDFSVAAAKMGKRGIALSSDEPPADALFWEMWNECEDIARQVLDTDYFRGIRNNNLDPNAYGSLMVQDAYYCFEAENAYAAAASHPLDDVCSDFLKGKCA
SYEEYNLYYHGPWHIRDASGVIPDDPIKSYADYEAHVAGHLDSPYLFCVMLPSEYLWNWIANQLLPTASPDGLYYFWIEGNGGTPDGAYQMANMLENYRTQTDEEQAK
EIFHTAMRHELEVFSTATQLNTNVLWLRKNTF

>WP_168166613.1 MULTISPECIES: hypothetical protein [Bacteroides]

MTLMNKWYIRRDFSVAAAKMGKRGIALSSDEPPADALFWEMWNECEDIARQVLDTDYFRGIRNNNLDPNAYGSLMVQDAYYCFEAENAYAAAASHPLDDVCSDFLKG
KCASYEEYNLYYHGPWHIRDASGVIPDDPIKSYADYEAHVAGHLDSPYLFCVMLPSEYLWNWIANQLLPTASPDGLYYFWIEGNGGTPDGAYQMANMLENYRTQTDEE
QAKEIFHTAMRHELEVFSTATQLNTNVLWLRKNTF

>WP_217327177.1 hypothetical protein [Prevotella copri]

MMKWYQKRDFSIKVPLSFQMKLGKLQQRDASASTLFQEMWDECEDIAKAVLETDYFKGILNNNLDPNAYGSLMVQDAYYCFKAQNSYTIARTHAYDSDCSEFLAGKE
QSYKDYNETYHKIWHIREDYGVIPGPEIKEYADYEAYVASQLDTPYLFAVMLPCEYLWNWVANQLAPKASKDGLYYFWIDGNAGSPTGALQMESMLELYRADIDENKA
KEIFRTAMEFELKVFTSATILNLSYYGKKEI

>WP_234367671.1 hypothetical protein [Parabacteroides pacaensis]

MDDACGDFLQGKYTSYEEYNAYYHETWHVREASGVIPGDEIKEYAAYEAFVAGNLDSPYLFSVMLPCEYLWNWIANELDKTAPKDGLYYFWIEGNGGTPNGAYQMAN
MLESYRGQIDEAKAKEIFRIALQHELKVFTAATLLKSELLWQRKNSSLQR

>MBV9108948.1 TenA family transcriptional regulator [Gemmatimonadetes bacterium]

MRSPRRIRVTPEMAAAHDLSTDPPPPDALFWTLWNACSQIAQQALETPYIQGIRAGTLDPTHYGAFNVSDAYYCFRGADDYKAVVERTADPRLRAFLKSKYDSYLNYNA
TFPKQWRVRDPASIVPFDICHDYCEFESAVAANDDPIYTLVAMLPCEYLWPWLASQLPQPAPTNLYASWVADNLGFGGAYAIGNFLESYRTRHPGSVDPAKATEIYTQA
MNFEHQNFTAATS

>OJJ13921.1 TenA family transcriptional regulator [marine bacterium AO1-C]

MKNNRSITIENDFLSTHQLSKASPAANSLFWKMWNACKNIAEEALKTDFIQGIKTATLDPVKYGGFNVSDAYYCFKGAQDYLSAANRASHPTLAAFLLKKHNSYLTYNQ
TFPTTWHVKDATGIVPTTVCEQYSDFETQVAANEAPIYSLITMLPCEYLWAWLGSELSPPTEGNLYADWITGNDYPDGAYTMGNFIENYRKENPIDESKALQLYTQAMTY
EYENFKVATTQS

>WP_166782516.1 MULTISPECIES: hypothetical protein [Acinetobacter]

MTNISFFPRSFNITQEEIEKLQFSTTLSVEPPPKNSLFQIMWDECLPIAEKVLATDYFQHMIIGNLDPQDYGRLINQDSFYCFNGQFDYELASQRAQSDKILFDFYNAKAQSY
KRYNVYFTNYWGIKDRNSIFPNNFINAYVEHEKLIASNEQSPYLMCAMLPCEYLWFCIARGIDNRVKLSNIYRFWVEENLSGGGQGAYLMGNILEKYRNQLDINKCIRIY
MESMKHELNVFTNATLKSEFMHIK

>HAA12424.1 TenA family transcriptional regulator [Cytophagales bacterium]

MKSNRRITLSSETIAKHELSTEPPAPDSLFWKLWKANLQVAEEALNTDFVQGIKAGTLSPTTYGGFNVSDAYYCFRGAEDYATAVSRAEHPVLKAFLQQKHDSYQSYNET
FPDTWHVKDGDSIVPTEVCQQYSAYETQVVSTEEPIYALVVMLPCEYLWAWLGAQLSPPAAGNVYADWITGNDYPSGAYAMGNFLEAWRQKYGIDEAKAMDIYTKAI
TFEYQNFANP

>MCG8329915.1 hypothetical protein [Chitinophagales bacterium]

MKNNRSIQLDPQFIKEHQVSKASPEAGSLFWKLWQSCTDIADEVIKTPFIQGIKEGTLDPVVYGGFNVSDAYYCFHGASDYLGAANRATDPTLKAFLMKKYNSYDSYNKT
FPKKWHIKDANGVVPTDACKEYSAFESNTAATEDPIYSLIVMLPCEYLWAWLGKELSPPSKGNLYAPWITGNNYPDGAYAMGNFISYFQNKYPGSLDTDKAMELYRAA
MNYELENFKSATE

>WP_198135260.1 hypothetical protein [Aeromonas rivuli]

MLDQHGLSSAPPPTDSLFWRLWQPCIPIAEKTLNTPFMSGIKSASLDPVTYGGFNVSDAYYCFNGAQSYLAAESRTSDEVLKFFLLEKYKRYQAYNESFPTIWHIRDANGV
VPTTACRDYAQFESDTASHADPIYTLIMMIPCEYLWYWLANQLMPPSPGNLYAPWITGNNSASGAYAMGNFLDQYQKEHPGAIDEDKAMSIYSTAMNFEWKNFLSATQ
P

>WP_066308905.1 TenA family transcriptional regulator [Aquimarina aggregata]

MKNNRRIELDKSFLVEYKLSTAPPPANSLFWKMWDDCMQIADDALNTDFIQGIADGTLDPVTYGGFNVSDAYYCFNGEQDYLTAAEKATNPGLQAFLLKKHKSYQKYN
KTFPDIWHIKDANGIVPSKACKEYAEYESNVVSQMESIYALIVMIPCEYLWAWLGNQLTPPETGNLYAPWITGNDDFEGAYAMGNFLNEYQKKHVINESIAIDIYTKAMT
YEYLNFMTATKTKFNTQLL

>WP_224431244.1 hypothetical protein [Aeromonas rivuli]

MLDQHGLSSAPPPTDSLFWRLWQPCIPIAEKTLNTPFMSGIKSASLDPVTYGGFNVSDAYYCFNGAQSYLAAESRTSDEVLKFFLLEKYKRYQAYNEAFPTIWHIRDANGV
VPTTACRDYAQFESDTASHADPIYTLIMMIPCEYLWYWLANQLMPPSPGNLYAPWITGNNSASGAYAMGNFLDQYQKEHPGAIDEDKAMSIYSTAMNFEWKNFLSATQ
P

>WP_118501722.1 TenA family transcriptional regulator [Aquimarina sp. AD10]

MKNNRRIELDKSFLVEYKLSTAPPPANSLFWKMWDDCMQIADDALNTDFIQGLADGTLDPVTYGGFNVSDAYYCFNGEQDYLTAAEKATNPGLQAFLLKKHKSYQKY
NKTFPDIWHIKDANGIVPSKACKEYAEYESNVVSQMESIYALIVMIPCEYLWAWLGNQLTPPETGNLYAPWITGNDDFEGAYTMGNFLNEYQKKHVINESIAIDIYTKAM
TYEYLNFMTATKTKFNTQLL

>MBL7710767.1 hypothetical protein [Chitinophagaceae bacterium]

MNNRSIQMTPEFLRQYGLSADPPPSNSLFFQLWNANIAVATSALNTAFIQGIKNGNLDPVKYGAFNVTDAYYCYEGAEDYLVAESQATDPVLRAFLLKKYNSYLKYNQT
FTSIWHLRDASGVVPVPVVQAYAQFEEQVASHQLPIYMLITMIPCEFLWYWLGNQLSPAAPGNLYAPWINGNNDPSGAYAMGNFLQTYQQQNPVDVNLALQLYTQAM
NFELQNFQAATQ

>WP_012499220.1 TenA family transcriptional regulator [Chloroherpeton thalassium]

MKNPRRLVLTAEFIAKHGLSTAPPPENSLFWKMWNACESIAQASLNTDFIQGIKNGTLDPVKYGAFNVSDAYYCFNGAQDYLAAESRASDPTLRAFLYEKYNSYQTYNE
EFPDTWHIRDARGVIPLEICEEYSGFEKTVASHENPIYTLVAMIPCEYLWYWLAHELYPPASGNLYAPWIDGNNYPDGAYAMGNFLNDYQIAHPDEIDENTAIEIYTQAMT
YEQRNFSAATASQS

>MCG8574180.1 hypothetical protein [Flavobacteriales bacterium]

MKNPRSIRLEDDFIAQHQLSTSPPPPNSLFWKMWNASENIAQQALNTDFIQGIKNGTLNPVTYGAFNISDAYYCFHGAADYLTAANKATHSVLKAFLLKKHKSYQQYNDT
FPKTWRIKDANGIVPTEVCQQYSQFESGTASHEHPIYMLVVMLPCEYLWAWLAAQLSPPTQGNLYASWIKGNDDPKGAYAMGNFLNDYAKENPLDEDLALKLYIQAMT
YEFRNFKTAVGS

>WP_218329357.1 hypothetical protein [Aeromonas sp. sif2433]

MFSPRRLQFDNMLLERHGLSSAPPPADSLFWRLWQPCLPIAQKTLVTPYIDGIKNANLDPVIYGGFNVSDAYYCFNGAQSYLAAESRTDDEGLKFFLLEKYKRYQTYNEA
FPTIWHVRDASGVVPTPACRDYAQFESDTASHADPIYTLIMMIPCEYLWYWLANQLMPPSPGNLYGPWITANDSTSGAYAMGNFLDQYQRAHPGAIDENKAQNIYSTAM
NFEWQNFLSATQS

>WP_242378554.1 hypothetical protein [Aeromonas encheleia]

MATRSCNDTGYKEDNMFSPRRLQFDNMLLERHGLSSAPPPADSLFWRLWQPCLPIAQKTLVTPYINGIKNANLDPVIYGGFNVSDAYYCFNGAQSYLAAESRTDDEGLK
FFLLEKYKRYQTYNEAFPTIWHVRDASGVVPTPACRDYAQFESDTASHADPIYTLIMMIPCEYLWYWLANQLMPPSPGNLYGPWITGNDSTSGAYAMGNFLDQYQRAHP
GAIDENKAQSIYSTAMNFEWQNFLSATQS

>WP_218389057.1 hypothetical protein [Aeromonas sp. sia0103]

MATRSCNNTGYKENNMFSPRRLQFDNMLLERHGLSSAPPPVDSLFWRLWQPCLPIAQKTLVTPYINGIKNANLDPVIYGGFNVSDAYYCFNGAQSYLAAESRTDDEGLK
FFLLEKYKRYQTYNEAFPTIWHVRDASGVVPTPACRDYAQFESDTASHADPIYTLIMMIPCEYLWYWLANQLMPPSPGNLYGPWITGNDSTSGAYAMGNFLDQYQRAHP
GAIDENKAQNIYSTAMNFEWQNFLSATQS

>WP_218291627.1 hypothetical protein [Aeromonas sp. sif2416]

MATRSCNDTGYKENNMFSPRRLQFDNMLLERHGLSSAPPPVDSLFWRLWQPCLPIAQKTLVTPYINGIKNANLDPVIYGGFNVSDAYYCFNGAQSYLAAESRTDDEGLK
FFLLEKYKRYQTYNEAFPTIWHVRDASGVVPTPACRDYAQFESDTASHADPIYTLIMMIPCEYLWYWLANQLMPPSPGNLYGPWITGNDSTSGAYAMGNFLDQYQRAHP
GAIDENKAQNIYSTAMNFEWQNFLSATQS

>WP_149626228.1 TenA family transcriptional regulator [Aquimarina sp. RZ0]

MKNNRSTKLDNSFLAEHKLSTAPPPLDSLFWKMWNACQSIANQALNTEFIQGIKTGTLDPVSYGGFNVIDAYYCFNGEQDYLVAESKASNPILKAFLLKKYNSYQKYNET
FPKVWHVRDAGGIVPSKVCKQYSEFESNVASHKAPIYTLIAMMPCEYLWAWLGAQLSPPLSGNLYAPWITENDYPDGAYAMGNFLVEYQKENPVDETLATQLYSQAMT
YEFQNFNTAINR

>WP_197720907.1 hypothetical protein [Aeromonas encheleia]

MATRSCNDTGYKENNMFSPRRLQFDNMLLERHGLSSAPPPVDSLFWRLWQPCLPIAQKTLVTPYIDGIKNANLDPVIYGGFNVSDAYYCFNGAQSYLAAESRTDDEGLK
FFLLEKYKRYQTYNEAFPTIWHVRDASGVVPTPACRDYAQFESDTASHADPIYTLIMMIPCEYLWYWLANQLMPPSPGNLYGPWITGNDSTSGAYAMGNFLDQYQRAHP
GAIDENKAQSIYSTAMNFEWQNFLSATQS

>WP_133537109.1 TenA family transcriptional regulator [Tenacibaculum caenipelagi]

MKNNRSTKLDQTFLTEYKLSTAPPPTDTLFWKMWNACQDIANETLNTQFIQEIKSGTLNPVHYGGFNISDAYYCFKGAQDYLNAMDRASNPTLKAFLHKKYNSYQKYN
DTFPKTWRIKDAEGIIPSEVCKLYSKFESMVASHQDPIYALIVMIPCEYLWAWLAAQLSPTSSKNLYAPWITGNNDPNGAYAMGNFLEEYQKENPVDEKLATQLYTQAIT
YEYQNFNTALSS

>WP_105712671.1 hypothetical protein [Acinetobacter sp. MYb10]

MWDECLPIAEKVLATDYFQHMIIGNLDPQDYGRLINQDSFYCFNGQFDYELASQRAQSDKILFDFYNAKAQSYKRYNVYFTNYWGIKDRNSIFPNNFINAYVEHEKLIAS
NEQSPYLMCAMLPCEYLWFCIARGIDNRVKLSNIYRFWVEENLSGGGQGAYLMGNILEKYRNQLDINKCIRIYMESMKHELNVFTNASLKSEFMHIKQPFQIKSLKLQFN
QRVTQKHKKL

>WP_012909371.1 TenA family transcriptional regulator [Pirellula staleyi]

MKSPRRIVLTDEIIARHNLCTDPPPSDSLFWLLWNTCIQTAQQALQTPFIQGVKAGTLDPVVYGGFNVNDAYYCFNGAPDYQSAAQRASDVTLQQFLTAKYNSYQTYNA
TFPQIWRVKDATSIIPFDVCQQYSQFESYIATKFDPIYCLIAMLPCECLWAWLGEQLSPPDPQNLYAPWVNGNNDFSGAYAIGNFLQSYQIANPGVVDQSLSLKIYSQAMIY
EQQNFAAALSATD

>WP_121897937.1 TenA family transcriptional regulator [Rhodophyticola porphyridii]

MRGNRRLSDISDIIEKHQLSTAPPPADSLFWKMWNGVQDVATDTLALPYLQGINAGTLDPNQYGGYNVADAYYCFEGADAYQTAVSKTTPNTPLNAFLSKKLAGYQRY
NATFPDTWHVSSAASVTPPQVTKDYAEYEGNVAAQLDPIYCLIVMIPCEYLWAWLAGQMNPPQPTNVYGEWITENNDPSGAYAMGNFLDSYMTQNPGVVDPPYAQLIY
GFAQIYEYQNFAAATGTQPNDAAHYGIGKPAIPPS

>WP_125344594.1 TenA family transcriptional regulator [Tenacibaculum litoreum]

MRNNRATLLDQTFLTEHKLSTAPPPADSLFWNMWNACQEIADKALNTQFIQEIKSGTLNPVDYGGFNINDAYYCFNGAQDYLTAMNRASNPTLKALLFKKYHSYQKYN
ETFPKTWHIKDAKGIVPNEVCKQYSQFESMVVSHQDPIYALIVMIPCEYLWAWLAEQLAPPSSKNLYASWITANNDPGGAYAMGDFLSDYQKEKPIDEKLATQLYTEAIT
YEYQNFNTALNS

>MCE7991650.1 TenA family transcriptional regulator [Roseivirga sp.]

MKNNRAIQLPQETINLHNLSTAPPPENSLFWKMWHTCEDIAQGALATKFVQGIGTGTLHPQTYGAFNVSDAYYCFNGAADYQTAVDKATNPVLKAFLQHKHDSYESYN
NTFPDTWRVKNGDSIVPTAVCKAYSDFESNICANEEAIYALVVMLPCEYLWIWLGQQLAPAKEGNLYSDWITGNQDPSGAFAMGNFINAYQRANPIDEDKALAIYKQAIT
YERDNFATA

>NER24802.1 TenA family transcriptional regulator [Symploca sp. SIO1C2]

MKNNRSLADHEHLIAKHNLNRNPPPPDSLFWKMWYAGGESIAKKTLTTGFLEGIKNGNLDPNQYGPFSVSDIYYCFHGAADYGIAAKRATNTVLQDYFLKKQESYNTYN
QSACATWNLTGPESIAPTPTALNYSNFERSVAKGTAQEGNVKDPIFTLIVMLPCEYLWPWLASQLAPPTPGNIYASWITSNNNPDGAYAMGNFLQDYITLNPINEDVATTT
YLTAMEYEYKNFYTATPELS

>NCQ92070.1 TenA family transcriptional regulator [Microcystis aeruginosa LG13-13]

MRNNRSLIDHDYLIKKHKLNTNPPPPNSLFWKMWNAGGEWIAQEALNTGFLKGIKNGNLNPTHYGAFNVSDVYYCFNGASDYGIAAQRTTNIILQDYLLKKQQSYNQY
NQSACITWNLASPQSIQPTPTALNYSLFEQSVANGSAKEGNVKDPIFTLIVMLPCEYLWAWLASQLAPPAPSNIYASWITSNNQPDGAYAMGNFLQEYITLNRIDENLATVI
YLTAMEYEYSNFSSATQPG

>REJ51290.1 TenA family transcriptional regulator [Microcystis wesenbergii TW10]

MRNNRSLIDHDYLIKKHKLNTNPPPPNSLFWKMWNAGGEWIAQEALNTGFLKGIKNGNLNPTHYGAFNVSDVYYCFNGASDYGIAAQRTTNIILQDYLLKKQQSYNQY
NQSACITWNLAGPQSIQPTPTALNYSLFEQSVANGSAKEGNVKDPIFTLIVMLPCEYLWAWLASQLAPPAPSNIYASWITSNNQPDGAYAMGNFLQEYITLNRIDENLATVI
YLTAMEYEYSNFSSATQPG

>NEO58885.1 TenA family transcriptional regulator [Okeania sp. SIO3B5]

MKNNRSLGDHEHLITKHKLNRNPPPPDSLFWKMWYAVGEPIAQKTLTTGFLEGIKNGNLDPNQYGAFSVSDIYYCFHGASDYGIAAKRTTNTVLQDYYLKKQDSYNRY
NQSACTTWNLTGPESIAPTPTALDYSNFERSVANGTAQEGNVKDPIFTLIVMLPCEYLWAWLASQLAPPKPGNIYASWITSNNYPDGAYAMGNFLQDYITLNPINEDVATT
IYQTATEYEYKNFYTATPEPT

>WP_236115769.1 hypothetical protein [Maritalea sp. P4.10X]

MRNNRSIGDVSQIAKDFSLSTAPPPANSLFWEMWNQNIVQENAQSTLNLPYLQGINSGLLDPNDYGGYNVADAYYCFNGADDYQAAAAKAPSGSALQAFLNKKLAGYQ
RYNATFPSTWHVASAADVTPPEITKAYSDFERKVVAQYDPVYAIIVMLPCEYLWAWLASQMNSPSSQNVYGGWITGNNDPSGAYAMGNFLVQYQAANPGVIDQTLAN
NIYAWANFYEYANFSAATNTQIADPDILGLTPLPDLVSA

>WP_207380639.1 hypothetical protein [Shewanella sedimentimangrovi]

MRNPRRLHDHRLLVEKHRLDTQAPAADSLFWRLWQDGGSELAHKALATGFVQGIRTGSLDPRSYGAFNVSDIYYCCKGADDFGDAALRTRDPVLRDFLLHKQQSYEKY
YRAACREWRLADPKAVLPSDTAKRYSDFERRLATGQAEAGKAADPIYTLIAMLPCEYLWGWLAQQLSPATDTNLYGPWISSNACVAGAFAIGNFIEAYIGEHAVDEELA
RQLYLTAMELEYRNFEGALQ

>ETW93821.1 hypothetical protein ETSY1_37535 [Candidatus Entotheonella factor]

MAAHLWNASQDLAQAALESDYIQGIKQGTLNPNNFGQYSVQDVAYCHNGLEDWKAVATRAQQPDIKAFAEARVKSWAKYAQETYGAWHISDPAAVQLSVAAQTYSA
FESEVAHHYDPLYTVVVMIPCDRLWYWLANQIKSGSSATNVYDFWINGNSASDNGAHQMENVVDTHAAQLDEDTALHVYRTAMLGEVNFFRSACRQPLLEMPGTS

>PXF48067.1 hypothetical protein BWQ96_02019 [Gracilariopsis chorda]

MNSARSCFHSGNPIITLKHALNCPRTVSVTFSIAEKHNLSKTSPPEHSLFWRLWHQNKHYAKKALATTFIQDMVKGTLNPISYTRYNVSDAYFCYSGADDYKTAMNKAQ
DAVLKAYLSAKYRSYKRYNEMFPRTMCLKDASGIEPNAVCKEYSDFERSVVRDEEAVYTIFAMLPCEFLWPWLGSQIEYPDVTNVYREWIMMNLKVEQGNAFAAGNFL
VDFESVYGREIDHDKAGRIYRQAMVYEWQNFATACSY

>WP_207321561.1 hypothetical protein [Shewanella cyperi]

MRNPRRLHDHRLLIEKHQLDTQSPAADSLFWRLWQDGGSELAHKALATGFVQGIRTGSLDPRSYGAFSVSDIYYCCQGANDFGDAARRSRDPVLQDFLLHKQQSYDKY
FREACNEWRLADPKAVLPSDTAKRYSDFERRLATGQAEAGKAADPIYTLIAMLPCEYLWGWLAQQLSPASDTNLYGPWISSNACVAGAFAMGNFIEAYLGEHVVDEEL
ARHLYLTAMEHEYRNFEGALP

>WP_051214019.1 hypothetical protein [Maritalea myrionectae]

MQNNRSIGDITQLAKDFSLSTAPPPANSLFWEMWNQNIVQQNAKATLNLSYLQGINSGLLDPNVYGGYNVADAYYCYNGADDYKIAANAAPSGSALEAFLLKKYDGYQ
KYNATFPGTWHVASAADVTPPEITKAYSQFEHNVVTQYDPVYAIIVMLPCEYLWAWLASQMNTPSPQNVYGGWITGNNDPSGSFAMGNFLVEYMAANPGKIDQKLAN
NIYAWANFYEYANFSAATNAEIVDPASLGLTPLPDFVPS

>WP_117394815.1 TenA family transcriptional regulator [Maritalea myrionectae]

MQNNRSIGDITQLAKDFSLSTAPPPANSLFWEMWNQNIVQQNAKATLNLSYLQGINSGLLDPNVYGGYNVADAYYCYNGADDYKIAANAAPSGSALEAFLLKKYDGYQ
KYNATFPGTWHVASAADVTPPEITKAYSQFEHNVVTQYDPVYAIIVMLPCEYLWAWLASQMNTPSPQNVYGGWITGNNDPSGSFAMGNFLVDYMAANPGKIDQKLAN
NIYAWANFYEYANFSAATNAEIVDPASLGLTPLPDFDPS

>NQY95417.1 TenA family transcriptional regulator [Campylobacteraceae bacterium]

MKNPRKIVLTDEMIKKHKLSTQPPPQNSLFWAMWNASEEIAQEALGTEFINGIKNGTLDPVTYGGFNVSDAYYCFNGADDYKTAHDKATDPVLKFFLEQKYISYEKYNL
DFPTTWRIKDASGVVPTDACKEYSEFESSIAGGINSIYTVVAMLPCEFLWYWLAS

>XP_005710530.1 unnamed protein product [Chondrus crispus]

MTLTATLAPPRLARTMRNPRALTETPELLLQLLGPRTPPRVDSLFWTLWSKSADVACKARRTPFIQGIAAGTLDPVVFGRYHVSDAYYSFHAADDYLLAAARATDPVIKA
FLAAKHASYTAYNRTFPALWHIKDCNGIDPGQVCKAYSSFERSIVAAEDAIYTLIVMLPCEHLWPWIGTSIGPPDKANLYARWINHNSDFGSAFALGNVIDNFELRNPRILC
RDKALYIYRKAMVYEWKNFENA

>WP_207325153.1 hypothetical protein [Shewanella cyperi]

MRNPRRLHDHKLLVEKHRLDTQAPAADSLFWRLWQGGGSELAQKALATGFVQGIRTGSLDPRSYGAFNVSDIYYCCQGANDFGDAARRSRDPVLQDFLLHKQQSYEK
YYRVACLEWRLADPKAVLPSDTAKRYSDFERRLAKGQMEAGKAADPIYTLIAMLPCEYLWGWLAQQLSPASDTNLYGPWISSNACVAGAFAMGNFIEAYLGNHDLDE
GLARHLYLTAMEHEYRNFEGALQQP

>MBO6624016.1 hypothetical protein [Roseicyclus sp.]

MQDVATDTLALPYLQGINAGTLDPNQYGGYNVADAYYCFEGADAYQTAVSKTTPNTPLNAFLSKKLAGYQRYNATFPDTWHVSSAASISPPQVTKDYAEYEGNVAARL
DPIYCLIVMIPCEYLWAWLAGQMNPPQPSNVYGEWITENNDPSGAYAMGNFLDSYMTQNPGVLDTSYAQLIYGFAQIYEYQNFAAATGTQPNDAAHYGIDKPAIQPS

>MBO6603666.1 hypothetical protein [Roseicyclus sp.]

MATDTLALPYLQGINAGTLDPNQYGGYNVADAYYCFEGADAYQTAVSKTTPNTPLNAFLSKKLAGYQRYNATFPDTWHVSSAASISPPQVTKDYAEYEGNVAARLDPI
YCLIVMIPCEYLWAWLAGQMNPPQPSNVYGEWITENNDPSGAYAMGNFLDSYMTQNPGVLDTSYAQLIYGFAQIYEYQNFAAATGTQPNDAAHYGIDKPAIQPS

>WP_208111211.1 hypothetical protein [Maritalea mobilis]

MRNNRSIGDIAELAKDFSLSTAPPPANSLFWEMWNQNIVQQNAQATLNLSYLQGINSGLLDPNVYGGYNVADAYYCYNGADDYQYAAGQAPAGSALQAFLLKKFAGY
QKYNATFPDTWHVASAADISPPEITKAYSQFEHNVVTKYDPIYAIIVMLPCEYLWAWLASQMNTPTSQNVYGGWITGNNDPSGAYAMGNFLVEYMAANPGQIDQKLAN
NIYAWANFFEYANFSAATNTAFADPASLGLTPLPEFEPAN

>MBR1693707.1 hypothetical protein [Lachnospiraceae bacterium]

MNRIYTRNIRFTARLQKGAQVRTAKPPEDSFFFRVFEKNLDVANAVIDTKYLQAMKHGVLNPQDYGCLTVLDSYYCYRAADTLLSLLSEIDDKEYPDLKRLVEVQYEGY
EEYNSTFFRDWHIRTADSVNPTDTMMAYAEHEHHVMCAYPPIYTLVAMLPCYYLWPWFSQKIEESSDYEPGVYKDWFEGNYSGEDSYDSAYEIGNFIDAWQKDGKEFD
ESLAGEIFSKSMNYELAAFTEAYHEAIEGKGGKSCG

>WP_002614061.1 TenA family transcriptional regulator [Stigmatella aurantiaca]

MTSRLILDTPRKSSAQLSTANRLDTPAPQRGESLSKELWARTQDVAQEALGSTFIQGIKHGSLDPNSFGQYTIQDAVYCYQAQRDYEVLASRITRPDLKAFVEARRDGYAK
YNQQTFALWHIREPNALSLSPAAKAYSDFESMVAANDEPLYAIVAMIPCERLWSWLANQMIGDAGPGNLYSFWITGNTSDTGACRLEAFVDAHAHHLDEGRALSVYRT
CMLGECNFFRSACKQDPLLLEPARITATA

>XP_022093498.1 uncharacterized protein LOC110980809 [Acanthaster planci]

MSVWQKGLKNIRSPRSTAAMLEHLPQTVIDRLAAKHPAKLTKVPVADGRYEEKELSEWLWESSQVQAQLALHTKFIQGIKSGLLDPTDYGGYTVQDAVYCSNATTYYGI
AEDKSQDEIMKAFIQSRVKSYTSYTKVMFKQWHIRDPSGVAMGAAAASYSAFEKGVAENCEPIYLLIAMLPCEKLWEWLAEQIQSGISETNVYSFWITDNLGGSSKLAHFI
DAYADEYGVDLQKAKDIYHGAMQGEVNFFASATAEVP

>XP_038050170.1 uncharacterized protein LOC119723538 [Patiria miniata]

MSSLRKKSIKNVRSPRRIAACVEHLPQSFVDQLLAPKRKAKLMKVADTAEGGERELAEWLWESSQAQAQEALDTGFIQGIKSGLLDPTDYGGYTVQDAVYCFNATSYYG
IAYEKCKESTLREFIQGRIKSYAGYTEEMFKQWYIKDPRGIAMGTAAASYSSFEKGVATNSQAIYLLIAMLPCEQLWGWLAEQIQPGINDTNVYSFWIEDNLGGSHKLANF
INEHTESYGVRPEVAKGIYKTAMQCEVEFFRSATIDVV

>XP_003730491.2 uncharacterized protein LOC100893025 [Strongylocentrotus purpuratus]

MSALKRSPRNVRSPRSTAAMVKHLPSAVAALLQSGKGPAKLTNVDSPDGVKAMPLADWLWDKSQVQAQAALDTDFIQGIKSGLLDPTNYGGYTVQDAVYCHNATDY
YGIAECRATDEDLKEFIAARVKSYASYTEIMFKEWYIQDPKGIAMGDAAASYSKFELDVATKEEPFYLLIAMLPCEKLWEWLSQQIESGINDTNVYSFWIQDNLGGSHKL
ATYVNENAEKFGVDLPKAMDIYQKGMQCEVDFFTSGTIEEVN

>XP_030844619.1 uncharacterized protein LOC115918738 [Strongylocentrotus purpuratus]

MSALKRSPRNVRSPRSTAAMVKHLPSAVAALLQPGKGPAKLTKFDTPGGEKWMTLTEFLWNISQVQAQEALNTDFIQGIKSGLLDPTNYGGYTVQDAVYCDNATGCYE
KAESKATDEDLKKFIAARIESYAEYTEIMFKEWYIKHPKGIAMGDAAASYSEFELDVATTEEPFYLLIAMLPCEKLWGWLAQEIKSGINDTNVYSFWIQDNLPGSHTLANY
IDENAEKFKVDLKKAMKIYKDGMQCEVDFFTSGTIEEVN

>XP_030844031.1 uncharacterized protein LOC100892956 [Strongylocentrotus purpuratus]

MSALKRSPRNVRSPRSTAAMVKHLPSTVAALLQPGKGPAKLTKFDTPGGEKWMTLTEFLWNISQVQAQEALNTDFIQGIKSGLLDPTNYGGYTVQDAVYCDNATGCYE
KAESKATDEDLKKFIAARIESYAEYTEIMFKEWYIKHPKGISMGDAAASYSEFELDVATTEEPFYLLIAMLPCEKLWGWLAQEIKSGINDTNVYSFWIQDNLPGSHTLANYI
DENAEKFKVDLKKAMKIYKDGMQCEVDFFTSGTIEEVN

>XP_030844616.1 uncharacterized protein LOC115918737 [Strongylocentrotus purpuratus]

MLLSCGCTNRPSRLNSPKDWSIILNMSALKRSPRNVRSPRSTAAMVKHLPSAVAALLQSGKGPAKLTKVDTPDGVKAVPLADWLWDKSQVQAQAALDTDFIQGIKSGLL
DPTNYGGYTVQDAVYCDNATDYFGIAEMKACDKDLKEFIAARIKSYAEYTEIMFKEWYIQDPKGIAMGDAAASYSKFELGVATKEEPFYLLIAMLPCEKLWGWLSQQIE
SGINDTNVYSFWIQDNLPGSHTLANYIDENAEKFKVDLKKAMKIYKDGMQCEVDFFTSGTIEEVN

>XP_030844615.1 uncharacterized protein LOC115925196 [Strongylocentrotus purpuratus]

MLLSCGCPNRPSRLNSPKDWSIILNMSALKRSPRNVRSPRSTAAMVKHLPSAVAALLQSGKGPAKLTKVDTPDGVKAVPLADWLWDKSQVQAQAALDTDFIQGIKSGLL
DPTNYGGYTVQDAVYCDNATDYFGIAEMKACDKDLKEFIAARIKSYAEYTEIMFKEWYIQDPKGIAMGDAAASYSKFELGVATKEEPFYLLIAMLPCEKLWGWLSQQIE
SGINDTNVYSFWIEDNLPGSHTLATYINENAVRFGVDPQKAMDIYQQGMQCEVDFFTSGTIEEVN

>XP_022093516.1 uncharacterized protein LOC110980819 [Acanthaster planci]

MSLRQKSVKNVRSPRSASGAAFVLQHLPQTVTELLAAKRAAKKPTEVTVVDGRPQEWKELSEWLWDGSQVQAKEALNTEFIQGIKSGLLDPTDYGGYTVQDAVYCYN
ATTYYLTAAGKTSDLAMSKFIQGRIDSYKSYTQTMFDEWHIRDPTGVSMGTAAASYSAYEKHVAETNDPIYLLIAMLPCDQLWGWLAEQIKSGISDKNVYSFWIEDNLPQ
HKSKLAEFIDANAHYYEVFWPEAKVIYQRAMQGEVDFFTSATPPPKNA

>HAS42904.1 TenA family transcriptional regulator [Microscillaceae bacterium]

GAQDYLAAANRASHPTLKAFLLKKHNSYQTYNDTFPTTWHVKDASGIVPTEMCKQYSAFETEIATNEEPIYTLITMLPCEYLWAWLGSELSPPSNGNLYADWITGNDYPD
GAYTMGNFIESYRQQYPIDEPKALKLYTQAMTYEYQNFKVATE

>WP_078745630.1 hypothetical protein [Oceanospirillum multiglobuliferum]

MFNSLLSRSPRSKTPLKSINLRPQLQTDASPSSLYDQMWAATTLYQQTALHSDFIQGIAHCTLDPDQYGQFTVLDAAYCSYSVADYDIVIPKAQNPEIKSFCEARQKDYEN
YNAEVFAGWHIKDPAGIKLTSIAQQYASFERYVAEELDPLYFIVAMTPCISLWTYIGVSLNHYATPQNLYSSWIKANQVVDAPKSMADFINAHANEMNQQLAIDIFSKCM
QFECNMFLSACNQPPV

>WP_038801483.1 MULTISPECIES: hypothetical protein [pseudomallei group]

MKPLVTRTPRRIRLDHPSLAKLDVSTSPPLADSITSQLWEACYSLAQDALNSPYIQGIANGTLPPCNYGQYTVQDAAYCVRAEQDYRLVEARAKEHHEDMLAAFAQARY
ESYLSYTDSIMKSWHIKDILAINPNDAVKAYVAHEHYVAEFMEPIYGVVAMIPCDHLWSWLAETLSPDNVPNNLYDFWISDNQGWSGTYRLENFVNSWFAAHPKQYEW
ESALRAYKGSMLGEVGDFRAALE

>XP_021377140.1 uncharacterized protein LOC110465552 [Mizuhopecten yessoensis]

MALRNPRLSLHVHDIAKRKVVKTDLEPLSQYLWEQTESTRQKALATYFVQGVGKGNLNPTAFGGFMVQDSVYCYKAKGSIDVAASRAQEGDLKAYLQKESGSYESYY
QDLFTKWHIKDATGIDLDPACQSYAEYEHLVASTKDTIYMIVAMIPCMKLWPWLGQQLQTFNHGVYTDWYNANFDPTYDGYKQLDEFVDTAGTTIDKETALKVYSRC
MEGEYEFFNSAQLMM

>MBR3314682.1 hypothetical protein [Atopobiaceae bacterium]

MHHLISRNISLLMYLPEALRRLVGKPLPTSFYSRAFFANLLTANAVAATRYLQSMHDGTLNPLDYGSLTIQDAYYCYHAQDTLKELLDRIDREKSPDLYDLVASKASAYD
NYNRTFLEDWHIRDAECVLPTDGTRAYVAHEQRVAREEEPIYSLVAFLPCYNLWPWFARQLMASPRYNPGIYRDWFEGVYEGEVESFGGAWALGSFIEEWKDGGGAFD
ESLAHDIYRTSMEFELRMFREACTEE

>WP_232516833.1 hypothetical protein [Burkholderia pseudomallei]

MKPLVTRTPRRIRLDHPSLAKLDVPTSPPPANSITAQLWGACIGLAQDALNSPYIQGIANGTLPPCNYGQYTVQDAAYCVRAEQDYRVVEARAKEHHEDVLAAFALARYE
GYQSYTDSIMESWHIKDILAIDPNDAVKAYVAHEHYVAEFMEPIYGVVAMIPCDHLWSWLAETLSPDNVPTNLYDFWINENQGWTGTYQLENFVNGWFAKHPKQYEW
ESALRAYRGSMLGEVGDFRAALE

>WP_038722475.1 hypothetical protein [Burkholderia pseudomallei]

MMKPLVTRTPRRIRLDHPSLAKLDVPTSPPPANSITAQLWGACIGLAQDALNSPYIQGIANGTLPPCNYGQYTVQDAAYCVRAEQDYRVVEARAKEHHEDVLAAFALAR
YEGYQSYTDSIMESWHIKDILAIDPNDAVKAYVAHEHYVAEFMEPIYGVVAMIPCDHLWSWLAETLSPDNVPTNLYDFWINENQGWTGTYQLENFVNGWFAKHPKQYE
WESALRAYRGSMLGEVGDFRAALE

>WP_023359741.1 TENA/THI-4/PQQC family protein [Burkholderia pseudomallei]

MKPLVTRTPRRIQLDHPSLAKLDVPTSPPPANSITSQLWEACYSLAQDALNSPYIQGIANGTLPPCNYGQYTVQDAAYCVRAEQDYRLVEARAKKHHEDVLAAFAQARY
ESYLSYTDSIMKAWHIKDILAINPNDAVKAYVAHEHYVAEFMEPIYGVVAMIPCDRLWSWLAETLSPDNVPNNLYDFWISDNQGWSGTYRLENFVNSWFAAHPKQYER
ESALKAYRGSMLGEVGDFRAALE

>AIV52139.1 TENA/THI-4/PQQC family protein [Burkholderia pseudomallei MSHR1153]

MKPLVTRTPRRIQLDHPSLAKLDVPTSPPPANSITSQLWEACYSLAQDALNSPYIQGIANGTLPPCNYGQYTVQDAVYCVRAEQDYRLVEARAKKHHEDVLAAFAQARY
ESYLSYTDSIMKAWHIKDILAINPNDAVKAYVAHEHYVAEFMEPIYGVVAMIPCDRLWSWLAETLSPDNVPNNLYDFWISDNQGWSGTYRLENFVNSWFAAHPKQYEW
ESALKAYRGSMLGEVGDFRAALE

>WP_239693774.1 hypothetical protein [Burkholderia pseudomallei]

MKPLVTRTPRRIQLDHPSLAKLDIPTSPPPANSITSQLWEACYSLAQDALNSPYIQGIANGTLPPCNYGQYTVQDAAYCVRAEQDYRLVEARAKKHHEDVLAAFAQARYE
SYLSYTDSIMKAWHIKDILAINPNDAVKAYVAHEHYVAEFMEPIYGVVAMIPCDRLWSWLAETLSPDNVPNNLYDFWISDNQGWSGTYRLENFVNSWFAAHPKQYEWE
SALKAYRGSMLGEVGDFRAALE

>WP_111963042.1 MULTISPECIES: hypothetical protein [Burkholderia]

MAQDALNSPYIQGIANGTLPPCNYGQYTVQDAAYCVRAEQDYRLVEARAKKHHEDVLAAFAQARYESYLSYTDSIMKAWHIKDILAINPNDAVKAYVAHEHYVAEFM
EPIYGVVAMIPCDRLWSWLAETLSPDNVPNNLYDFWISDNQGWSGTYSLENFVNSWFAAHPKQYEWESALKAYRGSMLGEVGDFRAALE

>WP_229656331.1 hypothetical protein [Burkholderia pseudomallei]

MKPLVTRTPRRIQLDHPSLVKLDIPTSPPPANSITSQLWEACYSLAQDALNSPYIQGIANGTLPPCNYGQYTVQDAAYCVRAEQDYRLVEARAKKHHEDVLAAFAQARYE
SYLSYTDSIMKAWHIKDILAINPNDAVKAYVAHEHYVAEFMEPIYGVVAMIPCDRLWSWLAETLSPDNVPNNLYDFWISDNQGWSGTYRLENFVNSWFAAHPKQYEWE
SALKAYRGSMLGEVGDFRAALE

>WP_235512831.1 hypothetical protein [Burkholderia pseudomallei]

MMMKPLVTRTPRRIQLDHPSLVKLDIPTSPPPANSITSQLWEACYSLAQDALNSPYIQGIANGTLPPCNYGQYTVQDAAYCVRAEQDYRLVEARAKKHHEDVLAAFAQA
RYESYLSYTDSIMKAWHIKDILAINPNDAVKAYVAHEHYVAEFMEPIYGVVAMIPCDRLWSWLAETLSPDNVPNNLYDFWISDNQGWSGTYRLENFVNSWFAAHPKQY
EWESALKAYRGSMLGEVGDFRAALE

>WP_229653324.1 hypothetical protein [Burkholderia pseudomallei]

MKPLVTRTPRRIQLDHPSLAKLDVPTSPPPANSITSQLWEACYSLAQDALNSPYIQGIANGTLPPCNYGQYTVQDAAYCVRAEQDYRLVEARAKKHREDVLAAFAQARY
ESYLSYTDSIMKAWHIKDILAINPNDAVKAYVAHEHYVAEFMEPIYGVVAMIPCDRLWSWLAETLSPDNVPNNLYDFWISDNQGWSGTYRLENFVNSWFAAHPKQYEW
ESALKAYRGSMLGEVGDFRAALE

>WP_223854489.1 hypothetical protein [Burkholderia pseudomallei]

MKPLVTRTPRRIQLDHPSLAKLDVPTSPPPANSITSQLWEACYSLAQDALNSPYIQGIANGTLPPCNYGQYTVQDAAYCVRAEQDYRLVEARAKKHHEDVLAAFAQARY
ESYLSYTDSIMKAWHIKDILAINPNDAVKAYVAHEHYVAEFMEPIYGVVAMIPCDRLWSWLAETLSPDNVPNNLYDFWISDNQGWSGTYRLENFVNSWFAAHPKQYEW
ESALKAYRGSMLGEVGDFRAALE

>WP_229274563.1 hypothetical protein [Burkholderia pseudomallei]

MMMKPLVTRTPRRIQLDHPSLAKLDVPTSPPPANSITSQLWEACYSLAQDALNSPYIQGIANGTLPPCNYGQYTVQDAAYCVRAEQDYRLVEARAKKHHEDVLAAFAQA
RYESYLSYTDSIMKAWHIKDILAINPNDAVKAYVAHEHYVAEFMEPIYGVVAMIPCDRLWSWLAETLSPDNVPNNLYDFWISDNQGWSGTYRLENFVNSWFAAHPKQY
EWESALKAYRGSMLGEVGDFRAALE

>ARK69056.1 hypothetical protein BOC38_10045 [Burkholderia pseudomallei]

MMKPLVTRTPRRIQLDHPSLAKLDVPTSPPPANSITSQLWEACYSLAQDALNSPYIQGIANGTLPPCNYGQYTVQDAAYCVRAEQDYRLVEARAKKHHEDVLAAFAQAR
YESYLSYTDSIMKAWHIKDILAINPNDAVKAYVAHEHYVAEFMEPIYGVVAMIPCDRLWSWLAETLSPDNVPNNLYDFWISDNQGWSGTYRLENFVNSWFAAHPKQYE
WESALKAYRGSMLGEVGDFRAALE

>WP_231747168.1 hypothetical protein [Burkholderia sp. BDU5]

MMMKPLVTRTPRRIQLDHPSLAKLDVSTSPPPANSITSRLWEACYSLAQDALNSPYIQGIANGTLPPCNYGQYTVQDAAYCVRAEQDYRLVEARAKEHHEDMLAAFAQA
RYEGYLSYTSSIMKSWHIKDILAINPNDAVKAYVAHEHYVAEFMEPIYGVVAMIPCDHLWSWLAETLSPDNVPNNLYDFWISDNQGWSGTYRLENFVNSWFAAHPKQY
EWESALKAYRGSMLGEVGDFRVALE

>MBR3159736.1 hypothetical protein [Atopobiaceae bacterium]

MHHLISRNISLLMYLPEALRRLVGKPLPTSFYSRAFFANLLTANAVAATRYLQSMHDGTLNPLDYGSLTIQDAYYCYHAQDTLKELLDRIDREKSPDLYDLVASKASAYD
NYNRMFLEDWHIRDAECVLPTDGTRAYVAHEQRVAREEEPIYSLVAFLPCYNLWPWFARQLMASPRYNPGVYRDWFEGVYEGEVESFGGAWALGSFIEEWKDGGGAF
DESLAHDIYRTSMEFELRMFREACTEE

>WP_225316976.1 hypothetical protein [Burkholderia pseudomallei]

MKPLVTRTPRRIQLDHPSLAKLDIPTSPPPANSITSQLWEACYSLAQDALNSPYIQGIANGTLPPCNYGQYTVQDAAYCVRAEQDYRLVEARAKKHHEDVLAAFAQARYE
SYLSYTDSIMKAWHIKDILAINPNDAVKAYVAHEHYVAEFMEPIYGVVAMIPCDRLWSWLAETLSPDNVPNNLYDFWISDNQGWSGTYSLENFVNSWFAAHPKQYEWE
SALKAYRGSMLGEVGDFRAALE

>WP_225315235.1 hypothetical protein [Burkholderia pseudomallei]

MMMKPLVTRTPRRIQLDHPSLAKLDIPTSPPPANSITSQLWEACYSLAQDALNSPYIQGIANGTLPPCNYGQYTVQDAAYCVRAEQDYRLVEARAKKHHEDVLAAFAQA
RYESYLSYTDSIMKAWHIKDILAINPNDAVKAYVAHEHYVAEFMEPIYGVVAMIPCDRLWSWLAETLSPDNVPNNLYDFWISDNQGWSGTYSLENFVNSWFAAHPKQY
EWESALKAYRGSMLGEVGDFRAALE

>EDU08025.1 conserved hypothetical protein [Burkholderia pseudomallei 1655]

MDVPTSPPPANSITSQLWEACYSLAQDALNSPYIQGIANGTLPPCNYGQYTVQDAAYCVRAEQDYRLVEARAKKHHEDVLAAFAQARYESYLSYTDSIMKAWHIKDILAI
NPNDAVKAYVAHEHYVAEFMEPIYGVVAMIPCDRLWSWLAETLSPDNVPNNLYDFWISDNQGWSGTYRLENFVNSWFAAHPKQYEWESALKAYRGSMLGEVGDFRA
ALE

>WP_231752114.1 hypothetical protein [Burkholderia sp. MSMB1498]

MKPLVTRTPRRIQLDHPSLAKLDVSTSPPPANSITSQLWEACYGLAQDALNSPYIQGIANGTLPPCNYGQYTVQDAAYCVRAEQDYRLVEARAKKHHEDMLAAFAQARY
EGYLSYTDSIMKSWHIKDILAINPNDAVKAYVAHEHYVAEFMEPIYGVVAMIPCDHLWSWLAETLSPDNVPNNLYDFWISDNQGWSGTYRLENFVNSWFAAHPKQYEW
KSALKAYRGSMLGEVGDFRAALE

>XP_030844030.1 uncharacterized protein LOC764526 [Strongylocentrotus purpuratus]

MLLSCGCTNRPSRLNSPKDWSIILNMSALKRSPRNVRSPRSTAAMVKHLPSAVAALLQSGKGPAKLTKVDTPDGVKAVPLADWLWDKSQVQAQAALDTDFIQGIKSGLL
DPTNYGGYTVQDAVYCDNATDYFGIAEMKACDKDLKEFIAARIKSYAEYTEIMFKEWYIKHPKGIAMGDAAASYSEFELDVATTEEPFYLLIAMLPCEKLWGWLAQEIK
SGINDTNVYSFWIQDNLPGSHTLANYIDENAESLKWI

>XP_019617645.1 PREDICTED: uncharacterized protein LOC109464968 [Branchiostoma belcheri]

MATSTKRSRYNVQSPRNRLANPRVLKAFLQATIAFQKAGKKDEVDSPDVLRATAQQRVADEKTLSERLWDQNLDLVDAAWDTKFIQGIAHGNLDPNDYGQYTVQDAA
YCNAATDNLQFLTDKVQGAPLEEFFKGQYEGYKGYTQELYESWFLKPDGADLGPAAQAYVDLEHEIAHNEEALYYLVSMIPCLRLWPYLAKMMEKGGYDKESNIYKF
WIEDNGSYKSAEEVEEVVDGCSLSGLINEEKANQIYRKCMYGEVNFFKSACDEPLLIIPPQ

>CAC5425173.1 unnamed protein product [Mytilus coruscus]

MWKSSPRCVSDAAVRELQPFTLSDKPNTDFDFCELMWTETKETRIKAFESNFVQGVRRGRLHPTSFGAYMVQDSVYCQRVADSLEVAAEGEKCGPLKRFLEERKSSYER
YYEDLFKLWHIRDGKAIGLGPECQFYVDTVAGVADKDDAHYMLVALIPCGRLWPWLGQKLNEAEHCFGAYTDWVNSNLNPNSKGYKTLQDMVNAAVSGRKIDKKKA
MDIYSKCMNGEAGFFGSVPI

>MCM1088325.1 hypothetical protein [Muribaculaceae bacterium]

MYRSWGIFSATERNHYEQMFDSEQDSFLNRAFAANQDIADNTYQTDFIQGIRKGNLSPDIYGSVSVLDAYYCYQAAQSIWFACGQAETDSELQNALKKFYNSYAKYNAT
FYNVWHVPAAQNVVPTNAFKTYAEHERNVAQNDDPIYLLPALLPCYALWAWMANKIDKDKSATPGVYKDWVDGNKGNDGVSGSAVLADQVIGNWIAAGKAFDEEK
AQAIFKESMQCECRVFSSLGKY

>WP_231892035.1 hypothetical protein [Burkholderia sp. MSMB1589WGS]

MKPLVTRTPRRIQLDHPSLAKLDVSTSPPPANSITSQLWEACYSLAQDALNSPYIQGIANGTLPPCNYGQYTVQDAAYCVRAEQDYRLVEARAKKHHEDMLAAFAQARY
EGYLSYTDSIMKSWHIKDILAINPNDAVKAYVAHEHYVAEFMEPIYGVVAMIPCDHLWSWLAETLSPDNVPNNLYDFWISDNQDWSGTYRLENFVNSWFAAHPKQYEW
ESALKAYRGSMLGEVGDFRAALE

>CAC5425770.1 unnamed protein product [Mytilus coruscus]

MWKETEKTRMEAFTSNFVQGVKDGILHPTSFGAYMVQDSVYCQRVTDSLKVAAEREKCGPLKRFLEQQKNDYEEYYEDLFKNWHIRDGKAIGLGKECQEYVDTVAGV
ADKDDAHYMLVALIPCGRLWPWIGQKLKEANHCFGAYTDWVNTNFKPTSEGYKKLEVLVNAALAKKKIEKQKALNIYSKCMNGEADFFGSVPI

>WP_086479089.1 hypothetical protein [Oceanospirillum sanctuarii]

MSPANGTANRSANTSASILTRSPRSRVPTSVFKKKPVKLAAATGETLYDQMWAATTQYQQAALASDFVQGIAHSDLNPDQYGQFTVLDAAYCSYSADDYSIVIPKATDP
EVKAFCIARQKDYLDYNAEVFAGWHIADPKAVQLTDLAQAYADFERTIAETMDPLYFVVAMTPCISLWTYIGLTLAPDATPNNLYASWIEANQVAEAPKDMADFINAHA
DQLDHQRAIEIFIKGMQFECNMFRSACGQPPI

>VBP05719.1 multifunctional hydroxymethylpyrimidine phosphokinase/4-amino-5-aminomethyl-2-methylpyrimidine hydrolase [Burkholderia pseudomallei]

MKPLVTRTPRRIQLDHPSLAKLDIPTSPPPANSITSQLWEACYSLAQDALNSPYIQGIANGTLPPCNYGQYTVQDAAYCVRAEQDYRLVEARAKKHHEDVLAAFAQARYE
SYLSYTDSIMKAWHIKDILAINPNDAVKAYVAHEHYVAEFMEPIYGVVAMIPCDRLWSWLAETLSPDNVPNNLYDFGISDNQGWSGTYSLENFVNSWFAAHPKQYEWE
SALKAYRGSMLGEVGDFRAALE

>OPL33125.1 hypothetical protein AM593_07642, partial [Mytilus galloprovincialis]

MWKTSPRCVSGAAVKKLLQCTLSDKPCNDYDFCEWLWTETKDTRIKALNTNFVQGVKDGMLHPTSFGAYTVQDAVYCQKAQKSLSVAAKREEMGPLKIFLDHESTDY
KSYYEDLFDKWHIRDGTAIELGKECQEYVDTVADVASKDDAYYMLVALIPCGRLWPWLGQQLNAAKHNFGAYTDWVNSNFDPTSEGYKKLEVRVNAAFSNQEIDEKK
ALSIYSKCMNGEASFFGSVPI

>VDI32424.1 Hypothetical predicted protein [Mytilus galloprovincialis]

MWKASPRCVSDAAVSNLQQCTLSDKPCDDYDFCEWLWTETKDTRTIALNTNFVQGVKDGMLHPTSFGAYMVQDAVYCLKAHESLSVAAKRAEMCPLKIFLDHESSSY
KSYYEDLFDKWHIRDGKAIELGKECQEYVDTVADVASKDDAYYMLVALIPCARLWPWLGQQLTAAKDNFGAYTDWVNSNFDPSSKGYKKLEVRVNAAFSNKQIDRN
KALNIYSKCMTGEAGFFGSVPI

>XP_015760767.1 PREDICTED: uncharacterized protein LOC107339930 [Acropora digitifera]

MNRDRQQGTKWQLKKLRKMRVNTSFLPAETLKSAWPSFSQELFNNETSVKIRNAAKESMFIQGMASGTLDPDEYGGYMVQDAAYCFNAVEAFDVAANKIQSEGKPEF
ALLYRVQSESYKKYNQEFVKVWQLKSTESIVMGPAAATYVDYESVLSLQDPKYLAIAMLPCTMLWPWIAGELIDSVHKDNPYYDWFAENKPDGHKSRLEEFVDYFFNA
GDKAKSLVIFHQGLVNELNFFRNACDQTLYYYSSFNV

>XP_021377162.1 uncharacterized protein LOC110465566 [Mizuhopecten yessoensis]

MVLRHLRLATNVHGLHKPVTKSTAVVIAVCLLLLVVPKTDAESLSEYLWNQTESIRNNALSTYFVQGIGNGTLNPTAFGSYMIQDSVYIYNAKRSIDSAVYRAPEGKLKE
FLRGKSASFERYYHALFKKWHIKDPADIKVCAACQSYADYEHQIAATEDTIYMIVAMLPCYKLWPWLGQQLQSANHGVYDDWFNSNFDPQYEGYKALDALVDAADVK
QTISRNKALEVYTRCMLGEYEFFDSVQETQ

>XP_027049566.1 uncharacterized protein LOC113677017 [Pocillopora damicornis]

MDVTPSSQIPSLKQEGSFSSALFNDPKSEIICNLAKESEYIQGMVKGTLDPNVFGSYTVQDAAYCFNAVDSFDRAAEKMQEVGKPEFSLLYRTQSESYRSYNQYFVQTWR
LQNTDSVAMGPAAATYVGFERALSQNDPKYLCIAMLPCTMLWPWIADQLIASVDKNNPYYVWFKDNKPSPGHKSHLEMFVDHFFVQVGPEERQKCLSIFQEGLVNEVN
FFRSACDQTLFYYSSFQE

>XP_033749938.1 uncharacterized protein LOC117334425 [Pecten maximus]

MALRNPRLSSHVNGKFKKFVKKTDAEPLSENLWNQTEGIRQETLRTYFVQGIGNGDLNPTDFGEFMVQDSVYCQKAKASIDIAAEKAFPGPLKEYLSKESQSYEKYYKTL
FKKWHIEDASGITLDDACKSYAAYENNVAVTEDTIYMIVAMIPCMKLWPWLGQQLESGKKGVYDEWYNDNFDPTYDGYKELDKFVDDAAITGSIDAQKALKVYAYCM
SGELAFFGSVQHK

>CAH1265317.1 Hypp3161 [Branchiostoma lanceolatum]

MADSTMRSKLSVHSPRSRLEQPHFLQAFLEATVKQQVDSAVGETLSELLWKENFDMAGKAWDTQYIQGMAFGTLDPRAFGHYTVQDAAYCKSSTDNLKFLVDKMDDD SLKKFFEGRYESYKRYTKELYDQWFLKPDGADLGKAAKWYVDLEYKVAQEGGLYYLVAMIPCLRLWPYLAKKMKAEDGNIYKFWIDENGSFHGAEKVEKVVNAHAS SIDSQKASRIYRKCMYGEVNFFRSACGQPLLNIPTDQ

>XP_038068553.1 uncharacterized protein LOC119737947 [Patiria miniata]

MIPRDSMRCRSNMYAQLFCVLQCCNATMTLLSAELTGTKIDSRESGPVNVQLSEWLWRGSESRALAQKALHSGFIKGIKSGLLDPRHFGSFFVQDAVFCFHVVQHLKIAL QKYPSDSTLTAVLQEQIRTNEKYTRDLFQQWHIANPNGILLGKAAEEYVKFEREVAEDMSPLYLLIAMLPCEKLWSWLAMELQYEISATNVYRFWVDDHVSIDPVLATFI DENAGRYNVDLDTAMFIYQKALYWEGEFFESASAGSN

>XP_044176822.1 uncharacterized protein LOC122959544 [Acropora millepora]

MNRDRQQGTKWQLKKLRKMRVDTSFLPVETLQSARPSFSQELFNNETSVKIRNAAKESMFIQGMASGTLDPDDYGGYMVQDAAYCFNAVEAFDVAANKIQSEGKPEFA LLYRVQSESYKKYNQEFVKVWQLKSTESIVMGPAAATYVDYESVLSRQDPKYLAIAMLPCSMLWPWIAGELIDSVHKDNPYYDWFAENKPDGHKSRLEEFVDYFFNAG DKEKSLVIFHEGLVNELNFFRNACNQTLYYYSSFNV

>XP_044176810.1 uncharacterized protein LOC122959541 [Acropora millepora]

MNRDRQQGTKWQLKKLRKMIVDTSLLPAETLQSARPSFSQELFNNETSVKIRNAAKESMFIQGMASGTLDPDDYGGYMVQDAAYCFNAVEAFDVAANKIQSEGKPEFA LLYRVQSESYKKYNQEFVKVWQLKSTESIVMGPAAATYVDYESVLSRQDPKYLAIAMLPCSMLWPWIAGELIDSVHKDNPYYDWFAENKPDGHKSRLEEFVDYFFNAG DKEKSLVIFHEGLVNELNFFRNACNQTLYYYSSFNV

>XP_044176820.1 uncharacterized protein LOC114950603 [Acropora millepora]

MNRDRQQGTKWQLKKLRKMRVDTSFLPAETLQSARSSFSQELFNSETSVKIRNAAKESMFIQGMASGTLDPDDYGGYMVQDAAYCFNAVEAFDVAANKIQSEGKPEFA LLYRVQSESYKKYNQEFVKVWQLKSTEGIVMGPAAATYVDYESVLSRQDPKYLAIAMLPCTMLWPWIAGELIDSVHKDNPYYDWFAENKPDGHKSRLEEFVDYFFNAG DKAKSLVIFHQGLVNELNFFRNACDQTLYYYSSFNV

>XP_044176818.1 uncharacterized protein LOC122959543 [Acropora millepora]

MNRDRQQGTKWQLKKLRKMIVDTSFLPAETLQSARPSFSQELFNNETSVKIRNAAKESMFIQGMASGTLDPDDYGGYMVQDAAYCFNAVEAFDVAANKIQSEGKPEFA LLYRVQSESYKKYNQEFVKVWQLKSTESIVMGPAAATYVDYESVLSRQDPKYLAIAMLPCSMLWPWIAGELIDSVHKDNPYYDWFAENKPDGHKSRLEEFVDYFFNAG DKEKSLVIFHEGLVNELNFFRNACNQTLYYYSSFNV

>XP_044176816.1 uncharacterized protein LOC122959542 [Acropora millepora]

MNRDRQQGTKWQLKKLRKMIVDTKLLPAETLQSARPSFSQELFNNETSVKIRNAAKESMFIQGMASGTLDPDDYGGYMVQDAAYCFNAVEAFDVAANKIQSEGKPEFA LLYRVQSESYKKYNQEFVKVWQLKSTESIVMGPAAATYVDYESVLSRQDPKYLAIAMLPCSMLWPWIAGELIDSVHKDNPYYDWFAENKPDGHKSRLEEFVDYFFNAG DKEKSLVIFHEGLVNELNFFRNACDQTLYYYSSFNV

>NRE46958.1 hypothetical protein [Burkholderia pseudomallei]

MAQDALNSPYIQGIANGTLPPCNYGQYTVQDAAYCVRAEQDYRLVEARAKEHHEDMLAAFAQARYESYLSYTDSIMKSWHIKDILAINPNDAVKAYVAHEHYVAEFM EPIYGVVAMIPCDHLWSWLAETLSPDNVPNNLYDFWISDNQGWSGTYRLENFVNSWFAAHPKQYEWESALRAYKGSMLGEVGDFRAALE

>RMX41355.1 hypothetical protein pdam_00012798 [Pocillopora damicornis]

MEFRAVQGNQKNPQTARKVTFHLSFFSPLTHDAQDGSLSSYLSNDSKSKSVYNQRKEDKMDVTPSSQIPSLKQEGSFSSALFNDPKSEIICNLAKESEYIQGMVKGTLDPN VFGSYTVQDAAYCFNAVDSFDRAAEKMQEVGKPEFSLLYRTQSESYRSYNQYFVQTWRLQNTDSVAMGPAAATYVGFERALSQNDPKYLCIAMLPCTMLWPWIADQLI ASVDKNNPYYVWFKDNKPSPGHKSHLEMFVDHFFVQVGPEERQKCLSIFQEGLVNEVNFFRSACDQTLFYYSSFQE

>XP_044176814.1 uncharacterized protein LOC114967375 [Acropora millepora]

MNRDRQQGTKWQLKKLRKMIVDTSLLPAETLQSARPSFSQELFNNETSVKIRNAAKESMFIQGMASGTLDPDDYGGYMVQDAAYCFNAVEAFDVAANKIQSEGKPEFA
LLYRVQSESYKKYNQEFVKVWQLKSTESIVMGPAAATYVDYESVLSRQDPKYLAIAMLPCTMLWPWIAGELIDSVHKDNPYYDWFAENKPDGHKSRLEEFVDYFFNAG
DKEKSLVIFHEGLVNELNFFRNACNQTLYYYSSFNV

>CAG2251094.1 unnamed protein product [Mytilus edulis]

MWKVSPRCVSGAAVSKLLQCTLSDKPCNDYDFCEWLWTETKDTRMKALNTNFVQGVKDGMLHPTSFGAYTVQDAVYCQKAQESLSVAAKRDKMGPLKIFLEHESTD
YKSYYEDLFDKWHIRDGKAIDLGKECQEYVDIVADVASNDDAYYMLVALIPCGRLWPWLGQQLTAAKHDFGAYTDWFKANFDPSYEGYKKLEVRVNAAFSKQEIDKE
KALGIYSKCMNGEANFFGSVPI

>XP_028657201.2 uncharacterized protein LOC114651558 [Erpetoichthys calabaricus]

MMTRMSILILSLLAMRSMVWSIDIQDPSAKWTSPGKCSQDSVYDQLWDKSQDIVGEVIKTNFLKEINDNTLIAERYMKLMIQDIYYIQGVFNALNIALKNTNMPDDVKKF
LEDRATSYKNFLEMMQENNNLKDASSIIPNKAAQEYVEEYEKIAKRTDEGLYMVIALLPCARLWPYISEKLSITRCSPYYQFKMDNAKDKSRDHYEKLLQEYKDKIDMDE
ALKIFQIQMRHELAFFQSVDPKN

>XP_021377138.1 uncharacterized protein LOC110465551 isoform X4 [Mizuhopecten yessoensis]

MTGAEPLSEYLWKQTEAIRNKALYTNFVQGIANGSLNPTAFGSYMVQDAVYCYKAKQSIDMSVSRAPDGELKDYLRKESTNYERYYQVLFLKWHIIDPVGVNMSEACE
SYADYEYQIAATEDTIYMLVAMLPCMKLWPWLGQQMSSFDHGVYTEWYNAYFDPQYDGYKALDIFVDEADASKTINRTKALQVFTRCMEGEYEFFNSVQPMQLPTTS
LPVDDHVTSGSVSCTRWTLIISMYIVYAVMFFTNCVFELSAFL

>MBW4672456.1 hypothetical protein [Cyanomargarita calcarea GSE-NOS-MK-12-04C]

MQQNLSFLSPRKFVPEMALETTEFFKTDGKRLSEIMWEKALPIAEQALQTCHIQGIKYGDLDPNKYGNYTVQDAIYCYQATEQYRKLAETTPDQQIKIFAEKQVNSYEQY
TKNLFDQWCIADPNGINLSPDLKKYIDYKSEVLDKYKSLYFIVANIPCLRIWWWLANQLQCRTVGPANLYSFWIKDNLSPSYNNLEDFVDSKADELDIEYQKKLPLLQIVL
PFVDADLSLSALTLVYSSVLSRNLFQ

>XP_021377137.1 uncharacterized protein LOC110465551 isoform X3 [Mizuhopecten yessoensis]

MALRNPRQSTNLESIHEPTYISTARVLAVCLWLLCIQQQINMTGAEPLSEYLWKQTEAIRNKALYTNFVQGIANGSLNPTAFGSYMVQDAVYCYKAKQSIDMSVSRAPDG
ELKDYLRKESTNYERYYQVLFLKWHIIDPVGVNMSEACESYADYEYQIAATEDTIYMLVAMLPCMKLWPWLGQQMSSFDHGVYTEWYNAYFDPQYDGYKALDIFVDE
ADASKTINRTKALQVFTRCMEGEYEFFNSVQPMQLPTTSLPVDDHVTSGSVSCTRWTLIISMYIVYAVMFFTNCVFELSAFL

>XP_020625043.1 uncharacterized protein LOC110062459 [Orbicella faveolata]

MSRIRERSRRNLKSPRKIKFNFNVLPQEESFPGRSSFSQDLFNNETSMKIRNAAKESKFIQGMATGTLDPDDYGGYMVQDAAYCFNAVGAFDYAAQQMQVQGKPEFSLL
YRVQSETYKSYNQEFVKTWRLKNTESVVMGPAAEMYVGYESALSRQDAKFLCIAMLPCTMLWPWIASELIDSVDEKNPYYGWFEDNKPDTNDKSRLEKFVDFFFTPED
KEKSLLIFHEGLVNELNFFRDACDETLYYYSYFNL

>WP_254564068.1 hypothetical protein [Oscillatoria sp. HE19RPO]

MLQKLSFRSPRQFVPEIAGGTTEFLKVDGKRLSTLMWESAKPIAEKTLQTCHIQGIKYGDLDPNHYGFYTVQDAIYCYQTTEQYRILAETATDANIKEFAEERQKSYERYT
KNLFDNWCIEDPNSIKLSPELQKYINYKSEILKSHSSAYFIVSNIPCVRLWTWLANQLQNRLVGAANLYSFWIQDNLSDKSALTLEDFVDKNAHALDINEAIAVYKRCMEG
EFEFFALAGKVRYFLQEGDFLLPGDRLFSENESYQLIYQGDGNLVVYDSSNGKAIWASNTQGTYAWRTYMQDDGNFVVYTDHAKPIWSSGVYGSQYKGSKLFLKNDGN
LVIYDTSKNPIWQSKN

>WP_128832243.1 hypothetical protein [Burkholderia pseudomallei]

MAQDALNSPYIQGIANGTLPPCNYGQYTVQDAAYCVRAEQDYRLVEARAKKHHEDVLAAFAQARYESYLSYTDSIMKAWHIKDILAINPNDAVKAYVAHEHYVAEFM
EPIYGVVAMIPCDRLWSWLAETLSPDNVPNNLYDFWISDNQGWSGTYRLENFVNSWFAAHPKQYERESALKAYRGSMLGEVGDFRAALE

>WP_152615976.1 hypothetical protein [Burkholderia pseudomallei]

MAQDALNSPYIQGIANGTLPPCNYGQYTVQDAAYCVRAEQDYRLVEARAKKHHEDVLAAFAQARYESYLSYTDSIMKAWHIKDILAINPNDAVKAYVAHEHYVAEFM
EPICGVVAMIPCDRLWSWLAETLSPDNVPNNLYDFWISDNQGWSGTYRLENFVNSWFAAHPKQYEWESALKAYRGSMLGEVGDFRAALE

>WP_154235112.1 hypothetical protein [Burkholderia pseudomallei]

MAQDALNSPYIQGIANGTLPPCNYGQYTVQDAVYCVRAEQDYRLVEARAKKHHEDVLAAFAQARYESYLSYTDSIMKAWHIKDILAINPNDAVKAYVAHEHYVAEFM
EPIYGVVAMIPCDRLWSWLAETLSPDNVPNNLYDFWISDNQGWSGTYRLENFVNSWFAAHPKQYEWESALKAYRGSMLGEVGDFRAALE

>XP_021377135.1 uncharacterized protein LOC110465551 isoform X1 [Mizuhopecten yessoensis]

MFSNYVLFEQQTDTCTMALRNPRQSTNLESIHEPTYISTARVLAVCLWLLCIQQQINMTGAEPLSEYLWKQTEAIRNKALYTNFVQGIANGSLNPTAFGSYMVQDAVYCY
KAKQSIDMSVSRAPDGELKDYLRKESTNYERYYQVLFLKWHIIDPVGVNMSEACESYADYEYQIAATEDTIYMLVAMLPCMKLWPWLGQQMSSFDHGVYTEWYNAYF
DPQYDGYKALDIFVDEADASKTINRTKALQVFTRCMEGEYEFFNSVQPMQLPTTSLPVDDHVTSGSVSCTRWTLIISMYIVYAVMFFTNCVFELSAFL

>XP_021377136.1 uncharacterized protein LOC110465551 isoform X2 [Mizuhopecten yessoensis]

MFYLNSKQILALWHYGTQDSPPTWRVYTKVLAVCLWLLCIQQQINMTGAEPLSEYLWKQTEAIRNKALYTNFVQGIANGSLNPTAFGSYMVQDAVYCYKAKQSIDMSV
SRAPDGELKDYLRKESTNYERYYQVLFLKWHIIDPVGVNMSEACESYADYEYQIAATEDTIYMLVAMLPCMKLWPWLGQQMSSFDHGVYTEWYNAYFDPQYDGYKA
LDIFVDEADASKTINRTKALQVFTRCMEGEYEFFNSVQPMQLPTTSLPVDDHVTSGSVSCTRWTLIISMYIVYAVMFFTNCVFELSAFL

>WP_143281142.1 hypothetical protein [Burkholderia pseudomallei]

MAQDALNSPYIQGIANGTLPPCNYGQYTVQDAAYCVRAEQDYRLVEARAKKHREDVLAAFAQARYESYLSYTDSIMKAWHIKDILAINPNDAVKAYVAHEHYVAEFM
EPIYGVVAMIPCDRLWSWLAETLSPDNVPNNLYDFWISDNQGWSGTYRLENFVNSWFAAHPKQYEWESALKAYRGSMLGEVGDFRAALE

>WP_111952238.1 hypothetical protein [Burkholderia pseudomallei]

MAQDALNSPYIQGIANGTLPPCNYGQYTVQDAAYCVRAEQDYRLVEARAKKHHEDVLAAFAQARYESYLSYTDSIMKAWHIKDILAINPNDAVKAYVAHEHYVAEFM
EPIYGVVAMIPCDRLWSWLAETLSPDNVPNNLYDFWISDNQGWSGTYRLENFVNSWFAAHPKQYEWESALKAYRGSMLGEVGDFRAALE

>WP_143292595.1 hypothetical protein [Burkholderia pseudomallei]

MAQDALNSPYIQGIANGTLPPCNYGQYTVQDAAYCVRAEQDYRLVEARAKKHHEDVLAAFAQARYESYLSYTDSIMKAWHIKDILAINPNDAVKAYVAHERYVAEFM
EPIYGVVAMIPCDRLWSWLAETLSPDNVPNNLYDFWISDNQGWSGTYSLENFVNSWFAAHPKQYEWESALKAYRGSMLGEVGDFRAALE

>XP_045192634.1 uncharacterized protein LOC123548979 [Mercenaria mercenaria]

MKLWLFLIAFGLVSVSLECSTVKPPEVSLSDYLWQETVQQRQAALASSFIQGLKMVSLDPTNFGAYMVQDSVYCYLVKTNLDLAATRVKNNPQLKQFLEARSSSYEQYY
KALFKAWHISNANGISLGTAIAGYMKHLDNVASKMDPIYLVAALIPCARLWPWLGGQIGADTRNFGVYSKWVTENLNPGSHGYEKYEAVINNAAEQGLIDSQMALQVY
TRSMQGEVGFFSSV

>MCM1144885.1 hypothetical protein [Lachnoclostridium sp.

MYRSWGIFSATERKHYEQMFDAEQDSFLKRAFAANQNIADNTYQTNFIQKIWKGDLPPDEYGSVSVLDAYYCYQAAQSIWFACGKAETDSELQNALKKFYNSYAKYNA
AFYNVWHVPAAQNVVPTNAFKTYAEHERNVAQNDAPIYLLPALLPCYALWAWMANKIDKDKSATPGVYQRWVDGNKGNDGVSGSAVLADQVISHWIAAGKAFDEG
KAQAIFKESMQCEYRVFSSSGKY

>WP_108033887.1 hypothetical protein [Burkholderia mayonis]

MAQDALNSPYIQGIANGTLPPCNYGQYTVQDAAYCVRAEQDYRLVEARAKEHHEDMLAAFAQARYEGYLSYTGSIMKSWHIKDILAINPNDAVKAYVAHEHYVAEFM
EPIYGVVAMIPCDHLWSWLAETLSPDNVPNNLYDFWISDNQGWSGTYRLENFVNSWFAAHPKQYEWESALKAYRGSMLGEVGDFRVALE

>WP_122632739.1 hypothetical protein [Burkholderia pseudomallei]

MAQDALNSPYIQGIANGTLPPCNYGQYTVQDAAYCVRAEQDYRLVEARAKKHHEDVLAAFAQARYESYLSYTDSIMKAWHIKDILATNPNDAVKAYVAHEHYVAEFM
EPIYGVVAMIPCDRLWSWLAETLSPDNVPNNLYDFWISDNQGWSGTYSLENFVNSWFAAHPKQYEWESALKAYRGSMLGEVGDFRAALE

>XP_039608874.1 uncharacterized protein LOC120528863 [Polypterus senegalus]

MQLESSVFSYWQYVAECSHDSIYDQLWDKSQDIVGEVIKTNFLKEINDNTLIADRYMKLMIQDIYYIQGVFNALNIALKNPNMPDDVKHFLEDRARSYKNFLEMMQENY
NLKDANSIIPNKAAQDYVEEYVNIAKRKDEGLYMVIALLPCARLWPYISEKLSITRCSPYYQFKMDNAKDKSRDHYEKILQEYKDKIDMNLGLNIFQTQMKHELAFFQSV
DSKN

>XP_028412868.1 uncharacterized protein LOC114535758 [Dendronephthya gigantea]

MTSSQAILLLTMVVSLTNIFCYAPQCFQNEFLNKKAIQGHRLKDSNTGLTFAQSLFEDPFSVNVRNAVLETKFFQGIIQNTLEPEQYGGYMVQDAAFVFDAVKAFDTAAE
NMRGEYPPDFALFYHGRSECFTSYASYFVSKWKLFSASSILMGPAVGTYVAYQMKLAQTQPKNLAIGILPCEMLWPWVAAQIDPQVPEKNVYRSWVDDNLDNGYSGAQ
TFANKFFSEGDKDASQIIFNEGIINELNFFRSACGEDPVDYDFGNKNETAPIV

>VBL77151.1 ISBma1, transposase [Burkholderia pseudomallei]

MKPLVTRTPRRIQLDHPSLAKLDIPTSPPPANSITSQLWEACYSLAQDALNSPYIQGIANGTLPPCNYGQYTVQDAAYCVRAEQDYRLVEARAKKHHEDVLAAFAQARYE
SYLSYTDSIMKAWHIKDILAINPNDAVKAYVAHEHYVAEFMEPIYGVVAMIPCDRLWSWLAEMTCLISSGHFNLGERPRLLQLNNTAA

>XP_028412847.1 uncharacterized protein LOC114535738 [Dendronephthya gigantea]

MIRGNFSRDFGKKAGFGKTIQGHRLKDSETGLTFAQSLFEDSFSVNVRNAVLETKFFRGIINNTLEPEQYGGYMVQDAAFVFDAVKAFDTAAENMQGEQPPDFALFYRGR
SESFTSYSSYFVSEWNLFSTSSIFMGPAVGTYVAFQMKLAQTQPKNLAIGILPCDMLWPWVAGQIDNQVEKTNVYRSWVDDNLGDGSSSAQTFANKFFTEGDKDASQKIF
NEGIINELNFFRSACGEDPVDYDFGYKNETAPIV

>XP_020903754.1 uncharacterized protein LOC110242137 [Exaiptasia diaphana]

MQDSTRRYPKTSRRMLFEIDHFPLEELYTSPDDVDSPLTTFTQDLFNSQASVIIREAAKGSSFIQGMAQGTLDPDIYGGYMVQDAAYCFXAVQTFDMAADSCQKSGNPEF
ALLFRVYSEKFKTYNQDFVKKWRLKNSDSVGMGPAAAMYVSYERSLGKSQDPRFVSIAMLPCQMLWPWIAGELIGSVNKDNPYYGWFNDNKPDPSGKKSTVERFVNR
CFTKPEDKQKAEEIFLEGMVNELNFFRDACGEGLRYNFGKLTVGFGRK

>XP_043938478.1 uncharacterized protein LOC122811025 [Protopterus annectens]

MKPGLVFLVISAGIISVKLEPYIQSNYKSESSNEVYQQTLYEKLWESGLDIADQTLQTDFLKQMQAGTLKAERYINFTLQDIYYAVKVTKILNRLSKRNFSQDETDLQQFFS
KTNQSYSHFADTLLIGYSLKNIHELRPWPVIDNYVRSYEKLLKRNPLYFAVGMLPCSKLWPYIANKLTIDTSSPYYHFKSENEHDGSKNNYESLLERHRLKISERQAKKIFQ
THMKLEMQFFAEA

>XP_046871482.1 uncharacterized protein LOC124463741 [Hypomesus transpacificus]

MADIEKHIYDDLWNSSFDIVRQILKADFLQGMASVRLSAERFMTFNLQDIYYLVKVTEMLAEMKGKPQTPPDLKTFFENKYNSYQRFAASVLKKYCLKDASSIIPGPAISS
YLQTYREAMKSDPIYFVVAMLPCVSLWPYLAQHLDIKKNTAYYEWKDENMHGDPRTAYQPLLDKYACSINRETAKTLFRKQMSHEQSFFMASITARPADALLGVCRST
VDHRHIFMPFSY

>KAH3819073.1 hypothetical protein DPMN_120803 [Dreissena polymorpha]

MARSPRSATHLVHHLEAHHFLKPSVIPLKTDVPQLAEYLWAQTTAYQKAALQSGYVQGLKSVALNPEGFGGYMVQDSVFCFKAQGNIDIAVTNATDAILRKFLEARSES
YKAYYQSLFDTWHIEDASGIKLGTECTEYVNHEHNVATTMNSIYFVVAMIPCAKLWPWIGQQINAIGGSFGVYDEWVKANFNPDSSGVEKYEKLINTAEQDGHIDRKIAL
DVYTASMKGEAGFFGSIKVK

>VDI50242.1 Hypothetical predicted protein, partial [Mytilus galloprovincialis]

MWKASPRCVSGAAVSKLLQCTLSDKPCNDYDFCEWLWTETKDTRMKALNTNFVQGVKDGMLHPTSFGAYIVQDAVYCQKAQESLSVAAKRDKMGPLKIFLEHESTDY
KSYYEDLFDKWHIRDGKAIELGKECQEYVDIVADVASNDDAYYMLVALIPCGRLWPWLGQQLIAAK

>VDI50241.1 Hypothetical predicted protein [Mytilus galloprovincialis]

MWKASPRCVSGAAVSKLLQCTLSDKPCNDYDFCEWLWTETKDTRMKALNTNFVQGVKDGMLHPTSFGAYIVQDAVYCQKAQESLSVAAKRDKMGPLKIFLEHESTDY
KSYYEDLFDKWHIRDGKAIELGKECQEYVDIVADVASNDDAYYMLVALIPCGRLWPWLGQQLIAAKVYI

>KAH3823320.1 hypothetical protein DPMN_125119 [Dreissena polymorpha]

MSTNMSPRHSTFDAHILGSHSLHKGPLTSPVPGAETLSDFLWSATEHYRTKALNSGFVQGMVKKSLDPEGFGYYMIQDCIFTFKAKTSFDLAAQRADDVSVKQLLIKKAE
DFTAYYQGLFAKWHIKDPSGIELGPECTEYVNHEQDVATHKKSIYFIVAIIPCAKLWPWIGEKIKAKVVRNLSC

>RMX41353.1 hypothetical protein pdam_00012794 [Pocillopora damicornis]

MEFEKVIRKDKRSPSLLSYFKPDSHFAGNVSTLFNDPNSEIIRNRAKESKFIQGMAKGSLDPDVYGGYMVQDAAYCFNAVDSYERAAEKVQQVGKPEFSLLYRTQSESFK
RYNHDFLQKWRLRNSDSVAMGPAAATYVGYERALSQNDPKYLCIAILPCTMLWPWIAGQLIASVEKNNPYYV

>MCG8574541.1 hypothetical protein [Flavobacteriales bacterium]

MWKASENIAQQALNTDFIQGIKNGTLNPVTYGAFNISDAYYCVHGAADYLTAANKATHSVLKAFLLKKHKSYQQYNDTFPKAWRIKDANGIVPS

>XP_015217116.1 PREDICTED: uncharacterized protein LOC107079203 [Lepisosteus oculatus]

MKSFMVLSYFCAWSVFALDPKENVVNTKHCDRHGVSLYEKLWKNNMDIARDTMKTDFLKRMEAGTLEAERYINFTLQDIYYLEEVTKLLQKLSKEDELREDLKSFIMG
RYNSYSSFEKYSLQMYQLKNTSAIIPSPTIANYIQSYKAISEDDHVYFAIALLPCSKLWPYVAQNLKISKKSPYYPFKKNNVEDNSKKHYENLLEKYRCNIDESKAQSLFRL
QMAHEREFFSTS

>KAH3823321.1 hypothetical protein DPMN_125119 [Dreissena polymorpha]

MSTNMSPRHSTFDAHILGSHSLHKGPLTSPVPGAETLSDFLWSATEHYRTKALNSGFVQGMVKKSLDPEGFGYYMIQDCIFTFKAKTSFDLAAQRADDVSVKQLLIKKAE
DFTAYYQGLFAKWHIKDPSGIELGPECTEYVNHEQDVATHKKSIYFIVAIIPCAKLWPWIGEKIKAKVGKRVSQHIPFLVCFIILRSVITSIQSPPMMSVRTQARV

>XP_033751116.1 uncharacterized protein LOC117335234 [Pecten maximus]

MGTNFDELWKSTEAKRNDVLKTTFVDGVGAGTLDPVKFGGFVIQDCVYLYEQYKCIKAAAQRATDKDLKKFLSDVAGKYDGYYRDAFKAWHIEQASGIELGTACQAY
VKYMQDAQKLDTIYFLVSMTPCLSLWAWLGEQLQKKNKGVYAGWVKNNFAGGSTLKRLEKEINEAKLDIGIAKNLFKDGMDHEYKFFSSA

>XP_046846899.1 uncharacterized protein LOC124440549 [Xenia sp. Carnegie-2017]

MTKWNEILLLSIILLLSIVSAFKLQNQVDSRILAYLRHGASNYRPGVTASNNEEFAQSLFDNQFSINVRNAVLQTQFFQKMINNTLQPEEYGGYMVQDAAYVFDAIYAFQI
AAKKSQASDSLPRDFALFCRGRAESFTSYYSYFSSEWKLRNSKSVIMGPAAATYVAFEMKLAKTDAKFLSIGILPCDMLWPWVARELSNLVGESNVYRSWVDDNYSEEG
SSSSTQDFVNKFFTEDDRAISQEIFNEGMINELNFFRSACGEDPVDYDFGDSPQA

>XP_001636921.2 uncharacterized protein LOC5516928 [Nematostella vectensis]

MGGRRTNFKIINIPRSGVKPADKLVGIQKSTAQPLHAPSAAPFPENKASFTQKLFEHPKSQAIVDECKRTKFMRGMAEGTLLADLYGGYMVQDPAYCYHAVPAYELAAD
KLQSEFPEFSLLYRRQSGSFQKYNQDFVNTWRIKGADSIVLGEAAKTYVKYESAISQSQSRKLCIAMLPCQMLWPKLANDMGGEVREGNPYKPWFDGNKSDHPSHLERFI
DYFLRDEKVRDYLDEFLEGMTNELNFFRDACGEELYYYTKEFQM

>MBD5492913.1 hypothetical protein [Lachnospiraceae bacterium]

MFRSWGLVFESEIEYYEKLSVSEEYSFLEKAFAANMDIAENTYNTPFVQSIKADKLSPETYGSVTVLDAYYCYEAAQSIWHACGNADENSELQNALKKFYNSYAKYNGT
FYSTWHVPAAQNVVPNNAFQTYAKHERAIAKEDSPIYLLPALMPCYKLWAWMAYKIYQEWKAESSSNKQNVGDDKLYGFWVLGNMGNKGESGSATLADHVISQWVK
DGKEFDEEKALKIFRKSMQCEYDVFSSNGNY

>MCO5554051.1 hypothetical protein [Adiantum nelumboides]

MAAQLDSSISQPYRSGRSFRRLHLRTELPASPASPSPIPEPAASRPWLSSPAAGAGTSSTPSEFWQKLWADPKSEELKEQALSTLFINGVGLGTLDPRIFGKYSVQDAVYCA
KIAHLWFKLSNNPNADEYLQDLARVLSQKYRGIAKEMLQRWHIQTNESNEHFGVWLGDHATEYVKMVEESLEENPAYMLVATFPCLKLWPYLASTLEHTQPSNNIYAF
WIEENKKEGSSIERVQSAINLYAEKLDYATALELFHKGLQCEINFFNEP

>XP_020602320.1 uncharacterized protein LOC110041381 [Orbicella faveolata]

MTNTFQSRNKDQSYTPNRIRLNLNTLLQERSQDISKKVLRPRSSRSFSKVLFENVRLSVPIRQHALQTNFIQGMAVGSLKPTQYGRYMVQDTAYLANANKVYSEAAQKM
EEQGKPDFAFFYRSQAAKYEDFYKEFLKTWALESAEAVHEGPAVQAYMGYQQAVVKQHPRYLPVAMLPCTMLWPWMADSLIKTVDKQNPYYEDWFVKNLRAPGTQS
STERFVDENFSAFDEGIALEIFCEGMMNELNFFREACDEAPYKLSDICPSAG

>XP_028407799.1 uncharacterized protein LOC114530377 [Dendronephthya gigantea]

MMMSTEVILFLTMVVSLTNISCYELHHLQRKQLKENVDGAMGRMGSQRGDLWEISDAARPAFHNIDDSRLSRLRHYVPGFDERDENNPNELTFAQSLFEDPFSVKVRQA
VLETKFFQRIINNTLAPEQYGGYMVQDAAYVFDAVKAFDSAAKNMQGKHPPDFALFYRGRSASFTSYASYFVSKWKLKSTKSIITGPAAATYVAYEMKLAQNKPKYLSI
GILPCDMLWPWVAKQINDQVPKTHVYRSWVDDNLSDGSSSAQKFANKFFTAEDKEDSQPIFNEGIINELNFFRSACGEKPVSYEFGIQE

>XP_020602281.1 uncharacterized protein LOC110041341 [Orbicella faveolata]

MLLSNTRLVILRILKLTFKPSTMKVGKVWFFLVVAVSVATVVSSRQVDSGTGGQELDQKLSNHSDGTPGNKDQSYTPNRIRLNLNTLLQERSQDISKKVLRPRSSRSFSKV
LFENVRLSVPIRQHALQTNFIQGMAVGSLKPTQYGRYMVQDTAYLANANKVYSEAAQKMEEQGKPDFAFFYRSQAAKYEDFYKEFLKTWALESAEAVHEGPAVQAYM
GYQQAVVKQHPRYLPVAMLPCTMLWPWMADSLIKTVDKKNPYYEDWFVKNLRAPGTQSSTERFVDENVTAFDEGIALEIFCEGMMNELNFFREACDEAPYKLSEICPS
AG

>XP_033751089.1 aminopyrimidine aminohydrolase-like [Pecten maximus]

MDTRATHTVNDPKTKLSARLWESTKGIRDMAINTDFVQGIKNGKLDPEKFGVFCVEDSIYLYKQQHNIKVATEKAKPYPDQYTYLHKRIDSYKTLYEAEFKKWHVGKPE
GISLGKDLKEYMDYEEDVALNRQSIYFLVALIPCLKLWPWLGQQIKDGNHGIYNDWAKANLDPDYVGFKTVDALIDEADEKGGIQEDEAMKIYTKCMEGEYKFFNSI

>CAC5368566.1 unnamed protein product [Mytilus coruscus]

MLVILLQLVITLVQSTTRTPECLHFSSKTQGNNHISQGFGKRIPKPTGTGNECSHNLSHWLWHETEHFRTWAMNSTFVTGVRDGTLHPTSFGIFMLQDAIFCLEMSKSFQV
AANQLSDGSLKSLLEHEVKSYKRCGDELLTQWHIRNAPAIVVGAECQEYIDNHVNATRDEHPIYGIVAAIPCLRLWPWIGQQINLQNHQYGVYTDWVNYNFDPTYDGYK
KLEVMTNEFYAKGLIDKDKALHYYAKSIEGEAAFFDSVKP

>MCO5605367.1 hypothetical protein [Adiantum nelumboides]

MAAHVDRSICQPYRRWLHLCTEVPATSPPAVADRSSSNSKPPLQHPSLLSSVAGTPSTSDLWQKLWTDPKSEELKAQALNTLFISGVGLGILDPRIFGKYTVQDAVYCAKI
ANLWLNVSKNPNADAYLQDLARALSKKYLEVATEMLQWWQIQTDGVWLGEHAREYEQMVEESVKENPAYMLVATFPCLKLWPFLASTLERKQRRNNMYAFWIEGN
KQEGSTIARVQNAINRYAEKLDYATALRLFHKGLQCEINFFNEP

>XP_044166690.1 uncharacterized protein LOC122950761 [Acropora millepora]

MLVANVLVFLIPAAVLVATVFSPTEVFGANRKLHLKKPSKHLLEISDSGPKRSTEQNSFSQRLFDGPESQKIRELTKETAFITGMESGLLNANYYGAYMVQDIAYLANGAE
AYKNAADLMKDEFKTFYTEMATKWGSEYLKPMLTAWHLKDAGNVEPGTAAKEYMSFLITVSRGQPKYLAIAMLPCSMLWRWMADQLVDSVPVDSAYYSWFQDNRS
PSPGYKGSLERFVDANFDQEEYDTAKPFFCKAMVHECSFFSEGGNQTLCDLPPGCA

>EDO44858.1 predicted protein [Nematostella vectensis]

MGGRRTNFKIINIPRSGVKPADKLVGIQKSTAQPLHAPSAAPFPENKASFTQKLFEHPKSQAIVDECKRTKFMRGMAEGTLLADLYGGYMVQDAAYCYHAVPAYELAAD
KLQSEFPEFSLLYRRQSGSFQKYNQDFVNTWRIKGADSIVLGEAAKTYVKYESAISQSQPRKLCIAMLPCQMLWPKLANDMGREVREGNPYKPWFDENKSDHPSHLERFI
DYFLRDEKVRDYLDEFLEGMTNELNFFRDACGEELYYYTKEFQM

>CAC5368565.1 unnamed protein product [Mytilus coruscus]

MLVILLQLVITLASYGTVGDYMQCPAVQSTTRTPECLHFSSKTQGNNHISQGFGKRIPKPTGTGNECSHNLSHWLWHETEHFRTWAMNSTFVTGVRDGTLHPTSFGIFML
QDAIFCLEMSKSFQVAANQLSDGSLKSLLEHEVKSYKRCGDELLTQWHIRNAPAIVVGAECQEYIDNHVNATRDEHPIYGIVAAIPCLRLWPWIGQQINLQNHQYGVYTD
WVNYNFDPTYDGYKKLEVMTNEFYAKGLIDKDKALHYYAKSIEGEAAFFDSVKP

>XP_041096159.1 uncharacterized protein LOC121307898 [Polyodon spathula]

MQRVKKDSPARMNSFVIFTLLCTWSVFASSPRDKWTSKHCSSESSSLYDVLWDDSQVIADKTLETDFLKQMANGTLEAERYINFTLQDIYYLVKVTELLKALSIQPETHTD
IKQFILDRYNSYKKFADYVLNQYLLKNADDIKPSTAIAEYIQSYNDVMKEDHIYFAVALLPCARLWLYVAEHLEITKGSPYYRFKSENEVGHPEKHYKDLLENHRSEIDES
KAKNIFQKHMKHEYNFFRSS

>XP_039608768.1 uncharacterized protein LOC120528671 [Polypterus senegalus]

MPIENREELSLYDLLWDHSMDIVNLIMKTNFLTDMNLNTLQAERYTNMTIQDVYYIKAVYDILSTVVNEPWLPEDLKEFFSHIADGYYRFYKEMLIETRLKNTDSIVPSEA
VAEYVSSYQRVAQLERNKIYMVIALLPCSRLWPYISEHLNLTEDSPYYAFKKNNAKDGSREKYEKLLEDHRKEICENTALEIFRTQMNCERKFFTSF

>MCG8454917.1 hypothetical protein [Holophagales bacterium]

DPNVYGQYTLQDAVYCHYGQDDYRVLEQRAAADGEAAMAAFAKARYESYAKYNAETFEAWHIRNADAVALSEPEQVYIDFEHQVATSQAPIFGLIAMIPCDQLWAWL
ATELQGQAGPANLYSFWIHENADWGGAYRLDNFVDAWVAAHSGACDPATALFVYRSCMTCEVNLFRYACGQKLLPMPG

>XP_015766209.1 PREDICTED: uncharacterized protein LOC107345038 [Acropora digitifera]

MLGADVLAFLIPVAVLVATVLSPTEVFGANKKLHLKKPSKHPFEISQSGPKRSAEQSSFSQSLFDETESQNIRELTKETVFIRGMASGLLNANDYGAYMVQDIAYLANGAE
AYKNAADLMKDEFKTFYTEMATKWGSEYLKRMLTAWHLKDAGNVEPGTAAKEYMSFLITVSKGQPKYLAIAMLPCSMLWRWMADQLVDSVPVDSAYYKWFADNKS
PSPDYKGSLEKFVDANFGQEEFKTAKPFFCKAMVYECSFFSESGNQTLCDLPPGCA

>VDI43265.1 Hypothetical predicted protein [Mytilus galloprovincialis]

MNGMCIVLFILAFASIGTCQSVQSTARTPECLHFSSKTSGNNHISQGFGKRIPKPTGTGHECSHNLSHWLWHETENFRTWAMNSTFVTGVRDGTLHPTSFGIFMLQDAIFCL
EMSKSFQVAANQLSNGSLKSLLEHEVKSYKRCGDELLTEWHIRNAPAIVVGAECQEYIDNHVNATRDEHPIYGIVAAIPCLRLWPWIGQQINLQNHQYGVYTDWVNYNF
DPTYDGYKKLEVMTNEFYAKGLIDKDKALHYYAKSIEGEAAFFDSVKP

>XP_043938556.1 uncharacterized protein LOC122811066 isoform X1 [Protopterus annectens]

MNAVILTITMVLPRLITAEENNWTSEECSKESKSLLSTLWDNNIDIVKETEHTDFLICMTNGSLVAERYMKFILQDIYYVKHLTKLLSILANSSEPRKDLHEFFNNRNASYSR
FATSLLKGFGFQDFSELLPSAAIKAYIESYQALMMEDPIYFVIGLLPCAMLWPYIAQNLQISDSSPYINFKINNMADNSRKHYEKLLETHRHTMDEKKANEIFRTQMEHEKS
FFAES

>XP_043938557.1 uncharacterized protein LOC122811066 isoform X2 [Protopterus annectens]

MDAVILTILIVLPRLITAEENKWTSEKCSKESKSLLSTLWDNNIDIVKETEHTDFLICMTNGSLVAERYMKFILQDIYYVKHLTKLLSILANSSEPRKDLHEFFNNRNASYSRF
ATSLLKGFGFQDFSELLPSAAIKAYIESYQALMMEDPIYFVIGLLPCAMLWPYIAQNLQISDSSPYINFKINNMADNSRKHYEKLLETHRHTMDEKKANEIFRTQMEHEKSF
FAES

>XP_015766211.1 PREDICTED: uncharacterized protein LOC107345040 [Acropora digitifera]

MLGANALAFLIPVAVLVATVFSPTEVFGANRKLHLKKPSKHLLEISYSGPKRSAEQNSFSQRLFDEPESQKIRDLTKETVFIRGMESGLLNANYYGAYMVQDIAYLANGAE
AYKNAADLTKDKFKTFYTEMATKWGSEYLKPMLTAWHLKDAGNVEPGTAAKEYMSFLITVSKGQPKYLAIAMLPCSMLWRWMADQLVNSVPVDSAYYSWFQENRSP
SPGYKGSLERFVDSNFGQEEFETAKPFFCKAMVHECSFFSESGNQTLCDLPPGCA

>MCO5571000.1 hypothetical protein [Adiantum nelumboides]

MAALLDRSISQPYRSGRSALRLHLCELPSPPAPAVVDRPSPIPDQPSSPVLPSPAAGAGTATPDFWQKLWTDPKSEELKAQALSTLFVSGVGLGTLDPRIFGKYTVQDAVY
CAKIAHLWFNLSNNSNADEYLQDLARVLSRKYRDIATEMLQWWQIQSDESNEHFGVWLGDHATEYVKMVEGSLEKNPAYMLVPTFACLKLWPFLASTLEPKQPSNNIY
AFWIEEHKQEGSSIKLVQKAINLYAEKLDYATALQLFHKGLQCEINFFTKP

>XP_029210462.2 uncharacterized protein LOC114974375 [Acropora millepora]

MLGADVLAFLIPVAVLVATVLSPTEVFGANKKLHLKKPSKHPFEISQSGPKRSAEQSSFSQSLFDETESQNIRELTKQTVFIRGMASGLLNANDYGAYMVQDIAYLANGAE
AYKNAADLTKGKFKTFYTEMATKWGSEYLKPMLTAWHLKDAGNVEPGTAAKEYMSFLITVSKGQPKYLAIAMLPCSMLWRWMADQLVDSVPVDSAYYKWFADNRS
PSPGYKGSLEKFVDANFGQEEFKTAKPFFCKAMVYECSFFSESGNQLPCNLPPGCA

>XP_018610467.1 uncharacterized protein LOC108935949 [Scleropages formosus]

MNSFLFLTFSGVCYSLVDSAHVKWTPGQDVEDSSSFYEDLWKSNIDIANATLQTRFLQAMQMGNLPVEKYTIFGMQDLYYMINVTAMLREMSEKKPMPEDIKQFFKGRF
NSYEKYLAYLLNDFSLTNESVIVPRPAMASYINNYHRVMKKYEPIYFVVALLPCSKLWPYLAENVNMTENNPYYSFKRDNQGGNPAKHFKGLLDGYQHKMNKKTAHQI
FRQQMEHEKNFFQSSYTEKPILF

>KGC51248.1 putative transcription activator [Burkholderia pseudomallei]

MQDAAYCVRAEQDYRLVEARAKKHREDVLAAFAQARYESYLSYTDSIMKAWHIKDILAINPNDAVKAYVAHEHYVAEFMEPIYGVVAMIPCDRLWSWLAETLSPDNV
PNNLYDFWISDNQGWSGTYRLENFVNSWFAAHPKQYEWESALKAYRGSMLGEVGDFRAALE

>AIO12757.1 putative transcription activator [Burkholderia pseudomallei]

MQDAAYCVRAEQDYRLVEARAKKHHEDVLAAFAQARYESYLSYTDSIMKAWHIKDILAINPNDAVKAYVAHEHYVAEFMEPIYGVVAMIPCDRLWSWLAETLSPDNV
PNNLYDFWISDNQGWSGTYRLENFVNSWFAAHPKQYEWESALKAYRGSMLGEVGDFRAALE

>XP_038153220.1 uncharacterized protein LOC119791271 [Cyprinodon tularosa]

METVKLSCVVLGLLWMFSSISAHSEDIHDNQESCSGNAKWCSSSAHKVDPDYYEELWEKNLDLAKQTLKLPFLQHMQLGDLQADDYTVFMIQDIYYLAKVTDMLEVM
SKKDMPEDLKEFMKGRYQSYDRFRIQMLKTFNLNGVDGIKVTEAMKGYLAEYQRIMVEEEPIMFAVSLLPCNRLWVWIANQLNIGYGNAYWSWKKNNMGGKPEKKD
LLSKYLTAKNFEKANKIFRNQMGNELQFFKASLNQ

>VDI32426.1 Hypothetical predicted protein [Mytilus galloprovincialis]

MVQDAVYCLKAHESLSVAAKRAEMCPLKIFLDHESSSYKSYYEDLFDKWHIRDGKAIELGKECQEYVDTVADVASKDDAYYMLVALIPCARLWPWLGQQLTAAKDNF
GAYTDWVNSNFDPSSKGYKKLEVRVNAAFSNKQIDRNKALNIYSKCMTGEAGFFGSVPI

>KTF92611.1 hypothetical protein cypCar_00003475 [Cyprinus carpio]

MSYSQAVDDVYEYLWEQNKDIANKTIGLDFLRQMENGSLQAERYVNFTIQDIGYLLEVTKMLKKMSAKVFQPSDIKDFMKGRYSSYKGYADIILKQYFFKGEPPIVQTPA
MKKYLAYYRNLMHNEKSLYFAVGLLPCARLWVWLAKNLNTPPTNAYYTWKVDNMGGHPEKTYRALLNKYLKTPEKVKKANTVFRAQMQNEHDFFFSS

>XP_021375624.1 uncharacterized protein LOC110464623 [Mizuhopecten yessoensis]

MTSNDRGSNFDFLWNGSEKQWKQALSTPFVQGVKDGSLDAIEFVRYVVQDSIYLQKMYEVIKKAASKAEEGAMKDFLKLVTTKFKGHYEAAFNRLHINNSSAIKLDSA
CQAYIDFVENAAQNMDTIYFLVSMSPCLKLWAWLGTQIGTNHGAYNQWVASNFVSGSTLAGIIEFIDKEGEKFDRTKAMEVFKTGMEHEINFFNSVGKQ

>WP_004553849.1 hypothetical protein [Burkholderia pseudomallei]

MQDAAYCVRAEQDYRLVEARAKKHHEDVLAAFAQARYESYLSYTDSIMKAWHIKDILAINPNDAVKAYVAHEHYVAEFMEPIYGVVAMIPCDRLWSWLAETLSPDNV
PNNLYDFWISDNQGWSGTYSLENFVNSWFAAHPKQYEWESALKAYRGSMLGEVGDFRAALE

>XP_043984437.1 uncharacterized protein LOC122838113 [Gambusia affinis]

MNMESVRFCWVWTCLLLTHPCGSTAHSEHMHDVRSPGAACPDSWKWSLSSAPPGSPDVYEELWLENLDLAQQTLQLPFLQHMQLGDLQADRYASFLIQDIWYLAEVT
DLLGRAAQRSDQADDLKAFLTGRHQSYDRFKTTMLQSFNLNGVSNIKAVPAMKAYLQEYQRVLEEEEPIMMVVSLLPCSRLWVWLARQLSVGYGNAYWTWKSSNMD
GKPGKFRDLLNKHLKEKKDVQRAKEIFRKQMQNEFNFFKESLEK

>XP_036380957.1 uncharacterized protein LOC118775244 [Megalops cyprinoides]

MRSALIFSLCIWFVSASAPQNVCYLKGNQQACDLYEDLWQKNLDIAGDVLNSKFIQTMQSGSLPVHCYITFTLQDIYYLEEVTDMLKTLSAKVEKPEDLKTFFQERYKSY
SDYTSDVLGICSLTDKSIIKPQAAIKNYTDAYRDVMNTQDPIYFAIAMLPCSRLWTHLAEKLQMDKNNAYYEFKADNMRHPEKHFKPLLDKYQSTIDVTLAHAIFRSQMK
HERAFFQSAVP

>NP_001314821.1 uncharacterized protein LOC100332348 precursor [Danio rerio]

MKTAATFLALCFLGYSYISLGSCVEDVYEYLWQKNKDLAVQTLNLDFLRQMESGSLQAERYVNFTIQDIGYVLAVTKMLKRMSAEVSQPDDIRDFMKGRFASYKSFGEL
LLNMYFFKAEPPIQRIPALRNYLLYYRYLMLEEPIYFVVGLLPCARLWVWLANNLNIPPTNAYFTWKVDNMGGHPEKHYKALLNKYLNTADKVAKANAVFRDQMQNE
YKFFLTF

Table S2: List of 48 BtTenA representative proteins from diverse organisms obtained from a BLASTP search using BtTenA as a query sequence

	Organism	Classification or Common name	Locus	Length	Score (maximum)	E-value	Identities	Similarities
1	*B. thetaiotaomicron* VPI-5482	Bacteroidetes	AAO78252	246	514	0	246/246(100%)	246/246(100%)
2	*Bactroides congonensis*	Bacteroidetes	WP_195662386	252	295	2e-97	140/236(59%)	173/236(73%)
3	*Phocaeicola vulgatus*	Bacteroidetes	WP_229532965	250	280	2e-91	139/236(59%)	171/236(72%)
4	*Bacteroides thetaiotaomicron* dnLKV9	Bacteroidetes	EOR97517	252	276	5e-90	139/236(59%)	171/236(72%)
5	*Bacteroides faecis*	Bacteroidetes	MCE8942323	247	271	4e-88	134/234(57%)	166/234(70%)
6	*Prevotella copri*	Bacteroidetes	WP_217327177	246	263	1e-84	130/241(54%)	161/241(66%)
7	*Acinetobacter* sp. BIGb0196	γ-Proteobacteria	WP_166782516	247	187	3e-57	91/215(42%)	135/215(62%)
8	*Acinetobacter* sp. MYb10	γ-Proteobacteria	WP_105712671	230	174	2e-52	83/199(42%)	126/199(63%)
9	*Aeromonas rivuli*	γ-Proteobacteria	WP_224431244	220	184	5e-54	95/214(44%)	126/214(58%)
10	*Aquimarina aggregata*	Bacteroidetes	WP_066308905	237	184	4e-54	96/214(45%)	130/214(60%)
11	*Chloroherpeton thalassium*	Green sulfur bacteria / *Chlorobiota*	WP_012499220	234	182	3e-53	97/215(45%)	128/215(59%)
12	*Aeromonas encheleia*	γ-Proteobacteria	WP_242378554	247	179	1e-51	97/214(45%)	123/214(57%)
13	*Pirellula staleyi*	Planctomycetota	WP_012909371	234	173	9e-50	88/213(41%)	125/213(58%)
14	*Rhodophyticola porphyridii*	α-Proteobacteria	WP_121897937	251	172	3e-49	95/215(44%)	118/215(54%)
15	*Tenacibaculum litoreum*	Bacteroidetes	WP_125344594	230	167	3e-47	84/211(40%)	120/211(56%)
16	*Microcystis aeruginosa*	Cyanobacteria	NCQ92070	239	155	8e-43	87/220(40%)	127/220(57%)
17	*Gracilariopsis chorda*	Red seaweed	PXF48067	254	150	7e-41	81/214(38%)	117/214(54%)
18	*Candidatus Entotheonella* factor	Candidatus	ETW93821	211	149	1e-40	72/203(35%)	116/203(57%)
19	*Stigmatella aurantiaca*	Myxobacteria	WP_002614061	248	135	7e-39	71/214(33%)	114/214(53%)
20	*Chondrus crispus*	Irish Moss	XP_005710530	244	145	6e-39	81/208(39%)	116/208(55%)
21	*Acanthaster planci*	Crown-of-thorns starfish	XP_022093498	257	134	6e-35	74/226(33%)	114/226(50%)
22	*Patiria miniata*	Bat starfish	XP_038050170	258	132	4e-34	69/199(35%)	107/199(53%)
23	*Strongylocentrotus purpuratus*	Purple sea urchin	XP_003730491	258	131	3e-33	74/209(35%)	110/209(52%)
24	*Dreissena polymorpha*	Zebra mussel	KAH3819073	239	125	2e-31	70/224(31%)	115/224(51%)
25	*Oceanospirillum multiglobuliferum*	γ-Proteobacteria	WP_078745630	237	118	1e-30	63/209(30%)	107/209(51%)
26	*Mizuhopecten yessoensis*	Yesso scallop	XP_021377140	229	116	4e-28	66/232(28%)	120/232(51%)
27	*Burkholderia pseudomallei* MSHR1153	BetaProteobacteria	AIV52139	240	114	2e-28	62/185(34%)	94/185(50%)
28	*Branchiostoma belcheri*	Belcher's lancelet	XP_019617645	275	114	1e-26	70/219(32%)	108/219(49%)
29	*Mytilus coruscus*	Korean mussel	CAC5425173	235	112	1e-26	67/214(31%)	105/214(49%)
30	*Acropora digitifera*	Acropoid Coral	XP_015760767	252	108	7e-25	75/239(31%)	120/239(50%)
31	*Erpetoichthys calabaricus*	Reedfish	XP_028657201	243	106	5e-24	65/206(32%)	110/206(53%)

32	*Pocillopora damicornis*	Cauliflower coral	XP_027049566	236	107	1e-24	60/189(32%)	95/189(50%)
33	*Pecten maximus*	Great scallop	XP_033749938	230	107	1e-24	70/226(31%)	112/226(49%)
34	*Mercenaria mercenaria*	Hard clam	XP_045192634	231	103	2e-23	64/234(27%)	113/234(48%)
35	*Dendronephthya gigantea*	Soft coral	XP_028412868	271	102	5e-26	71/236(30%)	114/236(48%)
36	*Polypterus senegalus*	Senegal bichir	XP_039608874	223	101	2e-22	61/206(30%)	106/206(51%)
37	*Exaiptasia diaphana*	Brown anemone	XP_020903754	265	100	1e-21	59/186(32%)	90/186(48%)
38	*Protopterus annectens*	West African lungfish	XP_043938478	237	99.4	1e-21	70/205(34%)	103/205(50%)
39	*Hypomesus transpacificus*	Delta smelt	XP_046871482	231	97.1	1e-20	63/198(32%)	98/198(49%)
40	*Lepisosteus oculatus*	Spotted gar	XP_015217116	232	95.9	3e-20	63/205(31%)	101/205(49%)
41	*Nematostella vectensis*	Starlet sea anemone	XP_001636921	263	89.4	1e-17	69/240(29%)	110/240(45%)
42	*Polyodon spathula*	American paddle fish	XP_041096159	242	89.0	1e-17	63/198(32%)	95/198(47%)
43	*Mytilus galloprovincialis*	Mediterranean mussel	VDI43265	269	88.6	3e-19	50/197(25%)	89/197(45%)
44	*Scleropages formosus*	Asian arowana	XP_018610467	240	85.5	3e-16	57/207(28%)	91/207(43%)
45	*Cyprinodon tularosa*	White Sands pupfish	XP_038153220	249	84.0	1e-15	62/199(31%)	97/199(48%)
46	*Gambusia affinis*	Mosquito fish	XP_043984437	257	81.3	1e-15	61/213(29%)	97/213(45%)
47	*Cyprinus carpio*	Common carp	KTF92611	220	73.6	5e-15	53/205(26%)	92/205(44%)
48	*Danio rerio*	Zebrafish	NP_001314821	223	72.4	9e-15	53/198(27%)	89/198(44%)

```
Acropoid_coral           -----------------------------M-----------NRDR---------QQGT---K-----------WQ------L----------------KKLRKMRVN-------------------TSF-LP------------A-------------------------ET--------
Cauliflower_coral        -------------------------------------------------------------------------------------------------------MDVTP------------------SSQ-IP---------------------------------------S--------
Sea_anemone              -------------------------------------------------------------------------------------------------------MQDS-------------------TRR-YP------------K-------------------------TSRRMLFEID
Brown_anemone            -------------------------------------------------------------------------------------------------------MQVE-------------------TMR-YP------------K-------------------------TSGRMLVEND
Starlet_sea_anemone      -----------------------------M-----------GGRRTNFKIINIPRSGV---K------------P------A----------------DKLVGIQKS-------------------TAQPLH------------A-------------------------PS--------
Soft_coral               -----------------------------M---------------------------T---S-----------SQ------A----------------ILLLTMVVS-LTN-----------IFCYAPQCFQ------------N-------------------------EF--------
Spotted_gar              --------------------------------------------------------------------------------------------------------------------------MKSFMVLSYFCAW--SVFALDPK-------------------------EN--------
American_paddle_fish     ---------------------------------------------------------------------------------------------------------------MQRVKKDSPARMNSFVIFTLLCTW--SVFASSPR-------------------------DK--------
West_African_lungfis     --------------------------------------------------------------------------------------------------------------------------MKPGLVFLVISAGIISV-KLEPY-------------------------IQ--------
Delta_smelt              --------------------------------------------------------------------------------------------------------------------------MADI------------------------------------------------------
Asian_arowana            --------------------------------------------------------------------------------------------------------------------------MNSFLFLTFSG-----V-CYSLV-------------------------DS--------
Mosquito_fish            -------------------------------------------------------------------------------------------------------------MN-----------MES---VRFCW-----V-WTCLLLTHPCGSTAHSEHMHDVRSPGAACPDS--------
White_Sands_pupfish      --------------------------------------------------------------------------------------------------------------------------MET---VKLSC-----V-VLGLLWMFSSIS-AHSEDIHDNQ---ESCSGN--------
Common_carp              ------------------------------------------------------------------------------------------------------------------------------------------------------------------------------------
Zebrafish                --------------------------------------------------------------------------------------------------------------------------MKT---AA-TF-----L-ALCFL-------------------------GY--------
Reedfish                 -----------------------------M---------------------------M---T-----------RM------S----------------ILIL----S-LLA-----------MRS-MVWSID------------I-------------------------QD--------
Senegalus_bichir         -------------------------------------------------------------------------------------------------------------MQ-----------LES-SVFSY-------------------------------------------------
Stigmatella_aurantia     -----------------------------M---------------------------T---S-----------RL------I----------------LD-------------------------------T------------P-------------------------RK--------
Candidatus_Entotheon     ------------------------------------------------------------------------------------------------------------------------------------------------------------------------------------
Burkholderia_pseudom     ---------------------------------------------------------M---K-----------PL------V----------------TR-------------------------------T------------P-------------------------RR--------
Oceanospirillum_mult     -----------------------------M---------------------------F---N-----------SL------L----------------SR-------------------------------S------------P-------------------------R---------
Crown_of_thorns_star     ---------------------------------------------------------M---S-----------VW-----------------------QK-------------------------------G------------L-------------------------KN--------
Bat_starfish             ---------------------------------------------------------M---SS----------LR-----------------------KK-------------------------------S------------I-------------------------KN--------
Purple_sea_urchin_       ---------------------------------------------------------M---S-----------AL-----------------------KR-------------------------------S------------P-------------------------RN--------
Lancelet                 ---------------------------------------------------------M---A-----------TS------T----------------KR-------------------------------S------------R-------------------------YN--------
Yesso_Scallop            ------------------------------MALRNPRLSLH----------------V---H-----------DI------A----------------K---------------------------------------------R-------------------------KV--------
Great_scallop            ------------------------------MALRNPRLSSH----------------V---N-----------GK------F----------------K---------------------------------------------K-------------------------FV--------
Zebra_Mussel             --------------------------------------------------------------------------M------W----------------KT-------------------------------S------------P-------------------------RC--------
Korean_mussel            --------------------------------------------------------------------------M------W----------------KS-------------------------------S------------P-------------------------RC--------
Mediterranean_mussel     ---------------------------------------------------------M---N-----------GM------C-----IVLFILAFASIGT-------------------------------C------------Q-------------------------SV--------
BT_3146_VPI_5482         -----------------------------M---------------------------NDFKN-QWLRKRTFAIPA------S----------------RL-------------------------------T------------G-------------------------R---------
Bacteroides_congonen     -----------------------------M---------------------------D--MN-KWYIRRDFSVVA------E----------------RL-------------------------------G------------R-------------------------R---------
Bacteroides_thetaiot     -----------------------------M---------------------------N--MN-KWYIRRDFSVAA------E----------------RL-------------------------------G------------R-------------------------R---------
Phocaeicola_vulgatus     ------------------------------------------------------------MN-KWYIRRDFSVAA------E----------------RL-------------------------------G------------R-------------------------R---------
Bacteroides_faecis       ------------------------------------------------------------MN-KWYIRRDFSVAA------A----------------KM-------------------------------G------------K-------------------------R---------
Prevotella_copri         ------------------------------------------------------------MM-KWYQKRDFSIKV------PLSFQM-----------KL-------------------------------G------------K-----------------------------------
Acinetobacter_sp._BI     -----------------------------M---------------------------T---N-ISFFPRSFNITQ------E----------------EI-------------------------------E------------K-------------------------LQ--------
Acinetobacter_sp._MY     ------------------------------------------------------------------------------------------------------------------------------------------------------------------------------------
Aeromonas_rivuli         -----------------------------M---------------------------F---S-----------PRRLRFDNL----------------ML-------------------------------D------------Q-------------------------HG--------
Aeromonas_encheleia      -----------------------------M---------------------------F---S-----------PRRLQFDNM----------------LL-------------------------------E------------R-------------------------HG--------
Chloroherpeton_thala     -----------------------------M---------------------------K---N-----------PRRLVLTAE----------------FI-------------------------------A------------K-------------------------HG--------
Aquimarina_aggregata     -----------------------------M---------------------------K---N-----------NRRIELDKS----------------FL-------------------------------V------------E-------------------------YK--------
Tenacibaculum_litore     -----------------------------M---------------------------R---N-----------NRATLLDQT----------------FL-------------------------------T------------E-------------------------HK--------
Pirellula_staleyi        -----------------------------M---------------------------K---S-----------PRRIVLTDE----------------II-------------------------------A------------R-------------------------HN--------
Rhodophyticola_porph     -----------------------------M---------------------------R---G-----------NRRLSDISD----------------II-------------------------------E------------K-------------------------HQ--------
Microcystis_aerugino     -----------------------------M---------------------------R---N-----------NRSLIDHDY----------------LI-------------------------------K------------K-------------------------HK--------
Red_algae                --------------MTLTATLAPPRLARTM---------------------------R---N-----------PR------A----------------LT-------------------------------E------------T-------------------------PE--------
Red_Seaweed              MNSARSCFHSGNPIITLKHALNCP---RTV---------------------------S---V-----------TF------S----------------IA-------------------------------E------------K-------------------------HN--------
```

Acropoid_coral	------------------L---------------KSA-------WP---SFSQE--LFNN-------E--TSVKIRNA
Cauliflower_coral	------------------L---------------KQE-------G--SFSSA--LFND-------P--KSEIICNL
Sea_anemone	HF-PL--EELYTSPDDVD----------------SPL-------T--TFTQD--LFNS-------Q--ASVIIREA
Brown_anemone	LFLPFFQEERCTSYDEIDL---------------DNA-------K--SFSQD--LFNS-------E--ASVMIRDI
Starlet_sea_anemone	------------------A----------APFP-ENK-------A--SFTQK--LFEH-------P--KSQAIVDE
Soft_coral	------------------L---------------NKK-------A-IQGHRLKDSNTGL--TFAQS--LFED-------P--FSVNVRNA
Spotted_gar	------------------V--------------VNTK-------H--CDRHG--V----------S------------------------------------LYEKLWK-NNMDIARD
American_paddle_fish	------------------W---------------TSK-------H--CSSES--S----------S------------------------------------LYDVLWD-DSQVIADK
West_African_lungfis	------------------S---------------NYK-------S--ESSNE--VYQQ-------T------------------------------------LYEKLWE-SGLDIADQ
Delta_smelt	-----------------------------------EK-------H---IYDDLWN-SSFDIVRQ
Asian_arowana	------------------A---------------HVK-------W--TPGQD--V-----E-DSSS------------------------------------FYEDLWK-SNIDIANA
Mosquito_fish	------------------W-----------------K-------W--SLSSAPPG-----SPD---------------------------------------VYEELWL-ENLDLAQQ
White_Sands_pupfish	------------------A-----------------K-------W--CSSSAHKV-----DPD---------------------------------------YYEELWE-KNLDLAKQ
Common_carp	--M--SYSQA--V-----D-D---------------------------------------VYEYLWE-QNKDIANK
Zebrafish	------------------S-----------------Y-------I--SLGSC--V-----E-D---------------------------------------VYEYLWQ-KNKDLAVQ
Reedfish	------------------P---------------SAK-------W--TSPGK--CSQD-------S------------------------------------VYDQLWD-KSQDIVGE
Senegalus_bichir	--W--QYVAE--CSHD-------S------------------------------------IYDQLWD-KSQDIVGE
Stigmatella_aurantia	------------------S------SAQL-----STA-------N--RLD-TP-----------A--PQRG----------E-------S------------------------------------LSKELWA-RTQDVAQE
Candidatus_Entotheon	--MAAHLWN-ASQDLAQA
Burkholderia_pseudom	------------------I------QLDH-----PSL-------A--KLD-VPT----------S--PPPA----------N-------S------------------------------------ITSQLWE-ACYSLAQD
Oceanospirillum_mult	------------------S------KTPL-----KSI-------N-----------------L----------------R----PQLQ-TDA----------S--P-------------S-------S------------------------------------LYDQMWA-ATTLYQQT
Crown_of_thorns_star	------------------I------RSPR-----STA-------A-----------------MLEHLPQTVIDR-LAAKHPAKLTKVP-VAD----------GRYE-------------E-K-----E------------------------------------LSEWLWE-SSQVQAQL
Bat_starfish	------------------V------RSPR-----RIA-------A-----------------CVEHLPQSFVDQLLAPKRKAKLMKVADTAE----------G--G-------------E-R-----E------------------------------------LAEWLWE-SSQAQAQE
Purple_sea_urchin_	------------------V------RSPR-----STA-------A-----------------MVKHLPSAVAALLQPGKGPAKLTKFD-TPD----------G--E-------------KWM-----T------------------------------------LTEFLWN-ISQVQAQE
Lancelet	------------------V------QSPR-----NRL-------A--------------NPRVLKAFLQATIAFQKAGKK----DEVD-SPDVLRATAQQRVA--D-------------E-K-----T------------------------------------LSERLWD-QNLDLVDA
Yesso_Scallop	------------------V---------------KTD-------L--E-------P------------------------------------LSQYLWE-QTESTRQK
Great_scallop	------------------K---------------KTD-------A--E-------P------------------------------------LSENLWN-QTEGIRQE
Zebra_Mussel	------------------V---------------SGA-------A--V-------KKLLQCTLSDKPCN--------------------DYDFCEWLWT-ETKDTRIK
Korean_mussel	------------------V---------------SDA-------A--V-------RELQPFTLSDKPNT--------------------DFDFCELMWT-ETKETRIK
Mediterranean_mussel	------------------Q---------------STA-------R--T-------PECLHFSSKTSGNNHISQGFGKRIPKPTGTGHECSHNLSHWLWH-ETENFRTW
BT_3146_VPI_5482	----------------------------------LTTLKSDVPAA--D-------S------------------------------------LFWKLWN-GSLDTAVQ
Bacteroides_congonen	----------------------------------GQLLSSDAPPP--D-------A------------------------------------LFWEMWQ-ECEPIARQ
Bacteroides_thetaiot	----------------------------------GQFLSSDAPPP--D-------A------------------------------------LFWEMWQ-ECEPIARQ
Phocaeicola_vulgatus	----------------------------------GQFLSSDAPPP--D-------A------------------------------------LFWEMWQ-ECEPIARQ
Bacteroides_faecis	----------------------------------GIALSSDEPPA--D-------A------------------------------------LFWEMWN-ECEDIARQ
Prevotella_copri	-------------------------------------LQQRDASA--S-------T------------------------------------LFQEMWD-ECEDIAKA
Acinetobacter_sp._BI	------------------F---------------STTLSVEPPPK--N-------S------------------------------------LFQIMWD-ECLPIAEK
Acinetobacter_sp._MY	--MWD-ECLPIAEK
Aeromonas_rivuli	------------------L---------------SSA----PPPT--D-------S------------------------------------LFWRLWQ-PCIPIAEK
Aeromonas_encheleia	------------------L---------------SSA----PPPV--D-------S------------------------------------LFWRLWQ-PCLPIAQK
Chloroherpeton_thala	------------------L---------------STA----PPPE--N-------S------------------------------------LFWKMWN-ACESIAQA
Aquimarina_aggregata	------------------L---------------STA----PPPA--N-------S------------------------------------LFWKMWD-DCMQIADD
Tenacibaculum_litore	------------------L---------------STA----PPPA--D-------S------------------------------------LFWNMWN-ACQEIADK
Pirellula_staleyi	------------------L---------------CTD----PPPS--D-------S------------------------------------LFWLLWN-TCIQTAQQ
Rhodophyticola_porph	------------------L---------------STA----PPPA--D-------S------------------------------------LFWKMWN-GVQDVATD
Microcystis_aerugino	------------------L---------------NTN----PPPP--N-------S------------------------------------LFWKMWNAGGEWIAQE
Red_algae	------------------LLLQLLG---------PRT----PPRV--D-------S------------------------------------LFWTLWS-KSADVACK
Red_Seaweed	------------------L---------------SKT----SPPE--H-------S------------------------------------LFWRLWH-QNKHYAKK

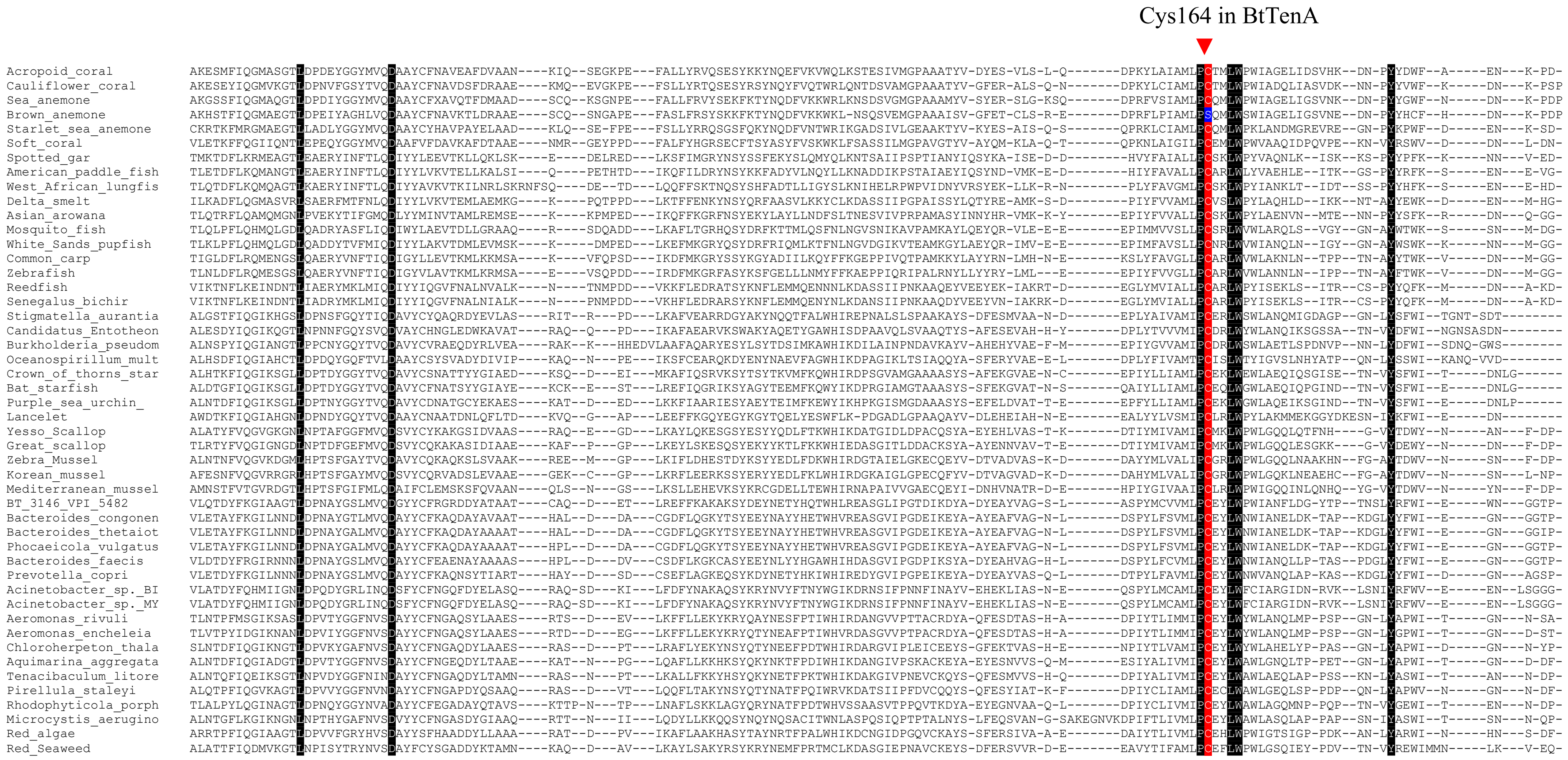
Cys164 in BtTenA

Glu229 in BtTenA

```
Acropoid_coral          -G--H-KSR-LEEFVD----YF-F---NA----------GDKAKSLVIFHQGLVNELNFF---RN----A--------------------------------------------------------------------------------------------------CDQTL--YYYSS
Cauliflower_coral       -G--H-KSH-LEMFVD----HF-FVQVGP----------EERQKCLSIFQEGLVNEVNFF---RS----A--------------------------------------------------------------------------------------------------CDQTL--FYYSS
Sea_anemone             SG--K-KST-VERFVN----RC-FTK--P----------EDKQKAEEIFLEGMVNELNFF---RD----A--------------------------------------------------------------------------------------------------CGEGL--RY--N
Brown_anemone           SG--K-KST-VEMFVD----SF-FKTLDE----------XEKEKALMIFLESMVNEVNXF---RD----A--------------------------------------------------------------------------------------------------CGEML-------
Starlet_sea_anemone     ----H-PSH-LERFID----YF----LRD----------EKVRDYLDEFLEGMTNELNFF---RD----A--------------------------------------------------------------------------------------------------CGEEL--YYYTK
Soft_coral              ----G-YSG-AQTFAN----KF----FSE----------GDKDASQIIFNEGIINELNFF---RS----A--------------------------------------------------------------------------------------------------CGEDPVDYDFGN
Spotted_gar             ----NSKKH-YENLLE----KY----RCN----------IDESKAQSLFRLQMAHEREFF---ST----S--------------------------------------------------------------------------------------------------------------
American_paddle_fish    ----HPEKH-YKDLLE----NH----RSE----------IDESKAKNIFQKHMKHEYNFF---RS----S--------------------------------------------------------------------------------------------------------------
West_African_lungfis    ----GSKNN-YESLLE----RH----RLK----------ISERQAKKIFQTHMKLEMQFF---AE----A--------------------------------------------------------------------------------------------------------------
Delta_smelt             ----DPRTA-YQPLLD----KY----ACS----------INRETAKTLFRKQMSHEQSFF---MA----SITARPADALLGVCRSTVDHRHIFMPFSY----------------------------------------------------------------------------------
Asian_arowana           ----NPAKH-FKGLLD----GY----QHK----------MNKKTAHQIFRQQMEHEKNFF---QS----S----------------------------YTEKPILF--------------------------------------------------------------------------
Mosquito_fish           ----KPGK--FRDLLN----KHL---KEK----------KDVQRAKEIFRKQMQNEFNFF---KE----S----------------------------LEK-------------------------------------------------------------------------------
White_Sands_pupfish     ----KPEK--YKDLLS----KYL---TA-----------KNFEKANKIFRNQMGNELQFF---KA----S----------------------------LNQ-------------------------------------------------------------------------------
Common_carp             ----HPEKT-YRALLN----KYL---KTP----------EKVKKANTVFRAQMQNEHDFF--------------------------------------FSS-------------------------------------------------------------------------------
Zebrafish               ----HPEKH-YKALLN----KYL---NTA----------DKVAKANAVFRDQMQNEYKFF--------------------------------------LTF-------------------------------------------------------------------------------
Reedfish                ----KSRDH-YEKLLQ----EY----KDK----------IDMDEALKIFQIQMRHELAFF---QSVDPKN--------------------------------------------------------------------------------------------------------------
Senegalus_bichir        ----KSRDH-YEKILQ----EY----KDK----------IDMNLGLNIFQTQMKHELAFF---QSVDSKN--------------------------------------------------------------------------------------------------------------
Stigmatella_aurantia    ------GACRLEAFVD----AH----AHH----L------DEGRALSVYRTCMLGECNFF---RS----A--------------------------------------------------------------------------------------------------------------
Candidatus_Entotheon    ------GAHQMENVVD----TH----AAQ----L------DEDTALHVYRTAMLGEVNFF---RS----A--------------------------------------------------------------------------------------------------------------
Burkholderia_pseudom    ------GTYRLENFVNSWFAAH----PKQ----Y------EWESALKAYRGSMLGEVGDF---RA----A--------------------------------------------------------------------------------------------------------------
Oceanospirillum_mult    ------APKSMADFIN----AH----ANE----M------NQQLAIDIFSKCMQFECNMF---LS----A--------------------------------------------------------------------------------------------------------------
Crown_of_thorns_star    ------GSSKLAHFID----AY----ADE----YG----VDLQKAKDIYHGAMQGEVNFF---AS----A--------------------------------------------------------------------------------------------------------------
Bat_starfish            ------GSHKLANFIN----EH----TES----YG----VRPEVAKGIYKTAMQCEVEFF---RS----A--------------------------------------------------------------------------------------------------------------
Purple_sea_urchin_      ------GSHTLANYID----EN----AEK----FG----VDLKKAMKIYQQGMQCEVDFF---TS----G--------------------------------------------------------------------------------------------------------------
Lancelet                ------GSYKSAEEVE----EV----VDG----CSLSGLINEEKANQIYRKCMYGEVNFF---KS----A--------------------------------------------------------------------------------------------------------------
Yesso_Scallop           --TYD-GYKQLDEFVD----TA----GTT----------IDKETALKVYSRCMEGEYEFF---NS----A--------------------------------------------------------------------------------------------------------------
Great_scallop           --TYD-GYKELDKFVD----DA----AIT----GS----IDAQKALKVYAYCMSGELAFF---GS----V--------------------------------------------------------------------------------------------------------------
Zebra_Mussel            --TSE-GYKKLEVRVN----AA----FSN----QE----IDEKKALSIYSKCMNGEASFF---GS----V--------------------------------------------------------------------------------------------------------------
Korean_mussel           --NSK-GYKTLQDMVN----AA----VSG----RK----IDKKKAMDIYSKCMNGEAGFF---GS----V--------------------------------------------------------------------------------------------------------------
Mediterranean_mussel    --TYD-GYKKLEVMTN----EF----YAK----GL----IDKDKALHYYAKSIEGEAAFF---DS----V--------------------------------------------------------------------------------------------------------------
BT_3146_VPI_5482        ----N-GAYQMGNMLE----QY----RDK----------IDEDKAVEIFNTAMNYELKVF---TS----S------------------------------------T-------------------IL--TTIENGK-------------------------------------------
Bacteroides_congonen    ----D-GAYQMANMLE----SY----RRQ----------IDEAKAKEIFRIALQHELKVF---TA----A------------------------------------T-------------------LLKSELLWQRK-----------------------NSSLQ-R-------------
Bacteroides_thetaiot    ----D-GAYQMANMLE----SY----RRQ----------IDEAKAKEIFRIALQHELKVF---TA----A------------------------------------T-------------------LLKSELLWQRK-----------------------NSILQ-R-------------
Phocaeicola_vulgatus    ----D-GAYQMANMLE----SY----RRQ----------IDEAKAKEIFRIALQHELKVF---TT----A------------------------------------T-------------------LLKSELLWQRK-----------------------NSILQ-R-------------
Bacteroides_faecis      ----D-GAYQMANMLE----NY----RTQ----------MDEKQAKEIFHTAMRHELEVF---ST----A------------------------------------T-------------------ELKNNALWLRK-----------------------NTF-----------------
Prevotella_copri        ----T-GALQMESMLE----LY----RAD----------IDENKAKEIFRTAMEFELKVF---TS----A------------------------------------T-------------------IL--NLSYYGK-----------------------KEI-----------------
Acinetobacter_sp._BI    ----Q-GAYLMGNILE----KY----RNQ----------LDINKCIRIYMESMKHELNVF---TN----A------------------------------------T-------------------LK--SEFMHIK-------------------------------------------
Acinetobacter_sp._MY    ----Q-GAYLMGNILE----KY----RNQ----------LDINKCIRIYMESMKHELNVF---TN----A------------------------------------S-------------------LK--SEFMHIKQPFQIKSLKLQFNQRVTQKHKKL--------------------
Aeromonas_rivuli        ----S-GAYAMGNFLD----QY----QKEHPGA------IDEDKAMSIYSTAMNFEWKNF---LS----A------------------------------------T---------------------------------------------------------Q-P-------------
Aeromonas_encheleia     ----S-GAYAMGNFLD----QY----QRAHPGA------IDENKAQSIYSTAMNFEWQNF---LS----A------------------------------------T---------------------------------------------------------Q-S-------------
Chloroherpeton_thala    ----D-GAYAMGNFLN----DY----QIAHPDE------IDENTAIEIYTQAMTYEQRNF---SA----A------------------------------------TAS-------------------------------------------------------Q-S-------------
Aquimarina_aggregata    ----E-GAYAMGNFLN----EY----QKKHV--------INESIAIDIYTKAMTYEYLNF---MT----A------------------------------------T---------------------------------------------------------K-TK------------
Tenacibaculum_litore    ----G-GAYAMGDFLS----DY----QKEKP--------IDEKLATQLYTEAITYEYQNF---NT----A------------------------------------L---------------------------------------------------------N-S-------------
Pirellula_staleyi       ----S-GAYAIGNFLQ----SY----QIANPGV------VDQSLSLKIYSQAMIYEQQNFAAALS----A------------------------------------T---------------------------------------------------------D---------------
Rhodophyticola_porph    ----S-GAYAMGNFLD----SY----MTQNPGV------VDPPYAQLIYGFAQIYEYQNF---AA----A------------------------------------T--GTQPNDAAHYGIGKPAI--------------------------------------PPS-------------
Microcystis_aerugino    ----D-GAYAMGNFLQ----EY----ITLNR--------IDENLATVIYLTAMEYEYSNF---SS----A------------------------------------T---------------------------------------------------------QPG-------------
Red_algae               ----G-SAFALGNVID----NF----ELRNPRI------LCRDKALYIYRKAMVYEWKNF---EN----A--------------------------------------------------------------------------------------------------------------
Red_Seaweed             ----G-NAFAAGNFLV----DF----ESVYGRE------IDHDKAGRIYRQAMVYEWQNF---AT----A------------------------------------C---------------------------------------------------------S-Y-------------
```

```
Acropoid_coral          -FNV------------------------------------
Cauliflower_coral       -FQE------------------------------------
Sea_anemone             -FGKLTVGFGRK----------------------------
Brown_anemone           ---ELV-----H----------------------------
Starlet_sea_anemone     EFQM------------------------------------
Soft_coral              -KNE--------TAPIV-----------------------
Spotted_gar             ----------------------------------------
American_paddle_fish    ----------------------------------------
West_African_lungfis    ----------------------------------------
Delta_smelt             ----------------------------------------
Asian_arowana           ----------------------------------------
Mosquito_fish           ----------------------------------------
White_Sands_pupfish     ----------------------------------------
Common_carp             ----------------------------------------
Zebrafish               ----------------------------------------
Reedfish                ----------------------------------------
Senegalus_bichir        ----------------------------------------
Stigmatella_aurantia    -----------------CKQDPLLLEPARITATA------
Candidatus_Entotheon    -----------------CRQ-PLLEMPG----TS------
Burkholderia_pseudom    -----------------------LE---------------
Oceanospirillum_mult    -----------------CNQ-PPV----------------
Crown_of_thorns_star    ----------------------------------TAEVP-
Bat_starfish            ----------------------------------TIDVV-
Purple_sea_urchin_      ----------------------------------TIEEVN
Lancelet                -----------------CDE-PLL----------IIPPQ-
Yesso_Scallop           -------------------Q-LMM----------------
Great_scallop           -------------------Q-HK-----------------
Zebra_Mussel            ----------------------PI----------------
Korean_mussel           ----------------------PI----------------
Mediterranean_mussel    ----------------------KP----------------
BT_3146_VPI_5482        ----------------------------------------
Bacteroides_congonen    ----------------------------------------
Bacteroides_thetaiot    ----------------------------------------
Phocaeicola_vulgatus    ----------------------------------------
Bacteroides_faecis      ----------------------------------------
Prevotella_copri        ----------------------------------------
Acinetobacter_sp._BI    ----------------------------------------
Acinetobacter_sp._MY    ----------------------------------------
Aeromonas_rivuli        ----------------------------------------
Aeromonas_encheleia     ----------------------------------------
Chloroherpeton_thala    ----------------------------------------
Aquimarina_aggregata    -----------------FNT-QLL----------------
Tenacibaculum_litore    ----------------------------------------
Pirellula_staleyi       ----------------------------------------
Rhodophyticola_porph    ----------------------------------------
Microcystis_aerugino    ----------------------------------------
Red_algae               ----------------------------------------
Red_Seaweed             ----------------------------------------
```

Figure S1: Alignment of 48 representatives of the BtTenA family using PRANK. The TenA conserved cysteine residues are indicated with a red arrow and the conserved glutamate residues are indicated with a blue arrow. Amino acid residues conserved in the 48 proteins are marked in black.

```
Zebrafish                       --------------------------MKTAATFLALCFLGYSYI-----------SLGSC   23
Acinetobacter sp. MYb10         ------------------------------------------------------------   0
BT_3146                         MNDFKNQWLRKRTFAIPASR-L--------------------TGRLTTL-----KSDVPA   34
Prevotella copri                ----MMKWYQKRDFSIKVPLSF--------------------QMKLGKL-----QQRDAS   31   BtTenA subfamily
Zebra mussel                    -------MWKTSPRCVSG----------------------AAVKKLLQC---TLSDKPCN   28
Bat starfish                    ----MSSLRKKSIKNVRSPRRIAACVEHLPQSFVDQLLAPKRKAKLMKV----ADTAEGG   52
Purple sea urchin               -----MSALKRSPRNVRSPRSTAAMVKHLPSAVAALLQPGKGPAKLTKF----DTPDGEK   51
Zea mays                        ------------------------------------------------------MDGGGV   6
Cushaw squash                   ------------------------------------------------MADPKTRAQLAG   12
Arabidopsis thaliana            ----------------------------------------------------------ME   2
Black cottonwood                ------------------------------------------------------MD-PVE   5    TenA_E subfamily
Adzuki bean                     ------------------------------------------------MMEQKSKE-EEK   11
Soybean                         -------------------------------------------------MEEKAKA-EQK   10
Velvet bean                     -------------------------------------------------MEEKAKA-EEK   10
Rhizobium leguminosarum         ----------------------------------------------------------MA   2
Helicobacter pylori             ------------------------------------------------------------   0
Bacillus subtilis               ------------------------------------------------------------   0
Lactococcus lactis              ------------------------------------------------------------   0
Candida metapsilosis domain     ----------TKLGHGNGPL-------------NHNITPCTQITRITDMADNN-ILKFDD   36   TenA_C subfamily
Klebsiella pneumoniae           ----------------------------------------------------------MI   2
Aureobasidium melanogenum domain ------------------------------------------------------------   0


Zebrafish                       VEDVYEYL----WQKNKDLAVQTLNLDFLRQMESGSLQAERYVNFTIQDIGYVLAVTKML   79
Acinetobacter sp. MYb10         -------M----WDECLPIAEKVLATDYFQHMIIGNLDPQDYGRLINQDSFYCFNGQFDY   49
BT_3146                         ADSLFWKL----WNGSLDTAVQVLQTDYFKGIAAGTLDPNAYGSLMVQDGYYCFRGRDDY   90
Prevotella copri                ASTLFQEM----WDECEDIAKAVLETDYFKGILNNNLDPNAYGSLMVQDAYYCFKAQNSY   87   BtTenA subfamily
Zebra mussel                    DYDFCEWL----WTETKDTRIKALNTNFVQGVKDGMLHPTSFGAYTVQDAVYCQKAQKSL   84
Bat starfish                    ERELAEWL----WESSQAQAQEALDTGFIQGIKSGLLDPTDYGGYTVQDAVYCFNATSYY   108
Purple sea urchin               WMTLTEFL----WNISQVQAQEALNTDFIQGIKSGLLDPTNYGGYTVQDAVYCDNATGCY   107
Zea mays                        DTATTA----AWMEKHRHMYERATRHPFTVSIRDGTVDMSAFKRWLSQDYLFVREFVAFI   62
Cushaw squash                   GMTATD----SWIRKHRLIYTDATRHPFVLSIRDGTVDLSAFRTWVEQQCEFLRSFTAFV   68
Arabidopsis thaliana            KRGVID----TWIDKHRSIYTAATRHAFVVSIRDGSVDLSSFRTWLGQDYLFVRRFVPFV   58
Black cottonwood                KNGVID----RWVKKHLVLYTGATRHPFILSIRDGTIDFSSFKRWLGQDYIFVREFVPFA   61   TenA_E subfamily
Adzuki bean                     KVGVIE----TWLRKHPLPFKGATRHPLILSIRDGSIDIASFKSWLAQDYLFVRAFVPFV   67
Soybean                         KIGMTE----TWLKKHRLLYNGATRHPLIISIRDGTINTASFKTWLAQDYLFVRAFVPFV   66
Velvet bean                     KIGMTE----VWIRKHRLLYDGATRHPLILTIRDGTINIPSFKTWLAQDYLFVRAFVPFV   66
Rhizobium leguminosarum         AESLSD----RILRENDAVLGAMLSHRFVEDVKNDRLSKEAFQRYLVYEGAFVDSAISIF   58
Helicobacter pylori             -MQVSQ----YLYQNAQSIWEDCISHPFVQGIGRGTLERDKFRFYIIQDYLFLLEYAKVF   55
Bacillus subtilis               -MKFSE----ECRSAAAEWWEGSFVHPFVQGIGDGTLPIDRFKYYVLQDSYYLTHFAKVQ   55
Lactococcus lactis              -MLFTE----LAREKSSIFWERSFEHPFIQGLVSGELAPEVFRYYLLQDRYYLEHFSKLY   55
Candida metapsilosis domain     NHTFIDYL--TSHPKVKQTWQNYVEHPFVKRLAENNLPFENFFYYLKQDYHYLIIYARMH   94   TenA_C subfamily
Klebsiella pneumoniae           VPAFSQGLYGRLRQLAAADWQRYVAHPFVQQLASGTLAENAFRRYLTQDYLFLIHFARSY   62
Aureobasidium melanogenum domain -GGFVDWL--LEREDVQQVWKEFTQHEFVEKMGDGTLPVERFKFYMVQDYLYLTQFARAN   57
```

```
Zebrafish                        KRMSAEVSQPDD---IRDFMKGRFAS---YKSFGELLLNMYFFKA----EPPIQRIPALR      129
Acinetobacter sp. MYb10          ELASQRAQSDKI---LFDFYNAKAQS---YKRYNVYFTNYWGIKD----RNSIFPNNFIN      99
BT_3146                          ATAATCAQ-DET---LREFFKAKAKS---YDEYNETYHQTWHLRE----ASGLIPGTDIK      139
Prevotella copri                 TIARTHAY-DSD---CSEFLAGKEQS---YKDYNETYHKIWHIRE----DYGVIPGPEIK      136   BtTenA subfamily
Zebra mussel                     SVAAKREE-MGP---LKIFLDHESTD---YKSYYEDLFDKWHIRD----GTAIELGKECQ      133
Bat starfish                     GIAYEKCK-EST---LREFIQGRIKS---YAGYTEEMFKQWYIKD----PRGIAMGTAAA      157
Purple sea urchin                EKAESKAT-DED---LKKFIAARIES---YAEYTEIMFKEWYIKH----PKGISMGDAAA      156
Zea mays                         ASVLLKCCKQE-DSSDMEIILGGVASISDEISWFKNEATVWGVDL----ASVS-PLKANL      116
Cushaw squash                    ASVLVKAWKESDDRADEEVILGSLATLNDEFAWFKKEALKRDIDL----TKIV-PQNATA      123
Arabidopsis thaliana             ASVLIRACKDSGESSDMEVVLGGIASLNDEIEWFKREGSKWDVDF----STVV-PQRANQ      113
Black cottonwood                 ASVLLKASKNSDDNSDMEVILSGLASLSDEISWFKQEAAKWDVPL----SDVV-VHKSNQ      116   TenA_E subfamily
Adzuki bean                      ASVLIKAWKESDESGDMEVILGGVASLEDEISWFKREASKWGISL----SEVV-PQQANK      122
Soybean                          ASVLIKAWKESDCSGDMEVILGGMASLEDEISWFKTEANKWGISL----SDVV-PQQANK      121
Velvet bean                      ASVLIKAWKDSDDSGDMEVILGGMASLEDEISWFKREANKWGILL----SEVD-PQQANI      121
Rhizobium leguminosarum          AYAAATANTMPQ---KRWLIAVLDALANEQIAYFERTFASRCIDP----SSFDTGIAEVE      111
Helicobacter pylori              ALGVVKAYDEAV---MREFSNAIQDILNNEMSIHNHYIRELQITQ-TELQNAR-PTLANK      110
Bacillus subtilis                SFGAAYAKDLYT---TGRMASHAQGTYEAEMALHREFAELLEISE-EERKAFK-PSPTAY      110
Lactococcus lactis               FLIAKKSASEEI---QAHLRLNAEELKNGEIFVREGFFKELEIGE-QEILETP-VAPTAY      110
Candida metapsilosis domain      GLLASKAPTYQQ---THAAATIIG-EIITEVEHHKKRLKAYNVDYERDIEKLQ-PGKACI      149   TenA_C subfamily
Klebsiella pneumoniae            ALLVSKLRTLPE---MRAAAASMN-AILNELPLHVGYCAQWGISE-PEMAAQP-EAPETL      116
Aureobasidium melanogenum domain ALAGYKAKTLEG---VAASASIVT-HIHTETKLHVSECLELGVTM-DELRNSE-EHQACT      111


Zebrafish                        NYLLYYRYLM-LEE-PIYFVVGLLPCARLWVWLANN-----LN--IPPTNAYFTWKVDNM      180
Acinetobacter sp. MYb10          AYVEHEKLIASNEQ-SPYLMCAMLPCEYLWFCIARG-----IDNRVKLSNIYRFWVEENL      153
BT_3146                          DYADYEAYVAGSLA-SPYMCVVMLPCEYLWPWIANF-----LDGYTPTNSLYRFWIEWN-      192
Prevotella copri                 EYADYEAYVASQLD-TPYLFAVMLPCEYLWNWVANQ-----LAPKASKDGLYYFWIDGN-      189   BtTenA subfamily
Zebra mussel                     EYVDTVADVASKDD-AYYMLVALIPCGRLWPWLGQQ-----LNAAKHNFGAYTDWVNSNF      187
Bat starfish                     SYSSFEKGVATNSQ-AIYLLIAMLPCEQLWGWLAEQ-----IQPGINDTNVYSFWIEDNL      211
Purple sea urchin                SYSEFELDVATTEE-PFYLLIAMLPCEKLWGWLAQE-----IKSGINDTNVYSFWIEDNL      210
Zea mays                         EYHRFLRSFTEPEISYAVAVTTFWTIETVYQDSF--GFC--IQDGNKTPPELLGTCQRWG      172
Cushaw squash                    GYSRFLESLMRPEMEYTVAITALWAIEAVYNESF--AYC--MEDGSKTPLELREACERWG      179
Arabidopsis thaliana             EYGRFLEDLMSSEVKYPVIMTAFWAIEAVYQESF--AHC--LEDGNKTPVELTGACHRWG      169
Black cottonwood                 NYCRFLESLMLPAVEYSVVFTALWAIETVYQESF--SHC--LEDGSKTPPELLEACKRWG      172   TenA_E subfamily
Adzuki bean                      KYCGLLESLMSPDVEYTVAITAFWVIEVVYQESF--AYC--IGEGSKTPQELKETCERWG      178
Soybean                          NYCGLLESLMSPDAEYTVAITAFWAIETVYQESF--AHC--IEEGSKTPPELKETCVRWG      177
Velvet bean                      NYCRLLESLMSPDVEYTVAITALWAIEAVYQESF--AHC--IEEGSKTPSELKETCARWG      177
Rhizobium leguminosarum          TFRAGMLEIARQGG-FLDTVAAMFAAEWMYWTWCRQ-----AASRPISDPLLKEWVDMHV      165
Helicobacter pylori              SYTSYMLAEGVKGS-IKEATAAVLACGWSYLVIA--QNLSQIPNALEHA-FYGHWIKGYS      166
Bacillus subtilis                SYTSHMYRSVLSGN-FAEILAALLPCYWLYYEVG--EKLLH--CDPGHP-IYQKWIGTYG      164
Lactococcus lactis               HYVSHMYRQLIDGS-VNSAIAGMLPCAWLYHEIG--LRLIE--VGSPNP-LYQKWIETYA      164
Candida metapsilosis domain      EYCDYLLDIGQKED-FLGIKVALAPCLHGYAEAGAYGKKLRTENPVEIPEVYSGWLDEYA      208   TenA_C subfamily
Klebsiella pneumoniae            NYTRYVLDIGHSGD-ALDLLVALMPCVAGYAEIG--LGLLQHPATRLDDNPYASWIRNYG      173
Aureobasidium melanogenum domain AYSRYILDIGASED-WLALQIAMFPCLLGYHHIAKRLSLLQDPSAPKNANRYRQWIDNYI      170
```

```
Zebrafish                          G-GHPEKHYKA--LLNKYL--N-TADKVAKANAVFRDQMQNEYKFFLTF-----------     223
Acinetobacter sp. MYb10            S-GGGQGAYLMGNILEKY----RNQLDINKCIRIYMESMKHELNVFTNASLK--------     200
BT_3146                            G-GTPNGAYQMGNMLEQY----RDKIDEDKAVEIFNTAMNYELKVFTSSTIL--------     239
Prevotella copri                   A-GSPTGALQMESMLELY----RADIDENKAKEIFRTAMEFELKVFTSATIL--------     236   BtTenA subfamily
Zebra mussel                       D-PTSEGYKKLEVRVNAAF--SNQEIDEKKALSIYSKCMNGEASFFGSVPI---------     235
Bat starfish                       G-GS----HKLANFINEHT--ESYGVRPEVAKGIYKTAMQCEVEFFRSATID--------     256
Purple sea urchin                  P-GS----HTLANYIDENA--EKFGVDLKKAMKIYQQGMQCEVDFFTSGTIE--------     255
Zea mays                           SAGFRQYCQSLQSIVDRCL-ANAPADAVQSAEEAFVRVLELEIGFWDMSSSRS-------     224
Cushaw squash                      NEGFGNYCNTLKKIVDRRL-EMAAGEISKKTEVALLRVLECEVEFWNMSRDGPQQTI---     235
Arabidopsis thaliana               NDGFKQYCSSVKNIAERCL-ENASGEVLGEAEDVLVRVLELEVAFWEMSRGGQ-------     221
Black cottonwood                   SEGFGEFCRSLKKIVNRCL-EKAPDEELKKAEVTFLHVLELEIEFWDMSHGGPK------     225   TenA_E subfamily
Adzuki bean                        SEAFAKYCQSLQNIVNRRL-QKASDEELKKAERMFLNVLEYEVDFWNMSRGNV-------     230
Soybean                            NEAFGKYCQSLQNIANRCL-QKASDEELKKAEVMLLSVLEHEVEFWNMSRGNV-------     229
Velvet bean                        NEGFGKYCQSLQKLANRRL-QKASDEEIKKAEVMLLSVLEHEVEFWNMSRGN--------     228
Rhizobium leguminosarum            DPNFAAQAQWLKNELDVAG-ETLEEDEKARLSAIFDRAMQLEIDFHDAPYL---------     215
Helicobacter pylori                SKEFQACVSWNINLLDSLT-HASSKQEIEKLKDIFITTSKYEYMFWDMAYQS--------     217
Bacillus subtilis                  GDWFRQQVEEQINRFDELA-ENSTEEVRAKMKENFVISSYYEYQFWGMAYRKEGWSDSAI     223
Lactococcus lactis                 SEEATVQIAKECDIINRLY-QESDEQEQEQMLEAFLISSKMEADFWEMALVLEKW-----     218
Candida metapsilosis domain        SDWYSKADKEGRLALNALVRDGITQGRADELVDIFNKVSQLEINFWSEVLDI--------     260   TenA_C subfamily
Klebsiella pneumoniae              DEGYLQGVSAALALLETVWQQRGSEARITELSEIFTTATRLEAQFWQMGLNAAAETRA--     231
Aureobasidium melanogenum domain   ADDYTQAVGKGTELVEGHV-SKQSPSRIEELVKIFIHATKMECGFWDMGMGA--------     221

Zebrafish                          -------------------------------     223
Acinetobacter sp. MYb10            -SEFMHIKQPFQIKSLKLQFNQRVTQKHKKL     230
BT_3146                            -TTIENGK-----------------------     246
Prevotella copri                   -NLSYYGKKEI--------------------     246   BtTenA
Zebra mussel                       -------------------------------     235
Bat starfish                       -VV----------------------------     258
Purple sea urchin                  -EVN---------------------------     258
Zea mays                           -------------------------------     224
Cushaw squash                      -------------------------------     235
Arabidopsis thaliana               -------------------------------     221
Black cottonwood                   -------------------------------     225   TenA_E
Adzuki bean                        -------------------------------     230
Soybean                            -------------------------------     229
Velvet bean                        -------------------------------     228
Rhizobium leguminosarum            -------------------------------     215
Helicobacter pylori                -------------------------------     217
Bacillus subtilis                  KEVEECGASRHN-----------------G-     236
Lactococcus lactis                 -------------------------------     218
Candida metapsilosis domain        -------------------------------     260   TenA_C
Klebsiella pneumoniae              -------------------------------     231
Aureobasidium melanogenum domain   -------------------------------     221
```

NP_001131451.1 Zea mays
KAG7026352.1 Cushaw squash
Q9ASY9.3 Arabidopsis thaliana
XP_002311391.1 Black cottonwood
XP_017424346.1 Adzuki bean
NP_001238329.1 Soybean
RDX62165.1 Velvet bean
BDA08841.1 Helicobacter pylori
PDB: 1YAF_A Bacillus subtilis
WP_221971201.1 Rhizobium leguminosarum
SPS12676.1 Lactococcus lactis
KAG5417137.1 Candida metapsilosis
STR31639.1 Klebsiella pneumoniae
KAH0023325.1 Aureobasidium melanogenum

Figure S2: Clustal Omega (https://www.ebi.ac.uk/Tools/msa/clustalo/) alignment of members of the TenA_C, TenA_E and BtTenA subfamilies. Red colour indicates the active site cysteine residue in TenA_C and BtTenA (BT_3146) and the conserved glutamate residues in all subfamilies are indicated in green.

***Burkholderia pseudomallei* MSHR1153**

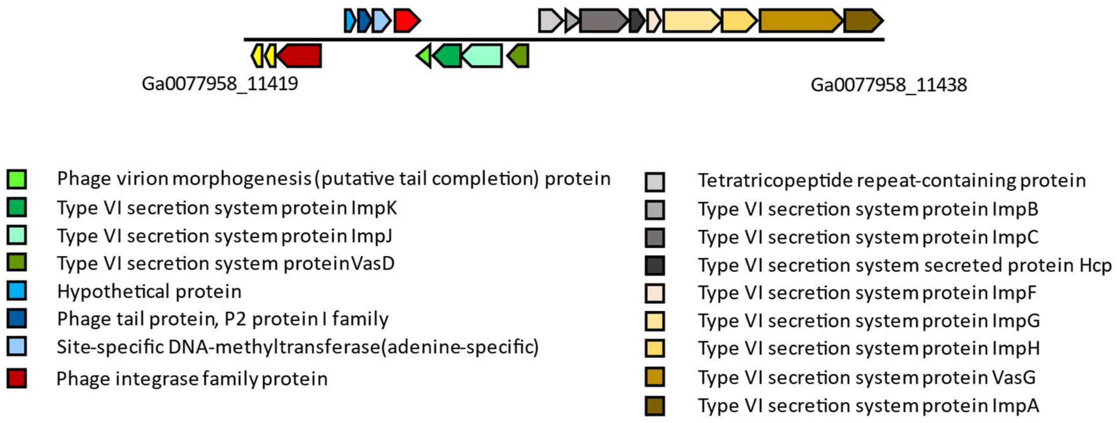

***Burkholderia pseudomallei* VB976100**

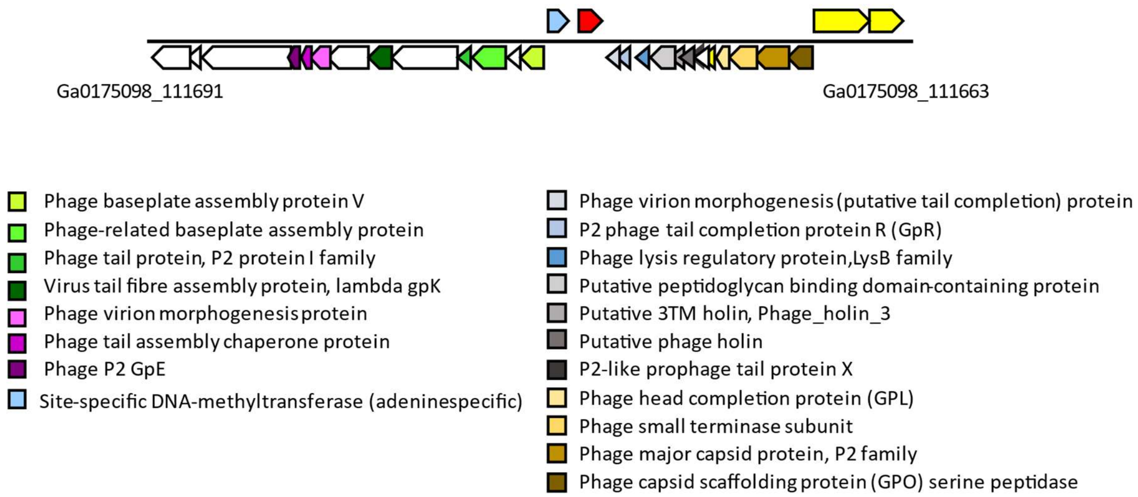

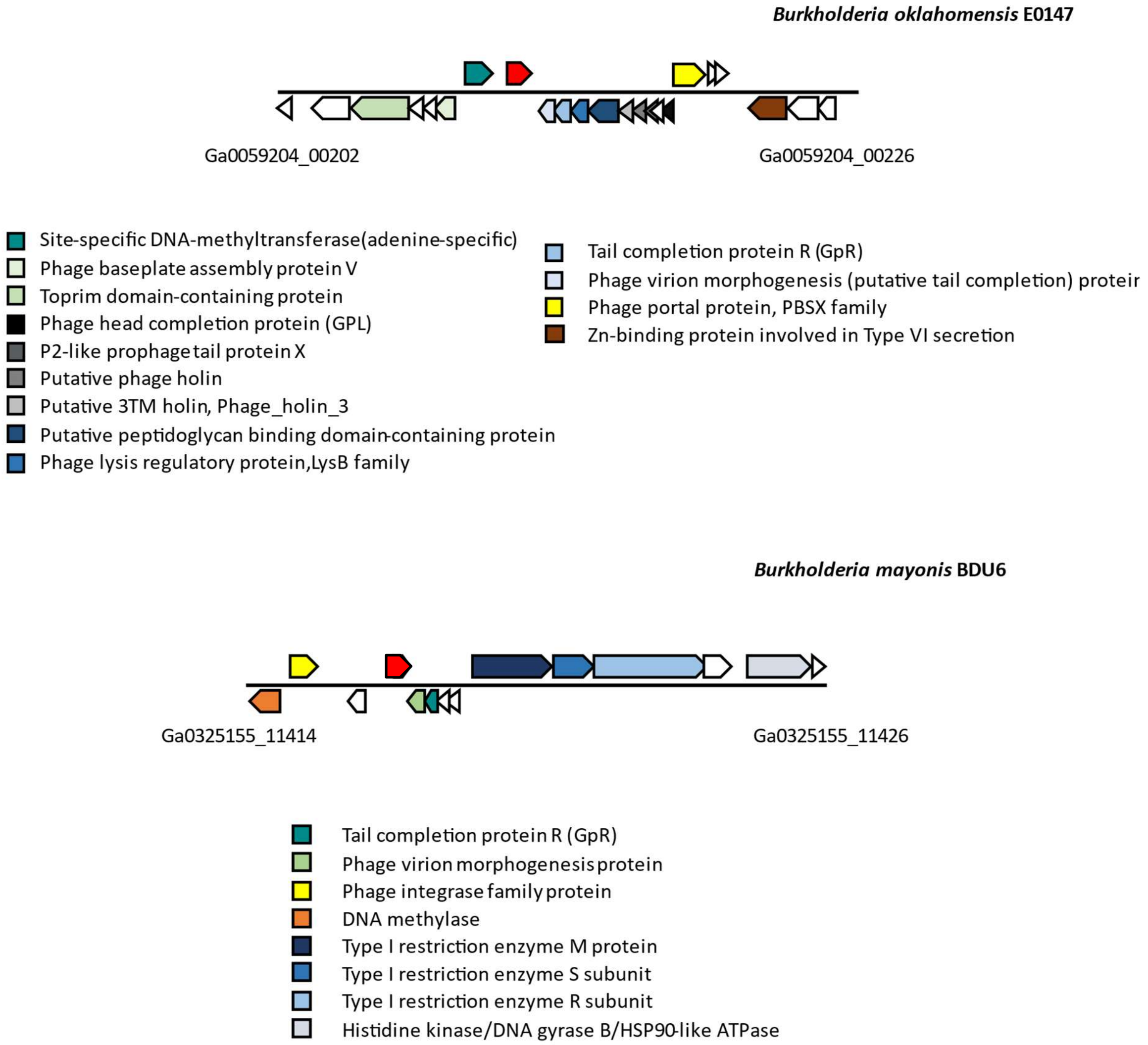

Figure S3: Bt*tenA*-like gene (in red) and comparison of neighbouring genes in *Brukholderia pseudomallei*, *B. mayonis* and *B. oklahomensis*.
